# Structure and operating principles of a monkeypox virus replisome

**DOI:** 10.1038/s41586-026-10937-2

**Published:** 2026-09-02

**Authors:** Zishuo Yu, Pradeep Sathyanarayana, Joel M. J. Tan, Side Hu, Xiaoyi Fan, Angela Gao, Philip J. Kranzusch, Joseph J. Loparo, Jonathan Abraham

**Affiliations:** 1 Department of Microbiology, Harvard Medical School, Boston, MA USA; 2 Department of Biological Chemistry and Molecular Pharmacology, Blavatnik Institute, Harvard Medical School, Boston, MA USA; 3 Department of Cancer Immunology and Virology, Dana-Farber Cancer Institute, Boston, MA USA; 4 Department of Medicine, Division of Infectious Diseases, Brigham & Women’s Hospital, Boston, MA USA; 5 Center for Integrated Solutions for Infectious Diseases, Broad Institute of Harvard and MIT, Cambridge, MA USA; 6 Howard Hughes Medical Institute, Boston, MA USA

**Keywords:** Cryoelectron microscopy, Pox virus, Single-molecule biophysics

## Abstract

Poxviruses are double-stranded DNA viruses with large genomes. Among them, monkeypox virus (MPXV) has been responsible for two recent public health emergencies as declared by the World Health Organization^1^. The MPXV polymerase comprises three subunits—a catalytic subunit (F8) and a heterodimeric processivity factor (A22 and E4). The viral polymerase must coordinate activities with the hexameric helicase–primase (E5) to initiate replication of the viral genome^2^. Although structures of MPXV E5 (refs. ^3,4^) and the polymerase^5–7^ in isolation are available, how they assemble into a functional replisome remains unclear. In isolation, E5 is in an autoinhibited conformation and has very weak helicase activity^3,4^, and the mechanism for helicase activation is unclear. Here we used cryo-electron microscopy to determine the structures of DNA-bound MPXV replisomes comprising the polymerase holoenzyme (F8, A22 and E4) and the E5 helicase hexamer. We show that, during replisome assembly, E5 undergoes large-scale conformational changes that allow two of its primase domains to interact with the polymerase F8 thumb and A22 subunit. Biochemical assays and single-molecule experiments reveal that this E5 conformational change is coupled to helicase activation and enhances primase activity. Taken together, these findings identify fundamental mechanisms governing coordinated helicase and polymerase activities during DNA replication for an important class of viral pathogens.

## Abstract (web summary)

During replisome assembly of monkeypox virus, the hexameric helicase–primase E5 undergoes large-scale conformational changes that allow two of its primase domains to interact with the polymerase F8 thumb domain and A22 subunit, activating the helicase–primase.

## Main

Since 2022, MPXV has affected over 140 countries and resulted in more than 179,000 laboratory-confirmed cases^8^. The most recent wave of infections was fuelled by the clade Ib variant, with evidence of sustained human-to-human transmission and global spread^9^. Another poxvirus is variola virus, which causes smallpox, one of the most devastating diseases known to humanity before it was eradicated.

Poxviruses replicate in the cytoplasm of host cells. Their genomes encode proteins for DNA replication and gene expression^2^ and have inverted terminal repetitions with incompletely base-paired hairpin loops connecting the two DNA strands, making a continuous polynucleotide chain^2^. The proteins that replicate the viral genome include the DNA polymerase (F8, A22 and E4) and the hexameric helicase–primase E5. The DNA polymerase of poxviruses is a family B DNA polymerase, and the catalytic subunit, F8, contains canonical fingers, palm and thumb domains, an N-terminal domain (NTD) and a 3′–5′ proofreading exonuclease (Exo) domain^5^. F8 exonuclease activity also promotes virus genetic recombination^10,11^. In the assembled polymerase holoenzyme, A22 serves as a bridge connecting F8 and E4 (refs. ^5,6,12^). The N-terminal region of viral helicase–primase E5 contains a primase domain, the enzymatic activity of which is essential for viral DNA replication^13^. The C-terminal region of E5 contains a superfamily-3 helicase and forms a hexameric ring with a central channel for ssDNA translocation in the 3′–5′ direction^14,15^. The single-stranded DNA (ssDNA)-binding protein I3 is also essential for viral replication^16,17^, as is a viral DNA or host DNA ligase^18^.

Previous studies with full-length MPXV E5 have shown that it has weak activity unless the primase domains are deleted^3,4,19^. Structures of E5 have revealed that the primase domains block the E5 ssDNA channel, suggesting an autoinhibited conformation^3,4^. How E5 transitions from an autoinhibited conformation to an activated state is unclear.

Here we determined cryo-electron microscopy (cryo-EM) structures of DNA-bound MPXV E5 helicase–primase in a complex with the DNA polymerase holoenzyme. We used biochemical assays and single-molecule experiments to show that conformational changes that occur when E5 interacts with the polymerase result in helicase activation and enhance E5 primase activity.

## MPXV replisome assembly

**Fig. 1:**
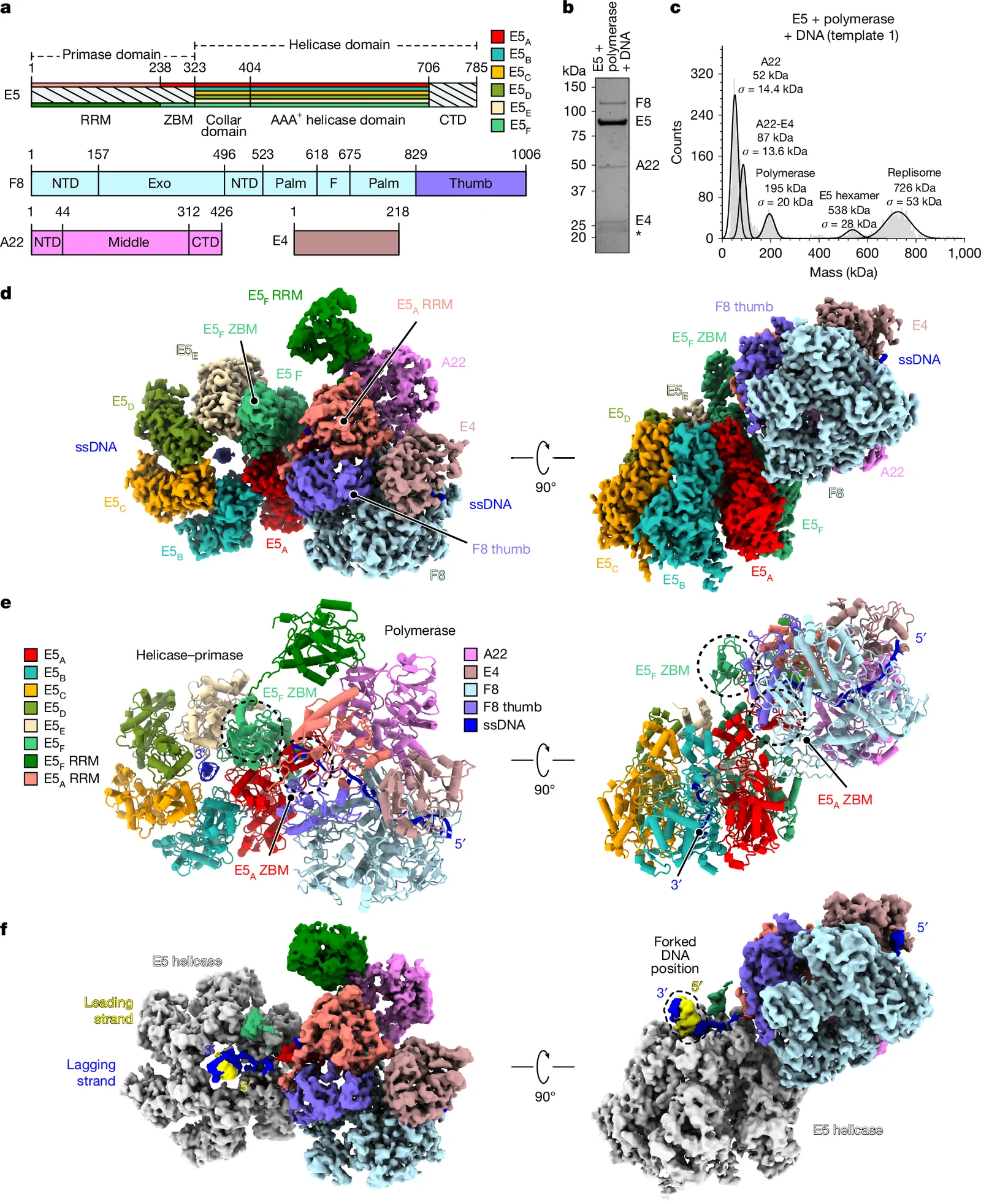
Cryo-EM structure of the MPXV replisome. **a**, The domain architecture of MPXV E5, F8, A22 and E4. For E5 protomers, the dashed boxes indicate regions that could not be resolved, probably due to flexibility. CTD, C-terminal domain; NTD, NH_2_-terminal domain; F, finger domain. **b**, SDS–PAGE analysis of MPXV E5 with polymerase and DNA (template 1) complex after purification by SEC, visualized using a stain-free gel system. The asterisk indicates a band that probably represents a degraded protein product or contaminant. The experiment was performed three times; a representative gel is shown. **c**, MP analysis of SEC-purified E5 with polymerase and DNA (template 1). The 726 kDa peak probably represents the MPXV replisome; the 538 kDa complex is consistent with the E5 hexamer; the 195 kDa peak may correspond to the polymerase holoenzyme; the 87 kDa peak may correspond to an A22–E4 subcomplex; and the 52 kDa peak may be A22 alone. Further information is provided in Supplementary Table 1. This experiment was performed twice (*n* = 2); representative data are shown. **d**,**e**, Composite cryo-EM map (**d**) and ribbon diagram (**e**) of the MPXV replisome bound to ssDNA. The overall map resolution is 4.1 Å; a composite map comprising maps focused on the helicase (3.8 Å) and primase-domain-bound polymerase (3.5 Å) post-processed with DeepEMhancer^46^ is shown. In **e**, the E5_F_ ZBM and linker to the E5_F_ RRM are shown but were not deposited in the final coordinates owing to poor density. **f**, Composite cryo-EM map of the MPXV replisome bound to forked DNA. The overall map resolution is 6.5 Å; a composite map comprising maps focused on the helicase (4.5 Å) and primase-bound polymerase (4.1 Å) post-processed with DeepEMhancer^46^ is shown. Polymerase subunits are coloured as in **e**, and the E5 helicase domains are shown in grey.

**Extended Data Fig. 1:**
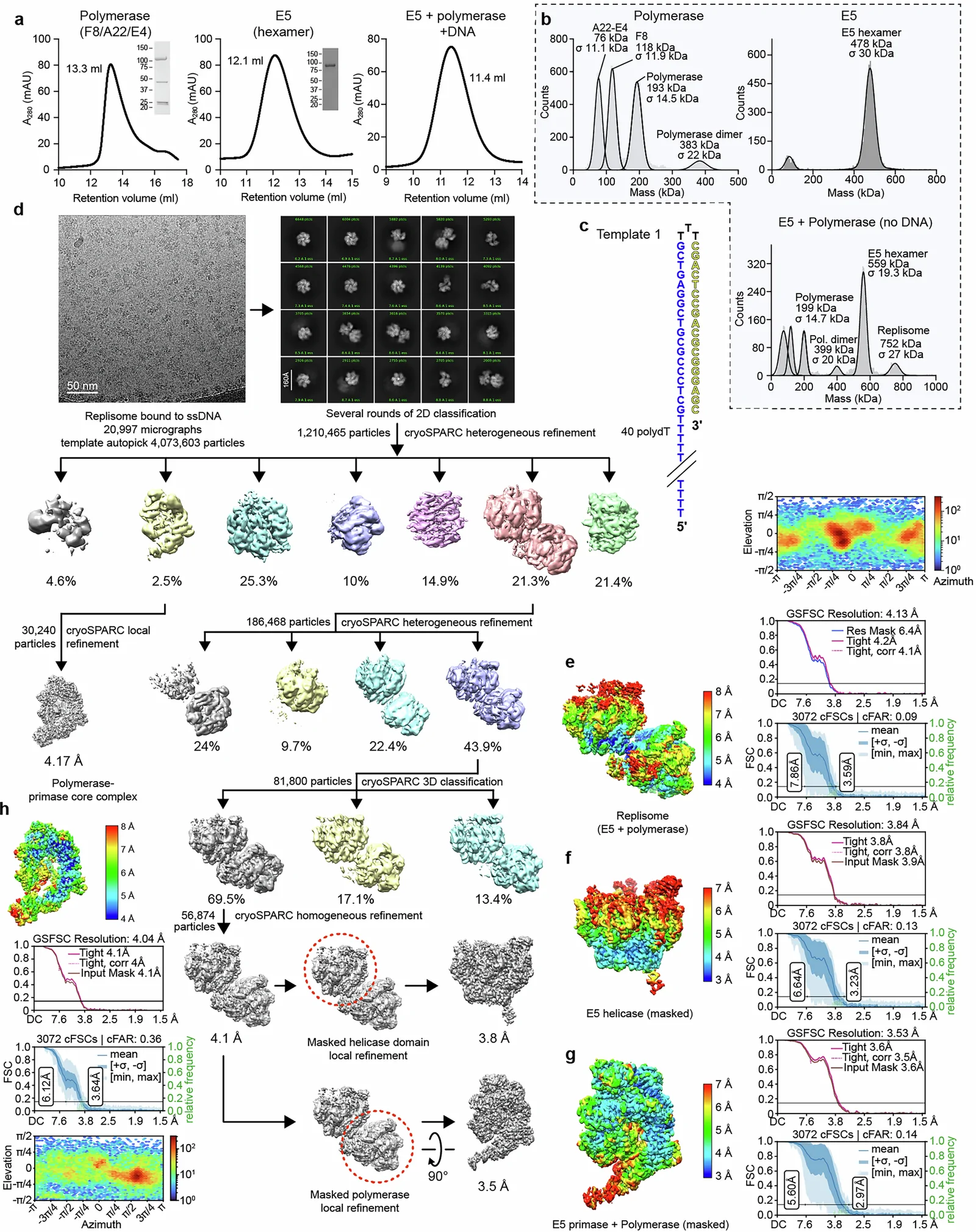
Cryo-EM reconstruction of MPXV replisome. **a**, Size-exclusion chromatography profiles of MPXV polymerase (F8/A22/E4), E5 hexamer, or complex comprising E5, polymerase, and DNA (template 1). Proteins were passed over a Superdex 200 increase column. The elution volume of the main peak is indicated for each chromatogram. A gel of the peak fraction for the E5/polymerase/DNA complex is provided in Fig. 1b. **b**, Mass photometry of the polymerase alone, E5 alone, or E5 and polymerase co-incubated without DNA. The experiment was performed twice (*n* = 2) and representative data are shown. **c**, Schematic diagram of the hairpin DNA (template 1) used to determine the structure of the ssDNA-bound MPXV replisome. **d**, Workflow used for cryo-EM data processing of the ssDNA-bound MPXV replisome. **e**–**g**, Local resolution estimation, Fourier shell correlation (FSC) curves, 3D-FSC curves and particle angular distributions of the cryo-EM reconstructions of the overall ssDNA-bound MPXV replisome at 4.1 Å resolution (**e**), masked helicase at 3.8 Å resolution (**f**), masked polymerase and E5 primase domains at 3.5 Å resolution (**g**). **h**, Local resolution estimation, FSC curves, 3D-FSC curves, and particle angular distributions of the cryo-EM reconstruction of the DNA-bound MPXV polymerase–primase core complex at 4.0 Å resolution. Maps were post-processed with DeepEMhancer^46^.

We purified E5 (residues 1–785) and the polymerase holoenzyme (F8, residues 1–1006; A22, residues 1–426; and E4, residues 1–218) in transiently transfected mammalian cells (Fig. 1a and Extended Data Fig. 1a). When examined using mass photometry (MP), polymerase samples had a substantial proportion of particles consistent with assembled holoenzyme (F8–A22–E4) heterotrimers, which would have an expected mass of 191 kDa (MP measurement of 193 with a s.d. (*σ*) of ±14.5 kDa) (Extended Data Fig. 1b and Supplementary Table 1). We also observed a smaller peak, possibly representing the autoinhibited dimers of F8, A22 and E4 also observed in a previous study^20^ (Extended Data Fig. 1b).

E5 formed hexamers consistent with an expected mass of 540 kDa (478 kDa, *σ* = ±30 kDa) (Extended Data Fig. 1b). Mixing the polymerase with E5 in the absence of DNA did not yield a substantial population of particles larger than hexameric E5 (Extended Data Fig. 1b). However, co-incubating polymerase with E5 and hairpin DNA with a 40 nucleotide-long poly-deoxythymidine (pdT) overhang (template 1) resulted in a complex that was stable when analysed by size-exclusion chromatography (SEC) (Extended Data Fig. 1a,c and Supplementary Table 2). SDS–PAGE analysis of the peak sample revealed bands for polymerase subunits (F8, A22 and E4) and E5 (Fig. 1b). MP analysis of the peak sample revealed a large proportion of particles with a mass of 726 kDa (*σ* = ±53 kDa), close to the expected mass of one copy of the polymerase and one copy of hexameric E5 along with DNA substrate (theoretical mass of 731 kDa for protein, and 25 kDa for nucleic acids) (Fig. 1c and Supplementary Table 1).

## Cryo-EM structure of the MPXV replisome

We used single-particle cryo-EM to obtain a 4.1 Å map of a complex that includes E5, polymerase and template 1 DNA (Extended Data Fig. 1c–e and Supplementary Table 3). Using masked refinement, we obtained a 3.8 Å map focusing on the hexameric E5 helicase and a 3.5 Å map focusing on the polymerase and two associated E5 primase domains (Extended Data Fig. 1f,g). We resolved in maps the helicase domains of the E5_A_–E5_F_ protomers and the primase domains of E5_A_ and E5_F_ (Fig. 1a). We did not observe density for the C-terminal domains of E5 protomers.

**Extended Data Fig. 2:**
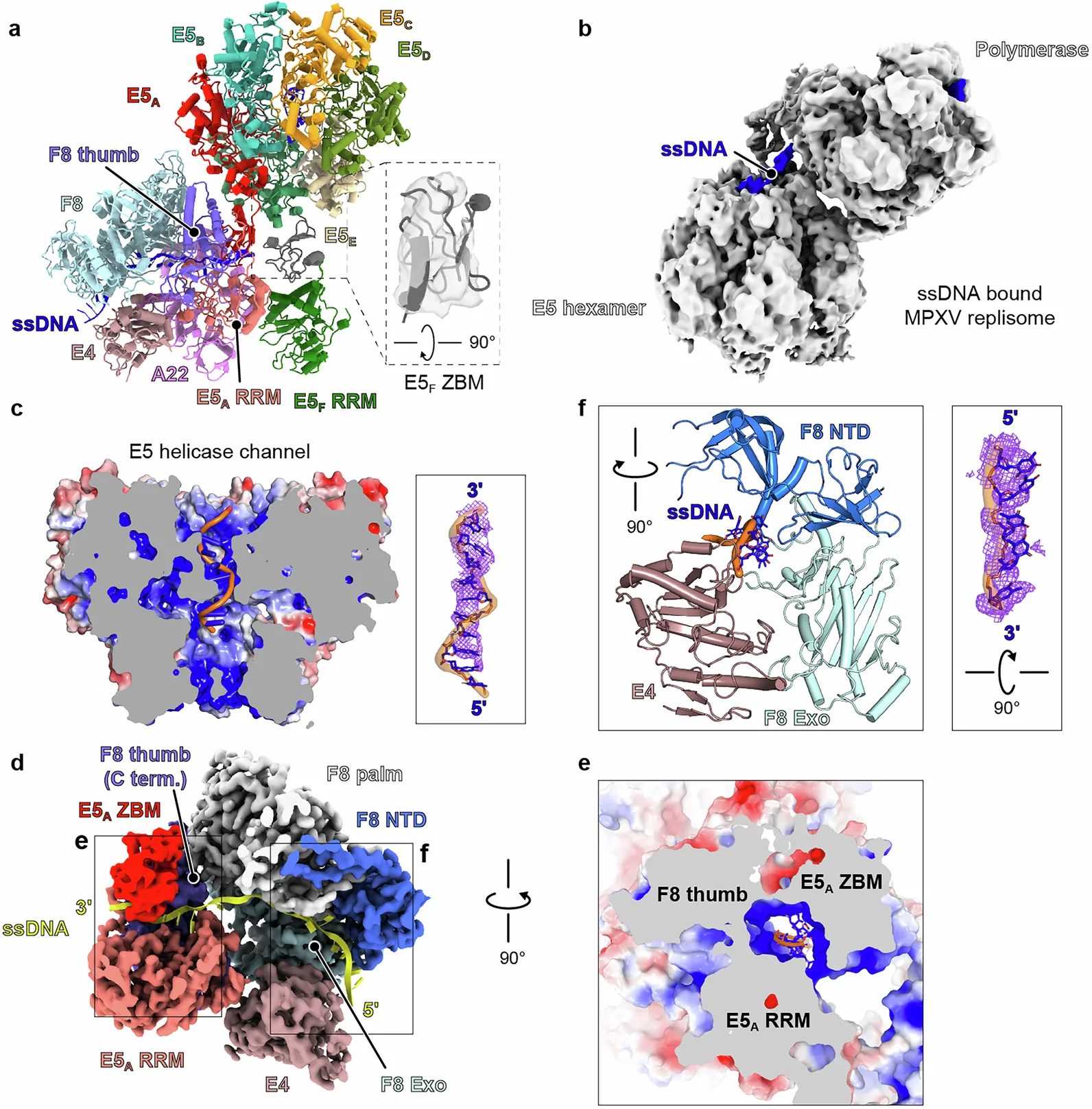
Single-stranded DNA recognition by the MPXV replisome. **a**, Ribbon diagram of the ssDNA-bound MPXV replisome. The cryo-EM density of E5_F_ ZBM, which can be observed in maps at low contour, is shown. **b**, Cryo-EM map of the ssDNA-bound MPXV replisome, showing the location of ssDNA density bridging the helicase and polymerase, which can be observed at low map contour levels in the map before post-processing (overall map resolution 4.1 Å). **c**, Electrostatic surface potential representation of the MPXV E5 helicase ssDNA channel, and cryo-EM density of ssDNA within E5 helicase. **d**, Cut-off view of the cryo-EM density of MPXV polymerase and E5_A_ ZBM and RRM shown as a surface from the map of the ssDNA-bound replisome (E5 primase and polymerase masked, 3.5 Å) post-processed with DeepEMhancer^46^. The bound ssDNA is shown as a ribbon diagram. C-term.: polymerase C-terminal region. **e**, Electrostatic surface potential representation of the ssDNA channel formed by the E5_A_ RRM and ZBM and the F8 thumb. **f**, Zoomed-in view of the ssDNA binding channel formed by E4 and F8 involving the F8 NTD and Exo domain, along with cryo-EM density for the ssDNA segment that is positioned through this region. The rotation with respect to the view shown in panel **d** is provided.

The complex can be divided into two parts: one is ring-shaped and includes the hexameric E5 helicase domains, and the other is irregularly shaped and includes the polymerase bound to the E5_A_ and E5_F_ primase domains (Fig. 1d,e). The E5 primase domain includes an RNA-recognition motif (RRM) and a zinc-binding motif (ZBM) (Fig. 1a). In the complex, the E5_A_ RRM and ZBM domains bind to the F8 thumb, and the E5_F_ RRM binds to A22 (Fig. 1d,e). We observed weak density for the E5_F_ ZBM (Fig. 1d and Extended Data Fig. 2a), suggesting flexibility.

## MPXV replisome ssDNA recognition

**Extended Data Fig. 3:**
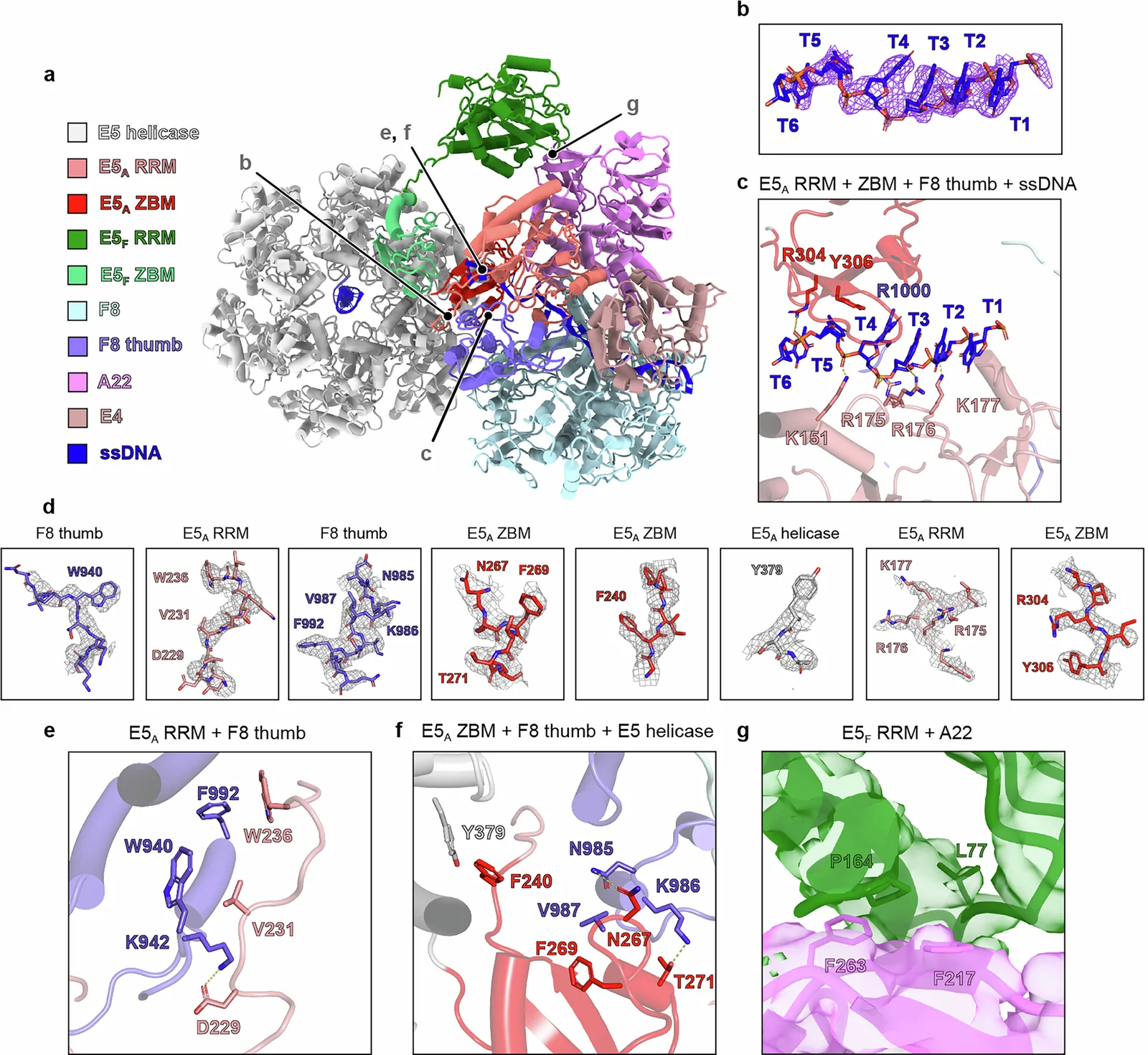
Protein-protein and protein-DNA interactions in the ssDNA-bound MPXV replisome. **a**, Ribbon diagram (left) and schematic diagram (right) of the ssDNA-bound MPXV replisome. The general location of the views shown in panels **b**, **c**, **e**, **f**, and **g** are indicated. **b**, Cryo-EM density of the T1–T6 ssDNA segment. Nucleotides are shown as sticks. **c**, Interactions between the T1–T6 ssDNA segment with the E5_A_ RRM, E5_A_ ZBM, and the F8 thumb. **d**, Cryo-EM density of the residues involved in polymerase and primase domain interactions during MPXV replisome assembly from an E5 primase and polymerase masked (3.5 Å) map post-processed with DeepEMhancer^46^. Residues are shown as sticks. **e**, Interactions between the E5_A_ RRM and F8 thumb. **f**, Interactions between E5_A_ ZBM, E5_A_ helicase, and the polymerase F8 thumb. **g**, Interactions between E5_F_ RRM and polymerase A22 subunit.

Template 1 DNA was required for efficient complex assembly as monitored by MP (Fig. 1c and Extended Data Fig. 1b), suggesting that DNA binding has a structural role in replisome assembly. We observed density for ssDNA interacting with both E5 and the polymerase, as well as weak ssDNA density bridging E5 and the polymerase (Extended Data Fig. 2b). The observed density would account for around 10 nucleotides in the ssDNA channel of the helicase, about 8 bridging nucleotides and around 17 nucleotides in the polymerase–E5_A_–E5_F_-primase complex. The map for most of the ssDNA segment was too poor in quality to unambiguously model nucleotides, and certain segments had strong density only for stacked bases (for example, ssDNA within E5) (Extended Data Fig. 2c). However, the portion of ssDNA interacting with the F8 thumb and E5_A_ RRM and ZBM (nucleotides T1–T6) had density that was clear enough to model individual bases and the phosphate backbone (Extended Data Fig. 3a–c).

We modelled a ssDNA segment as a poly-deoxythymidine (dT) strand, with the 3′ end passing through the centre of the E5 hexamer, which is a positively charged channel (Extended Data Fig. 2c). The 5′ end enters the polymerase through a positively charged tunnel to interact with the E5_A_ RRM and ZBM domains, which are held in place by contacts with the F8 thumb (Extended Data Fig. 2d,e). The ssDNA strand is positioned within the palm and through a ssDNA-binding channel formed by the F8 NTD and Exo domain (Extended Data Fig. 2f).

**Extended Data Fig. 4:**
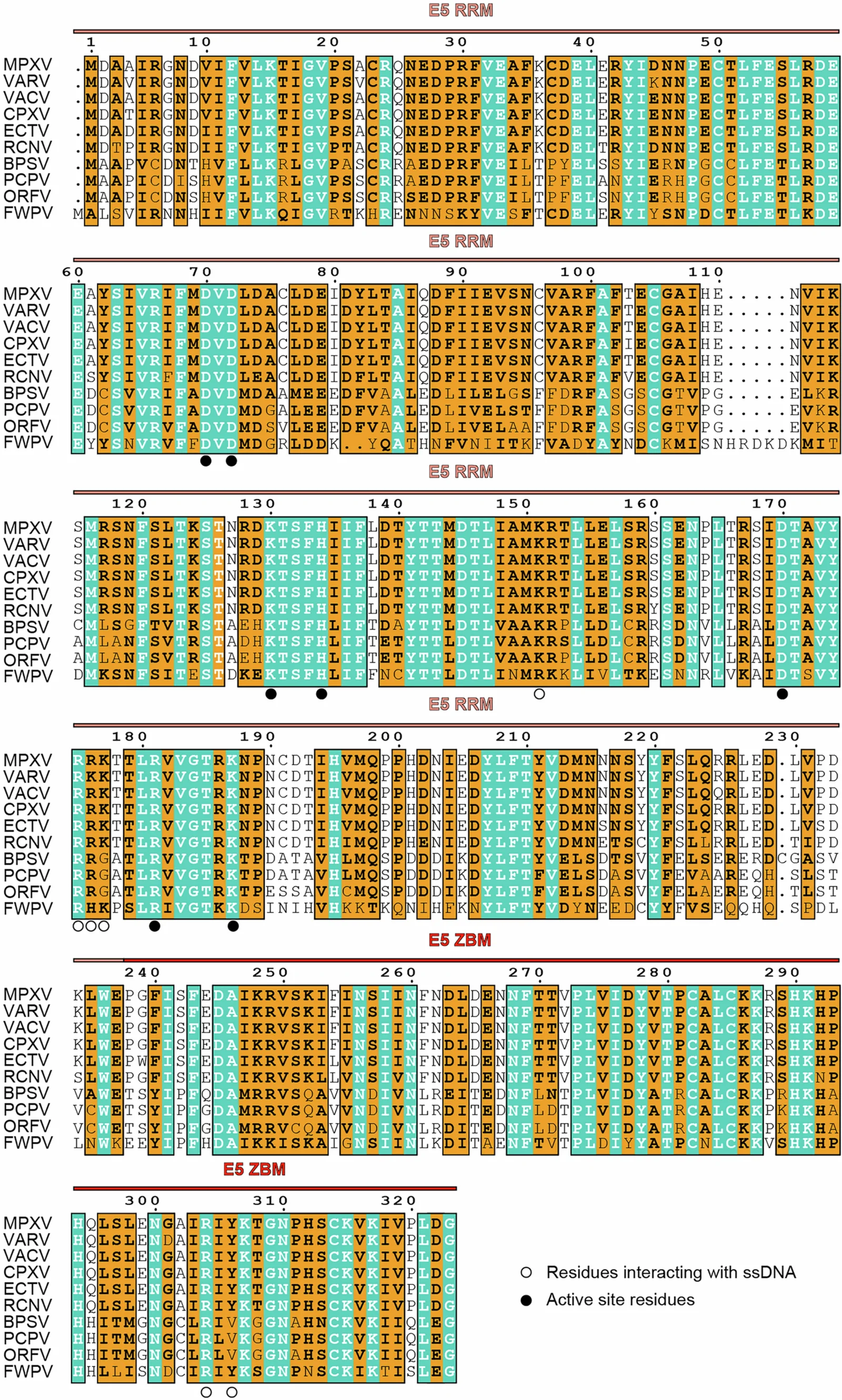
Sequence comparison of poxvirus primase domains. Amino acid sequence alignment of the primase domain, which spans E5 residues 1–323 (MPXV numbering). Conserved active site residues in the primase and residues that interact with ssDNA in the cryo-EM structure of the MPXV replisome are indicated. GenBank accession numbers are as follows: monkeypox virus (MPXV) E5 (XNX20584.1); variola virus (VARV) F5R (NC_001611); vaccinia virus (VACV) E5R (NC_006998); cowpox virus (CPXV) CPXV122 (NC_003663); ectromelia virus (ECTV) EVM094 (NC_004105); raccoonpox virus (RCNV) ACG19_gp106 (NC_027213); bovine papular stomatitis virus (BPSV) BPSVgORF068 (NC_005337); pseudocowpox virus (PCPV) gp070 (NC_013804); orf virus (ORFV) gORF068 (NC_005336); fowlpox virus (FWPV) FPV058 (NC_002188).

Four basic residues on the E5_A_ RRM (Lys151, Arg175, Arg176 and Lys177) contact the ssDNA phosphate backbone, and one F8 thumb residue (Arg1000) contacts an ssDNA base (T4) (Extended Data Fig. 3c,d). E5_A_ ZBM residues Arg304 and Tyr306 interact with ssDNA on the face opposite the one contacted by the RRM. Examination of sequence alignments of poxvirus E5 primase domains reveals that the residues contacting ssDNA are highly conserved (Extended Data Fig. 4).

## MPXV replisome forked DNA recognition

**Extended Data Fig. 5:**
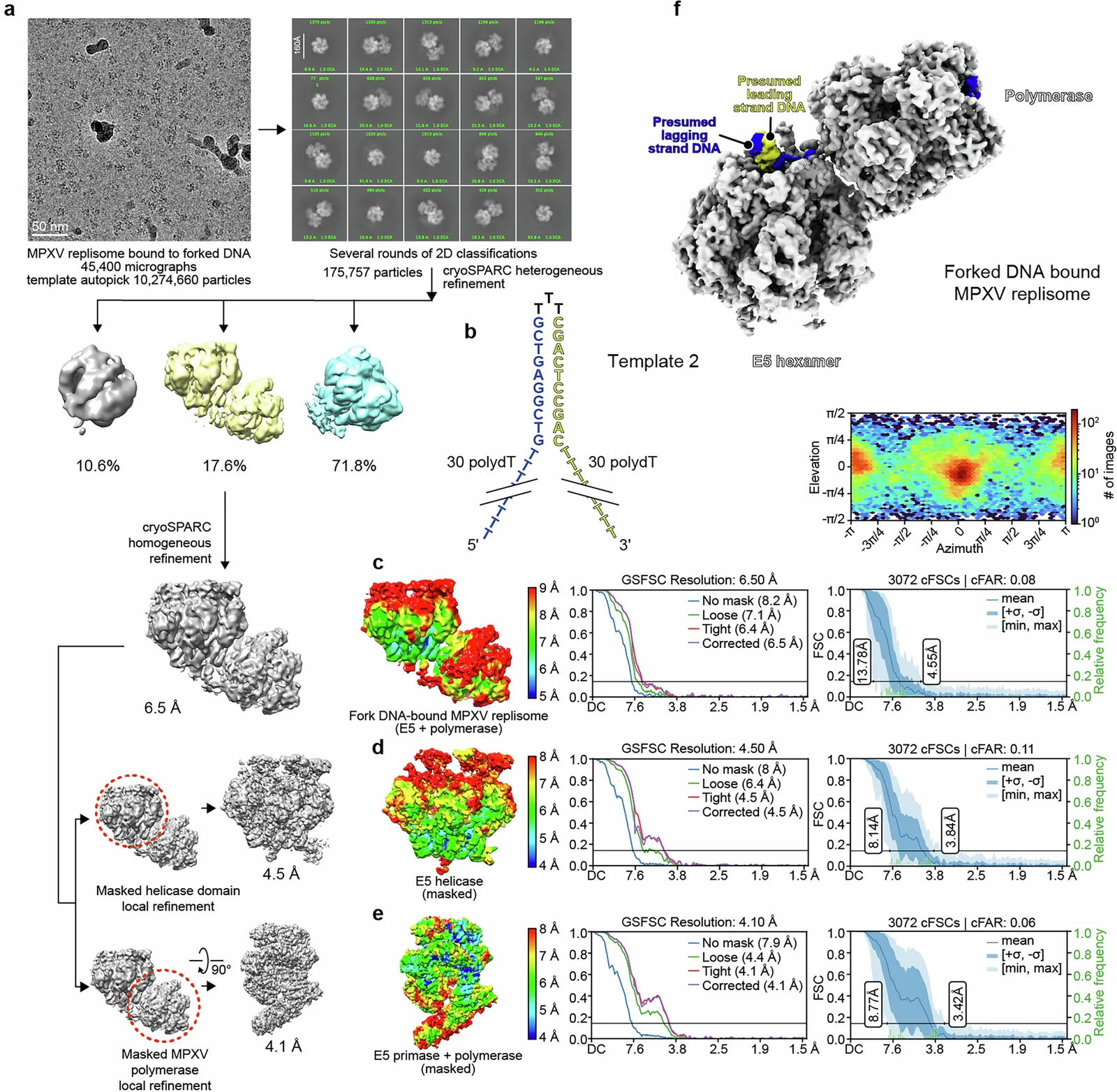
Cryo-EM reconstruction of MPXV replisome bound to forked DNA. **a**, Workflow used for cryo-EM data processing of forked DNA-bound MPXV replisome. **b**, Schematic diagram of the forked hairpin DNA (template 2) used to determine the structure of the forked DNA-bound MPXV replisome. **c**–**e**, Local resolution estimation, Fourier shell correlation (FSC) curves, 3D-FSC curves, and particle angular distributions of the cryo-EM reconstructions of the forked DNA-bound MPXV replisome determined at an overall resolution of 6.5 Å (**c**), with a masked helicase map at 4.5 Å resolution (**d**), and masked map of the polymerase–primase region at 4.1 Å resolution (**e**). **f**, Composite cryo-EM map of the forked DNA-bound MPXV replisome, post-processed with DeepEMhancer^46^.

In the ssDNA-bound replisome structure, the ssDNA segment bridging E5 and the polymerase is solvent exposed. We hypothesized that this region would accommodate double-stranded DNA (dsDNA) as part of a replication fork. To test this hypothesis, we determined the cryo-EM structure of a complex including E5 and polymerase assembled on a forked DNA substrate (template 2) (Extended Data Fig. 5a,b and Supplementary Tables 2 and 4). The global resolution of that map was 6.5 Å, and we obtained a 4.5 Å map focusing on the hexameric E5 helicase and a 4.1 Å map focusing on the polymerase and two associated E5 primase domains (Fig. 1f and Extended Data Fig. 5c–e). We observed clear density for the duplex region of the forked DNA at the same position as the bridging ssDNA density in the ssDNA-bound complex (Extended Data Figs. 2b and 5f).

## Contacts mediating complex assembly

As the ssDNA-bound replisome structure was higher resolution than the forked-DNA-bound structure, we focused our analysis on the former. The E5_A_ RRM interacts with the F8 thumb through hydrophobic and polar contacts that centre on F8 residue Trp940 (Extended Data Fig. 3e). The E5_A_ ZBM bridges the polymerase with E5 by interacting with the F8 thumb and the E5_A_ helicase domain through polar and hydrophobic contacts (Extended Data Fig. 3f). E5_F_ RRM interactions with A22 involve hydrophobic contacts that centre on A22 residues Phe217 and Phe263 (Extended Data Fig. 3g). E5 interaction with A22 is supported by a previous study that identified direct interactions between vaccinia virus (VACV) E5 and A20 (the same gene product as A22 in MPXV) in yeast two-hybrid experiments^21^.

## MPXV polymerase activates E5 helicase

**Fig. 2:**
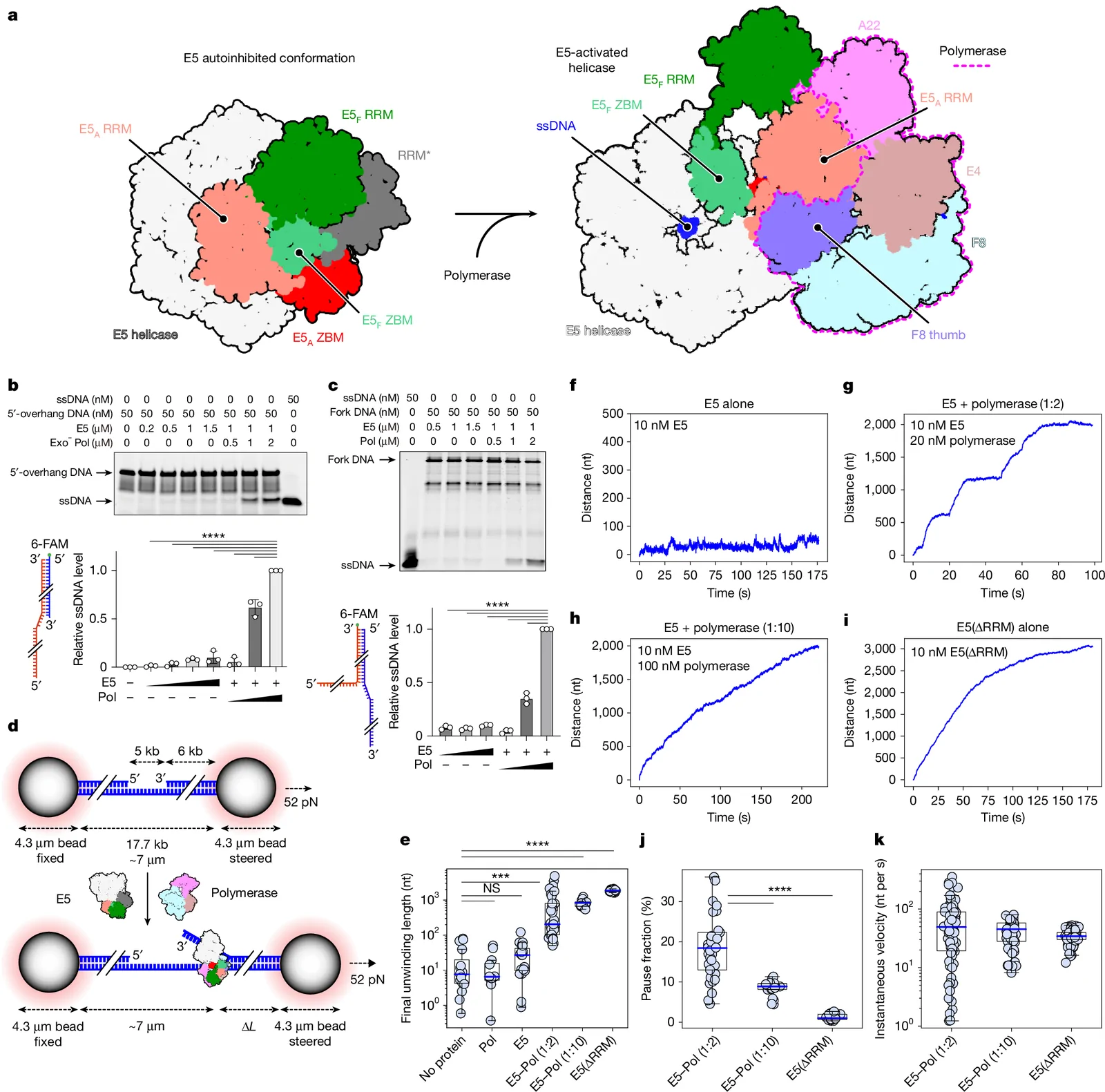
Polymerase binding activates the E5 helicase. **a**, Diagrams of E5 alone (left; PDB: 8HWA)^3^ or in the ssDNA-bound replisome (right). RRM*, unknown RRM. **b**,**c**, Helicase assay with E5 alone or with polymerase using 5′-overhang-containing substrate (**b**) or forked DNA substrate (**c**). The ssDNA control is the unannealed primer. DNA substrates and quantification of the ssDNA bands are shown. Pol, polymerase. **d**, Optical tweezer experiment design (Methods). Δ*L*, increasing distance. **e**, Unwinding lengths at 60 s of a control condition (no protein, *n* = 16), polymerase alone (*n* = 12), E5 alone (*n* = 19), E5 with polymerase 1:2 ratio (*n* = 33), E5 with polymerase 1:10 ratio (*n* = 19) or E5(ΔRRM) (*n* = 25); *n* values represent the number of independent traces. **f**–**i**, Representative traces of unwinding activity with E5 alone (**f**), E5 with polymerase (containing exonuclease-deficient F8) at different ratios (**g**,**h**) or E5(ΔRRM) (**i**) at the indicated concentrations. **j**, Pause fractions of E5 with polymerase at a 1:2 ratio (*n* = 33) or 1:10 ratio (*n* = 19), or E5(ΔRRM) (*n* = 25); *n* values represent the number of independent traces. **k**, Instantaneous velocities of E5 with polymerase at a 1:2 ratio (*n* = 75) or 1:10 ratio (*n* = 53), or E5(ΔRRM) (*n* = 92); *n* values represent independent unwinding events derived from traces. For **b** and **c**, data are mean ± s.d. Each experiment was performed three times (*n* = 3), and representative gels are shown. Statistical analysis was performed using one-way analysis of variance (ANOVA) with Tukey’s multiple-comparison test; ****P < 0.0001. In **e**, **j** and **k**, the box plots show the median (blue line) and the interquartile range (IQR; 25th to 75th percentile; box limits), and the whiskers extend to the minimum and maximum values within 1.5× the IQR. Individual points represent raw data. Comparisons were performed using Welch’s *t*-tests; all statistical tests are two-tailed comparisons; NS, not significant; ***P = 0.000499. nt, nucleotides. Source data

**Extended Data Fig. 6:**
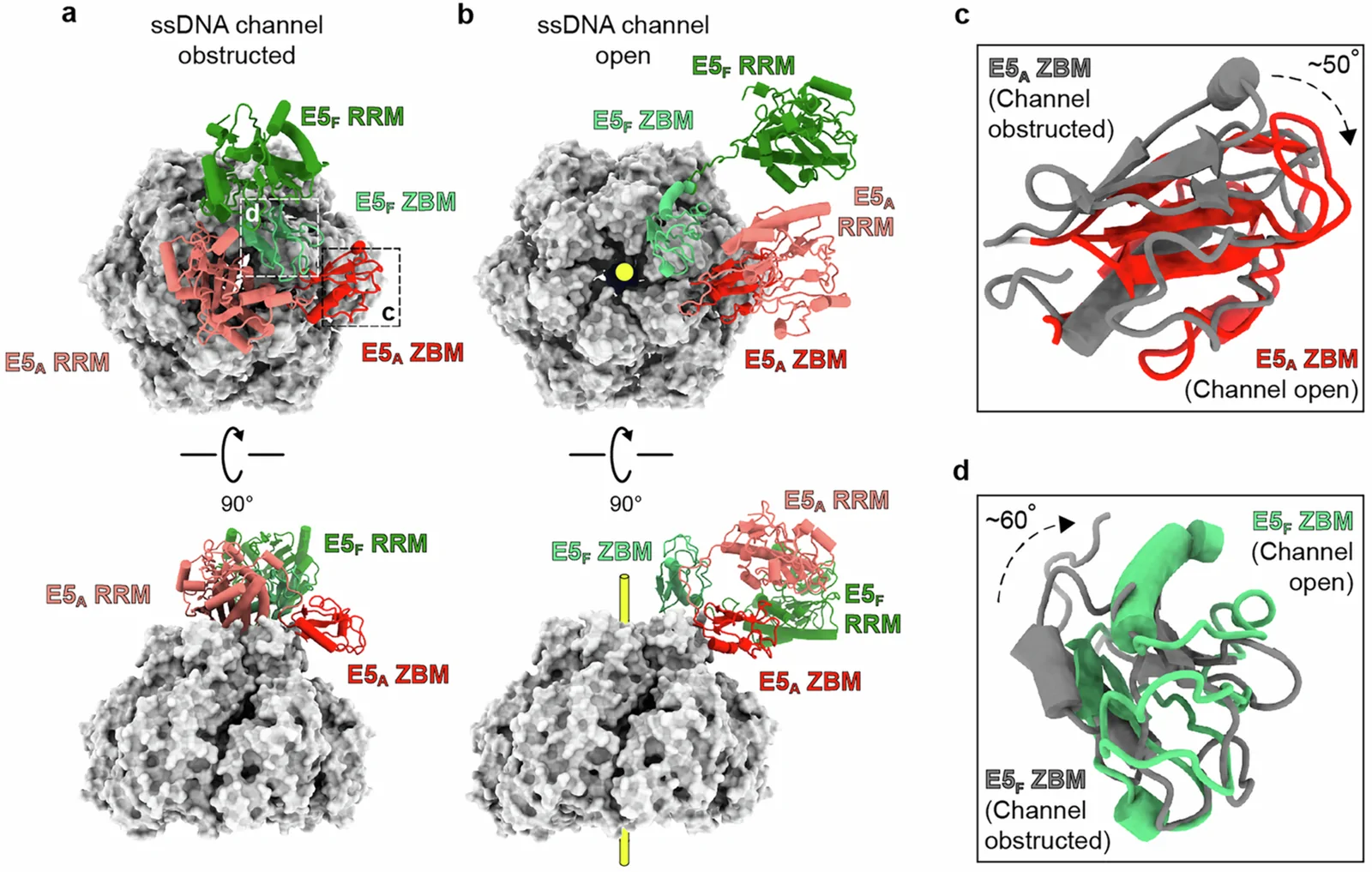
Comparison of E5 alone and E5 in the MPXV replisome. **a**, **b**, Two different views of E5 helicase hexamer and primase domains of E5_A_ and E5_F_. In E5 without polymerase, the ssDNA channel is obstructed (**a**), but when E5 associates with polymerase, the ssDNA channel is open (**b**). The E5 helicase is shown in surface representation. The RRM and ZBM of E5_A_ and E5_F_ are shown as ribbon diagrams. The location of the ssDNA channel is shown as a yellow cylinder. The E5_A_ RRM centre of mass is displaced by 58 Å to interact with the F8 thumb. The E5_F_ RRM centre of mass is displaced by 54 Å to interact with A22. **c**,**d**, Conformational changes occurring in the E5_A_ ZBM (**c**) or E5_F_ ZBM (**d**) when E5 is alone or interacting with polymerase.

Compared with the conformation of E5 in isolation^3^, the E5 primase domains undergo large-scale conformational changes when they bind to the polymerase (Fig. 2a and Extended Data Fig. 6). These conformational changes in the E5 primase domains result in exposure of the helicase ssDNA entry channel (Extended Data Fig. 6a,b). The E5_A_ and E5_F_ ZBMs mostly stay in a similar location, slightly rotating 50° and 60°, respectively, to allow repositioning of the RRMs (Extended Data Fig. 6c,d). While a previous structure of E5 in isolation showed a third RRM^3^, this third RRM is not visualized when E5 interacts with the polymerase.

**Extended Data Fig. 7:**
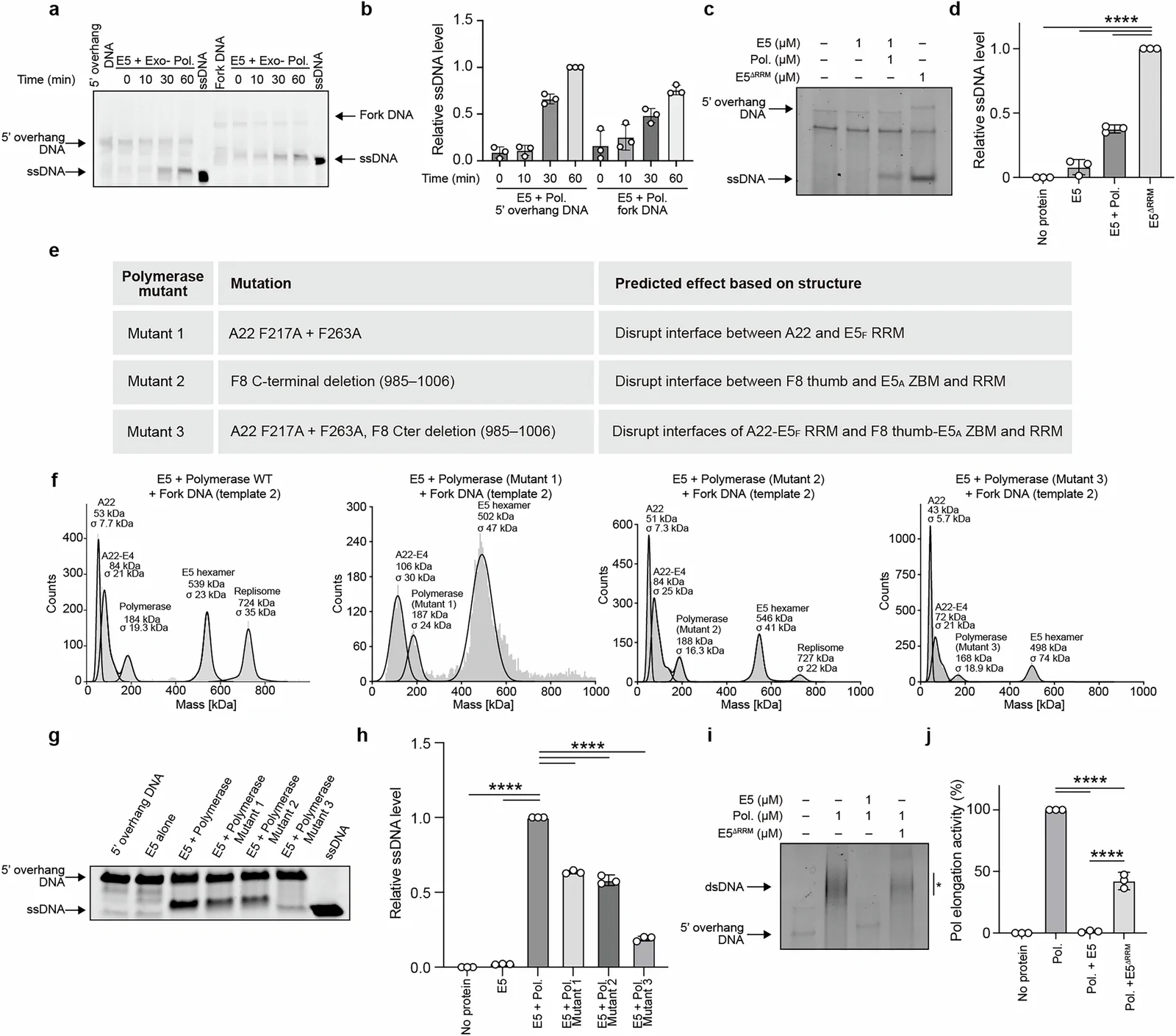
Impact of E5–polymerase interface mutations on helicase activity and DNA polymerase elongation activity assays. **a**,**b**, Time-course helicase activity assay performed with MPXV E5 co-incubated with polymerase using DNA substrate with a 5′ overhang or a fork DNA substrate (**a**). Quantification of the ssDNA, with mean ± s.d., is shown (**b**). This experiment was performed three times (*n* = 3), and a representative gel is shown. **c**,**d**, Helicase activity assay performed with MPXV E5 alone or co-incubated with polymerase or E5^ΔRRM^ alone using DNA substrate with a 5′ overhang (**c**). Quantification of the ssDNA, with mean ± s.d., is shown (**d**). This experiment was performed three times (*n* = 3), and a representative gel is shown. One-way ANOVA with Tukey’s multiple comparison test (****P < 0.0001). **e**, Summary of the mutations designed to disrupt specific interfaces between the polymerase and E5. See also Extended Data Fig. 3e–g. **f**, Mass photometry results of wild type MPXV replisome and MPXV replisome with mutant 1, 2 or 3. This experiment was performed twice (*n* = 2), with representative data shown. WT, wild type. **g**,**h**, Helicase activity assay performed with MPXV E5 alone or co-incubated with wild-type or mutant polymerases using DNA substrate with a 5′ overhang (**g**). Quantification of the ssDNA band, with mean ± s.d., is shown (**h**). This experiment was performed three times (*n* = 3), and a representative gel is shown. One-way ANOVA with Tukey’s multiple comparison test (****P < 0.0001). **i**,**j**, Polymerase DNA elongation assay performed with polymerase alone or co-incubated with wild type E5 or E5^ΔRRM^ using a DNA substrate containing a 5′ overhang (**i**). The region of the gel that was quantified is indicated with an asterisk. Quantification is shown (**j**). This experiment was performed three times (*n* = 3), and a representative gel is shown. One-way ANOVA with Tukey’s multiple comparison test (****P < 0.0001). Source data

As the conformational change noted above uncovers the E5 ssDNA entry channel, we hypothesized that polymerase binding activates E5. To test this, we used biochemical assays to examine E5 helicase activity in the presence of polymerase. Previous studies on E5 helicase activity suggest that it can have higher activity on forked DNA substrates than on substrates with either 3′ or 5′ overhangs^4^. We used two substrates for activity assays. The first contains a 5′-labelled 6-carboxyfluorescein (6-FAM) primer annealed to a template and has a 5′ overhang, organized like the template that was used for structural analysis of the ssDNA-bound replisome (Fig. 2b). The second is a dsDNA fork comprising a 3′-labelled 6-FAM primer annealed to a template, organized like the DNA substrate that was used for structural analysis of the forked DNA-bound replisome (Fig. 2c). To avoid MPXV F8 exonuclease-mediated cleavage of the 3′ end present in the 5′ overhang DNA substrate, we used a polymerase containing an exonuclease-dead F8 subunit (with the F8 D166A and E168A substitutions)^5^. In both assays, we observed no ssDNA in samples containing only E5. However, addition of polymerase increased the amount of ssDNA. In assays with the DNA substrate containing a 5′ overhang or a forked DNA substrate, we observed similar unwinding activity of E5 with polymerase under our experimental conditions (Extended Data Fig. 7a,b).

When full-length E5 is in isolation, the ZBM and RRM domains obstruct the ssDNA channel, causing autoinhibition of helicase activity^3,4^. Removal of the N-terminal region of E5 containing the RRM domains has previously been shown to relieve E5 autoinhibition, therefore yielding an active helicase^3,4^. We purified an E5 construct in which the RRM is deleted (E5(ΔRRM), containing residues 238–785)^3^. When tested in helicase activity assays, we found that E5(ΔRRM) is more active than full-length E5 in the presence of polymerase (Extended Data Fig. 7c,d).

## Single-molecule analysis of E5 activation

To directly observe unwinding activity on individual DNAs, we used a dual-optical-tweezer system to tether a 17.7 kb dsDNA–ssDNA hybrid molecule between two streptavidin-coated polystyrene beads^22,23^ (Fig. 2d). We conducted unwinding experiments in a constant-force mode with a high-frequency-feedback system, adjusting the position of a bead to maintain the desired force (52 pN). Unwinding activity would increase the distance between the beads due to conversion of dsDNA to ssDNA^24^ (Fig. 2d). When held at this force with no proteins present, we observed no evidence of unwinding or spontaneous DNA breakage of the tethered DNA substrate (*n* = 16 traces) (Fig. 2e and Supplementary Fig. 1a).

In experiments containing MPXV E5 alone, we observed only minor helicase activity (*n* = 19) (Fig. 2e,f and Supplementary Fig. 1b). We used exonuclease-deficient polymerase in assays to avoid potential cleavage of the DNA 3′ end. As expected, MPXV polymerase alone had no measurable activity (*n* = 12) (Fig. 2e and Supplementary Fig. 1c). However, with both E5 and polymerase present, we observed a markedly greater extent of DNA unwinding compared with E5 alone (*n* = 33) (Fig. 2e,g and Supplementary Fig. 1d).

In some of the traces, we also observed pauses in helicase unwinding activity. We hypothesized that pauses may be instances of the polymerase dissociating from E5. If so, increasing the polymerase concentration would diminish the number of pauses. Indeed, increasing the concentration tenfold with respect to E5 substantially decreased pauses in unwinding activity, from a pause fraction of 18.4% with E5 and polymerase at a 1:2 ratio (*n* = 33) to 8.9% with E5 and polymerase at a 1:10 ratio (*n* = 19) (Fig. 2e,h,j and Supplementary Figs. 1e and 2a). We found that the E5(ΔRRM) protein in these assays had a low-pause fraction (1%, *n* = 25) (Fig. 2e,i,j and Supplementary Fig. 2b). Correcting for the pauses in the measurement of unwinding activity revealed that the pause-free velocity remains relatively consistent across all conditions: 49 ± 9 bp s^−1^ (1:2 E5-to-polymerase ratio), 45 ± 3 bp s^−^^1^ (1:10 ratio) and 34 ± 1 bp s^−^^1^ for the E5(ΔRRM) mutant (Fig. 2k). These findings suggest that the large-scale conformational changes that occur when E5 binds to the polymerase regulate E5 helicase activity.

## Polymerase enhances E5 primase activity

**Fig. 3:**
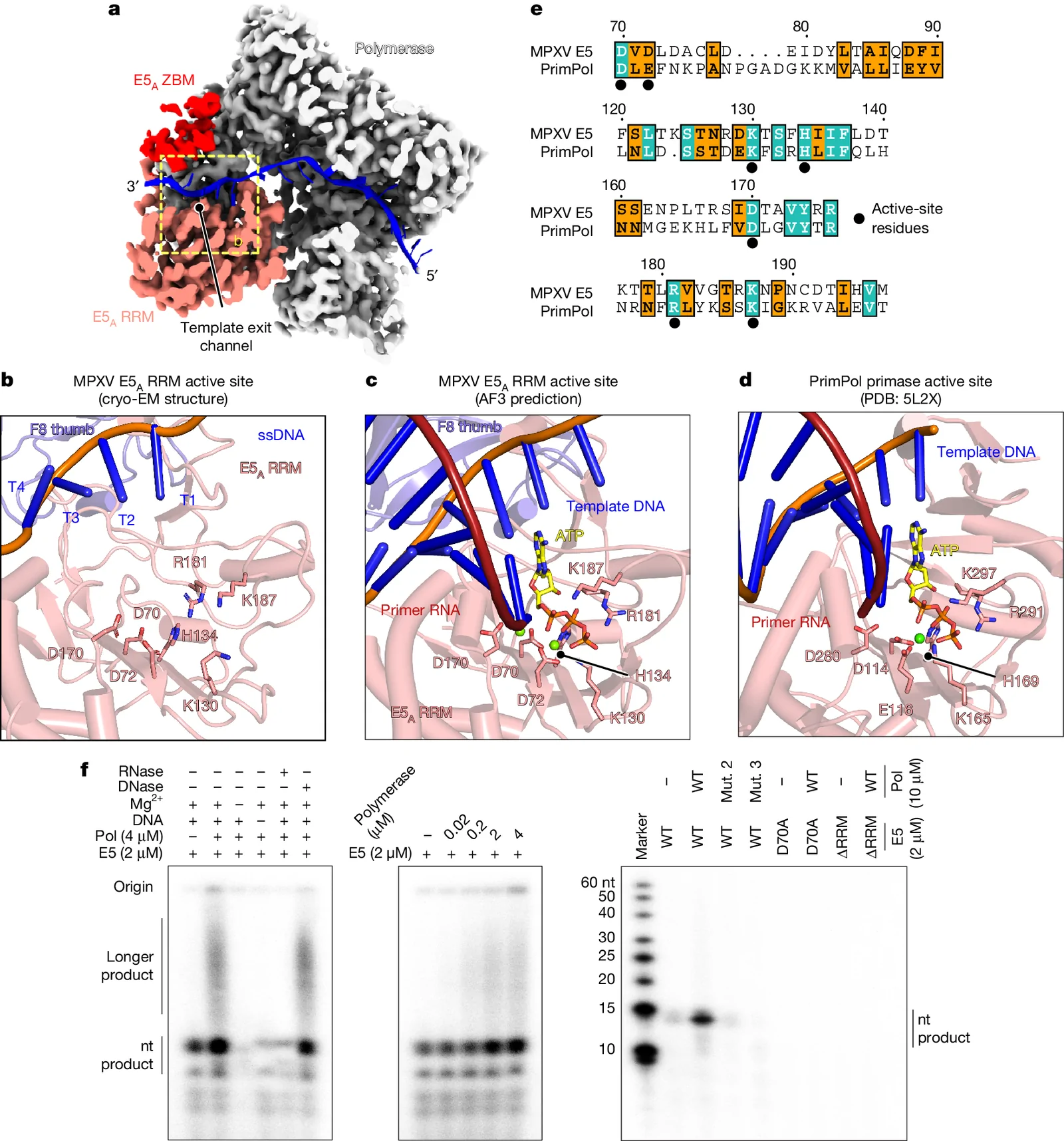
MPXV polymerase binding enhances E5 primase activity. **a**, Cut-off view of the cryo-EM density of MPXV polymerase and E5_A_ primase (ZBM and RRM) shown as a surface representation from the map of the ssDNA-bound replisome (E5 primase and polymerase masked, 3.5 Å) post-processed with DeepEMhancer^46^. The bound ssDNA is shown as a ribbon diagram. **b**, E5_A_ RRM active site in the ssDNA-bound MPXV replisome. Active-site residues that are expected to interact with active site metals and incoming nucleotide are shown as sticks. **c**, AF3 (ref. ^25^) prediction of the E5 RRM active site in the presence of F8 thumb domain, template DNA, primer RNA, ATP and magnesium ions (green spheres). Predicted local distance difference test scores are shown in Extended Data Fig. 8c. **d**, Human PrimPol active site (PDB: 5L2X)^28^. **e**, Sequence alignments of the active site of MPXV E5 (GenBank: XNX20584.1) and human PrimPol (GenBank: NP_001332824). Conserved active-site residues are indicated. **f**, MPXV E5 primase RNA synthesis assay performed in the presence or absence of wild-type or mutant polymerases with M13 ssDNA and α-^32^P-labelled CTP; radioactive products were separated on a urea–PAGE gel. Each experiment was performed twice (*n* = 2); representative gels are shown. mut, mutant; WT, wild type.

**Extended Data Fig. 8:**
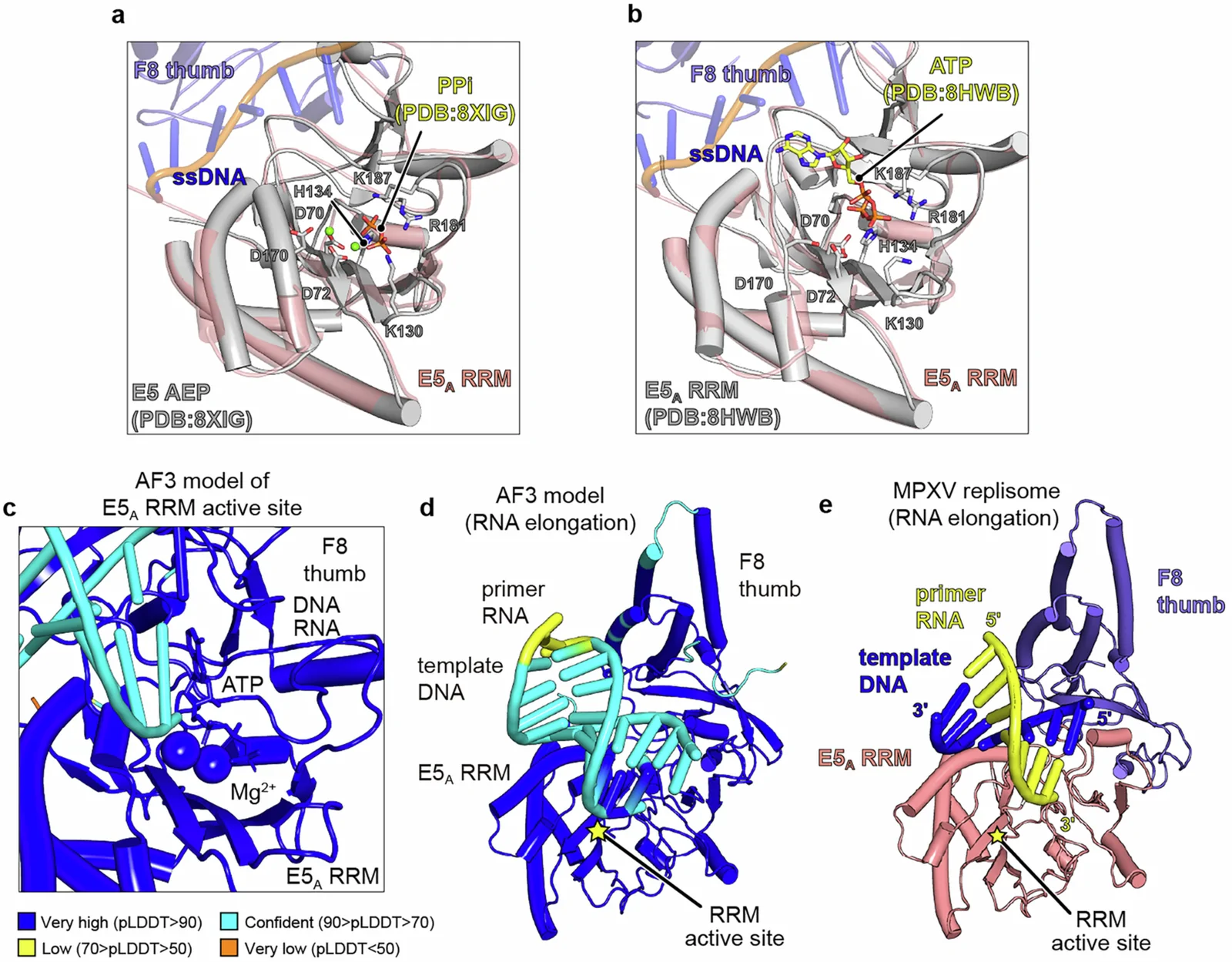
RRM active site configuration. **a**, Structural superposition of the E5_A_ RRM active site in the ssDNA-bound MPXV replisome and the crystal structure of the E5 RRM domain (PDB: 8XIG)^4^ bound to pyrophosphate (PP_i_) and two magnesium ions (green spheres). **b**, Structural superposition of the E5_A_ RRM active site in the ssDNA-bound MPXV replisome and ATP-bound E5_A_ RRM; the ATP-bound E5_A_ RRM is from a prior cryo-EM structure of E5 in isolation (PDB: 8HWB)^3^. **c**, Predicted local distance difference test (pLDDT) scores of the E5_A_ RRM active site of the AF3 model. The pLDDT score scale is provided. **d**, pLDDT of an AF3 model of RNA-DNA bound MPXV polymerase–primase core subcomplex including RNA-DNA hybrid bound to the F8 thumb and E5_A_ RRM. **e**, Active site configuration of an MPXV polymerase–primase core complex bound to RNA-DNA hybrid (RNA elongation state) from the cryo-EM structure.

The E5_A_ ZBM and RRM interact with an ssDNA segment in the template exit channel (Fig. 3a). E5_A_ RRM interactions with ssDNA position the RRM putative active site to face ssDNA bases in a state before primase-mediated nucleotide addition (Fig. 3b). Indeed, residues forming the RRM active site have been shown to bind to pyrophosphate and metals in the crystal structure of the MPXV E5 RRM domain^4^ and were bound to ATP in a previous E5 structure^3^. We superposed the crystal structure of the isolated E5 RRM domain (PDB: 8XIG)^4^ and ATP-bound E5 RRM (PDB: 8HWB)^3^ with the RRM conformation observed in the ssDNA-bound replisome cryo-EM structure to reveal how additional metals and nucleotides may be recognized (Extended Data Fig. 8a,b).

We used AlphaFold 3 (AF3)^25^ to predict the RRM active-site recognition of a DNA template, an RNA primer, incoming ATP and two active-site metals (Fig. 3c and Extended Data Fig. 8c). In the nucleotide-bound active site, E5 primase active-site residues Lys130, His134, Arg181 and Lys187 are predicted to interact with the phosphate groups of the incoming nucleotide, while Asp70 and Asp72 would coordinate an active site metal. E5 residue Asp170 would be positioned to interact with a second metal during nucleotide incorporation^26^ (Fig. 3c).

Examination of E5 primase domain sequence alignments revealed that basic and acidic residues forming the putative active site are highly conserved among poxviruses (Extended Data Fig. 4). The primase domain of E5 belongs to the archaeoeukaryotic primase family^27^. The active-site configuration is like that of human PrimPol, another archaeoeukaryotic primase family enzyme, for which a 2.2 Å X-ray crystal structure is available (PDB: 5L2X)^28^, and all active-site residues are conserved (Fig. 3d,e). The RRM active site configuration is supported by previous studies showing that E5 RRM acidic residues Asp70, Asp72, Asp170 and basic residues His134, Arg181 and Lys187 are required for primase activity in vitro^4^ and that VACV D5 (analogous to MPXV E5) residues Asp70, Asp72 and Asp170 are required for DNA replication^13^ (Supplementary Table 5).

To examine the effect of complex assembly on RNA primer synthesis, we performed E5 primase activity assays in the presence of [α-^32^P]cytidine triphosphate (CTP) and other unlabelled ribonucleotide triphosphates (rNTPs) with or without addition of polymerase (Fig. 3f). E5 alone was active, as radiolabelled RNA products were resistant to DNase treatment but susceptible to RNase treatment. The major RNA product synthesized by E5 was smaller than 15 nucleotides, consistent with product sizes reported previously for VACV D5 (ref. ^13^). E5 containing the RRM D70A mutation that would disrupt metal binding in the active site, or E5(ΔRRM), which lacks a primase domain, yielded no RNA synthesis. Addition of polymerase enhanced E5 RNA synthesis activity and apparent product size in a concentration-dependent manner. These findings suggest that MPXV polymerase, through interactions with the E5 primase domains, has a regulatory role in RNA synthesis.

## Functional assessment of replisome assembly

We next studied recombinant polymerases (F8–E4–A22) with alterations expected to disrupt polymerase interactions with E5. We introduced the F217A and F263A substitutions in A22 to impair hydrophobic interactions with the E5_F_ RRM (Extended Data Figs. 3g and 7e). We also introduced a deletion in the F8 thumb C-terminal residues 985–1006 to impair interactions with the E5_A_ ZBM and RRM (Extended Data Figs. 3e,f and 7e). We purified polymerases containing mutant A22 with wild-type F8 and E4 subunits (mutant 1), mutant F8 with wild-type A22 and E4 subunits (mutant 2), or mutant A22 and mutant F8 with wild-type E4 subunit (mutant 3). Compared with wild-type polymerase, MP experiments showed that mutants 1, 2 and 3, when incubated with forked DNA (template 2) and E5, were impaired at forming complexes of the expected size for replisomes (756 kDa) (Extended Data Fig. 7f).

We tested polymerase mutants 1, 2 and 3 in helicase activity assays designed to monitor unwinding of 5′-overhang DNA substrate and found that they were impaired at enhancing E5 helicase activity (Extended Data Fig. 7g,h). In a E5 primase activity assay, the two mutant polymerases we tested (2 and 3) did not enhance primase activity (Fig. 3f). Our results suggest the mutations of polymerase–E5 interface residues determined through cryo-EM structures impair replisome assembly and prevent polymerase-mediated E5 helicase–primase activation.

## Mechanism of RNA primer elongation

**Extended Data Fig. 9:**
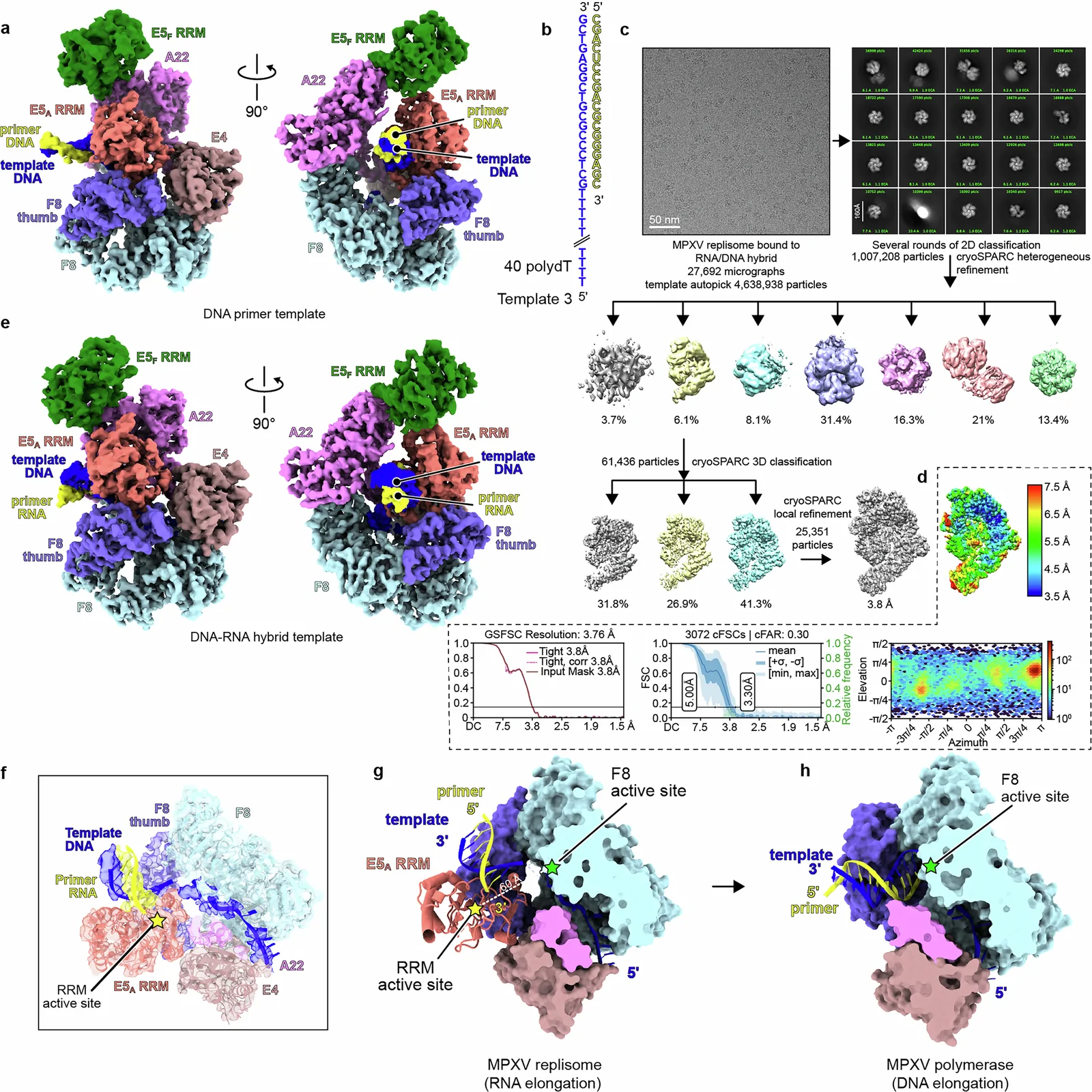
Cryo-EM reconstruction of MPXV polymerase–primase core complex bound to RNA-DNA hybrid. **a**, Two views of the 4.0 Å cryo-EM map of the polymerase–primase core complex bound to dsDNA (see also Extended Data Fig. 1h). **b**, Schematic diagram of RNA-DNA substrate (template 3) used for cryo-EM structure determination. **c**, Workflow used for cryo-EM data processing of the polymerase–primase core complex bound to RNA-DNA hybrid. **d**, Local resolution estimation, Fourier shell correlation (FSC) curves, 3D-FSC curves and particle angular distributions of the cryo-EM reconstruction of the polymerase–primase core bound to RNA-DNA hybrid at 3.8 Å resolution. **e**, Two views of the 3.8 Å cryo-EM map of polymerase–primase core complex bound to RNA-DNA hybrid. **f**, Cryo-EM density (3.8 Å) of E5_A_ RRM active site of polymerase–primase core complex bound to RNA-DNA hybrid shown as surface. The RRM active site is indicated. **g**,**h**, Comparison between the polymerase–primase core complex bound to RNA-DNA hybrid and the dsDNA-bound polymerase in a DNA elongation state (PDB: 8HG1)^5^. The E5_A_ RRM and nucleic acids are shown as ribbon diagrams. The locations of the RRM and F8 active sites are indicated. The distance between the two active sites is labelled with a dashed line. Maps shown in **a**, **e**, **d** and **f** were post-processed with DeepEMhancer^46^.

Examining volumes from the first step of 3D classification revealed a subclass of particles resembling the MPXV polymerase but with density for two E5 RRMs: one interacting with the F8 thumb (E5_A_ RRM) and another interacting with A22 (E5_F_ RRM) (Extended Data Figs. 1h and 9a). The entire hexameric E5 helicase and E5_A_ and E5_F_ ZBMs could not be visualized, perhaps because they are flexible with respect to the rest of the complex. However, we obtained a 4.0 Å map for a complex denoted here as a polymerase–primase core complex (Supplementary Table 3). This map had density for ssDNA going through the ssDNA-binding channel within the polymerase (lined by E4 and F8) and for dsDNA interacting with the E5_A_ RRM. The dsDNA segment probably represented the hairpin portion of the DNA oligo (template 1), with the captured state relevant to RNA elongation.

**Fig. 4:**
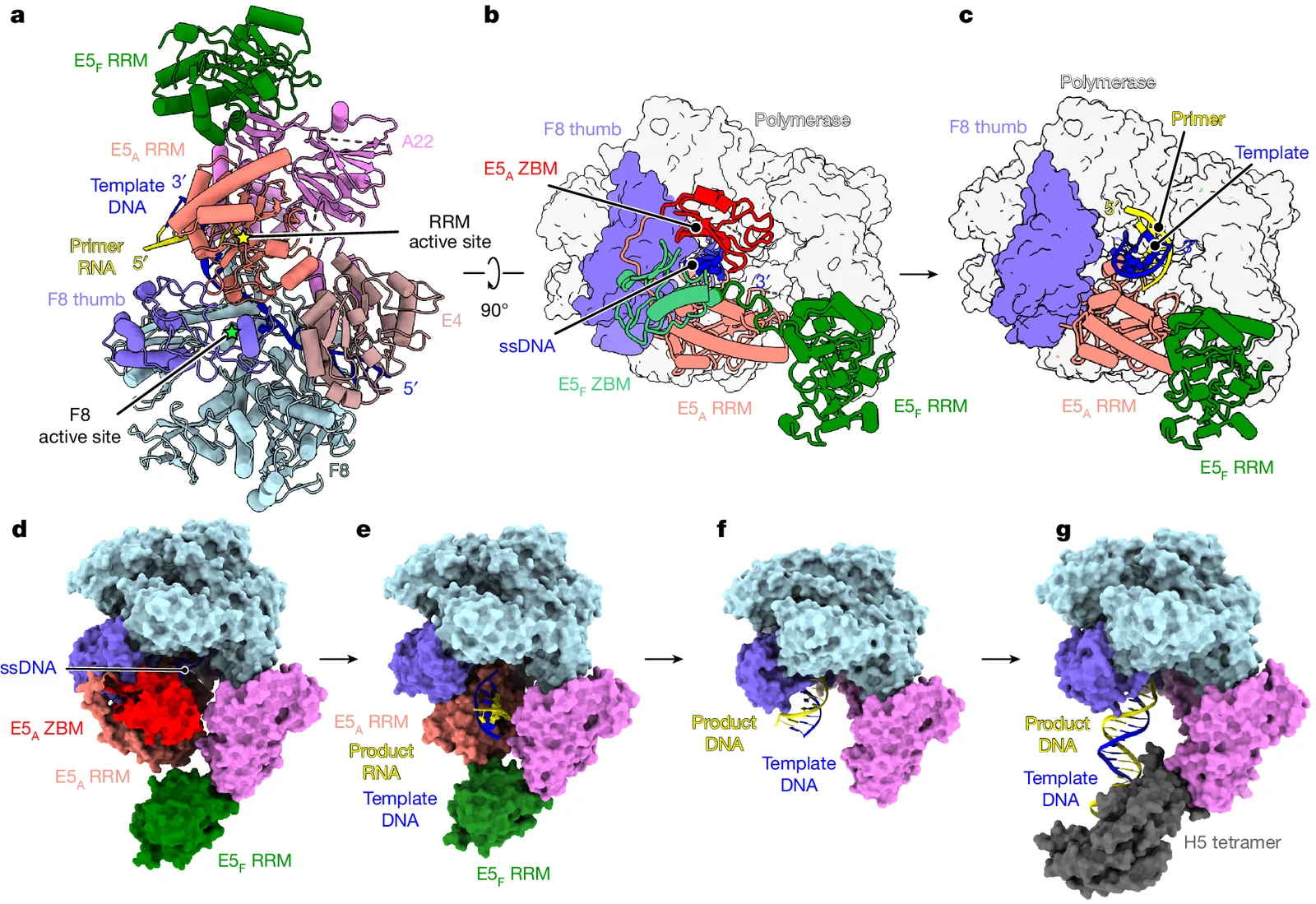
Structure of an MPXV polymerase–primase core complex and RNA-to-DNA elongation transitions. **a**, Ribbon diagram of an MPXV polymerase–primase core complex bound to an RNA–DNA hybrid template from maps obtained at a resolution of 3.8 Å (Extended Data Fig. 9e). The locations of the RRM and F8 active sites are indicated. **b**,**c**, Comparison of MPXV polymerase–primase core complex in the structure of the ssDNA-bound replisome (**b**) or bound to RNA–DNA hybrid (**c**). The polymerase is shown as a surface representation. E5 primase domains and nucleic acids are shown as ribbon diagrams. **d**–**g**, Comparison of the structures of an MPXV polymerase–primase core complex bound to ssDNA (RNA initiation) (**d**); polymerase–primase core complex bound to RNA–DNA hybrid (RNA elongation) (**e**); polymerase bound to dsDNA (DNA elongation state) (PDB: 8HG1)^5^ (**f**); and polymerase bound to dsDNA and to the processivity factor, which is the H5 tetramer (PDB: 8WPP)^7 ^(**g**). Proteins and subdomains are shown as a surface representation, and nucleic acids are shown as ribbon diagrams.

We next co-incubated MPXV polymerase and E5 with an RNA primer–DNA hybrid template (template 3) and used cryo-EM to obtain a 3.8 Å map of a polymerase–primase core complex (Fig. 4a, Extended Data Fig. 9b–e and Supplementary Table 6). Maps for the polymerase–primase core complex bound to DNA (4.0 Å) revealed an identical organization to maps of the same complex bound to the RNA–DNA hybrid template (3.8 Å), with a correlation coefficient of 0.68. We used AF3 (ref. ^25^) to predict interactions in a polymerase–primase subcomplex including the F8 thumb, the E5 RRM and an RNA–DNA hybrid (Extended Data Fig. 8d). Comparison between the cryo-EM structure and the AF3 model revealed close similarity, with an all-atom root mean square deviation of only 0.85 Å. The binding mode between the F8 thumb domain and the E5 RRM is nearly identical, and the RNA–DNA hybrid in the cryo-EM structure aligns closely with the RNA–DNA hybrid orientation predicted by the AF3 model (Extended Data Fig. 8d,e).

Importantly, in the polymerase–primase core complex, potentially consistent with an RNA elongation state, the 3′ end of the product RNA strand is pointed towards the E5_A_ RRM active site, rather than the F8 palm active site that is usually responsible for DNA elongation (Fig. 4a and Extended Data Fig. 9f).

In the polymerase–primase core complex maps, we observed two of the E5 primase domains, but not the hexameric helicase domains. Comparing the ssDNA-bound replisome to the RNA–DNA-hybrid bound polymerase–primase core complex suggested that E5_A_ ZBM interactions with the F8 thumb are weakened during RNA elongation (Fig. 4b,c). As the E5_A_ ZBM bridges the E5 helicase with the F8 thumb (Extended Data Fig. 3f), disruption of the E5_A_ ZBM–F8 thumb contacts as RNA is synthesized may render the hexameric helicase domains more flexible with respect to the polymerase–primase core. Moreover, weakening the E5_A_ ZBM contacts with the F8 thumb may create more space to accommodate RNA products (Fig. 4b,c).

## RNA versus DNA elongation transitions

Comparing the structure of the polymerase–primase core complex bound to RNA–DNA hybrid with a previously reported dsDNA-bound MPXV polymerase structure (PDB: 8HG1)^5^ shows that the orientation of the duplex shifts by roughly 60° during DNA elongation (Extended Data Fig. 9g,h). This shift allows the 3′ end of the RNA primer to be displaced by around 60 Å as it transitions from the primase RRM active site to the F8 catalytic centre for nucleotide addition during DNA elongation. The structures thus suggest that E5 primase domain binding to dsDNA would impair polymerase DNA elongation. Indeed, addition of E5 inhibited DNA polymerase activity in an elongation assay (Extended Data Fig. 7i,j). The E5(ΔRRM) construct, unable to interact with the polymerase, only partially reduced DNA elongation, probably through competition for DNA substrate.

The poxvirus protein H5 associates with the DNA polymerase holoenzyme and is required for DNA replication^29^. H5, as a tetramer, binds to the dsDNA downstream of F8 to make additional contacts with dsDNA in a position similar to processivity factors (for example, proliferating cell nuclear antigen (PCNA))^30–33^. Comparing the MPXV replisome structures reported here to the dsDNA-bound elongation state of the MPXV DNA polymerase without (PDB: 8HG1)^5^ or with the H5 tetramer (PDB: 8WPP)^7^ revealed that the E5_F_ RRM and H5 compete with the same surface on A22 (Fig. 4d–g). Thus, recruitment of the H5 tetramer may have a regulatory role in facilitating the transition from RNA primer synthesis to DNA elongation, promoting processive DNA replication.

## Discussion

Here we report that association of the heterotrimeric MPXV DNA polymerase with the E5 helicase–primase regulates the activity of the helicase and primase. Interactions between the DNA polymerase and the E5 primase domains probably represent a critical regulatory mechanism in the viral DNA replication process.

**Fig. 5:**
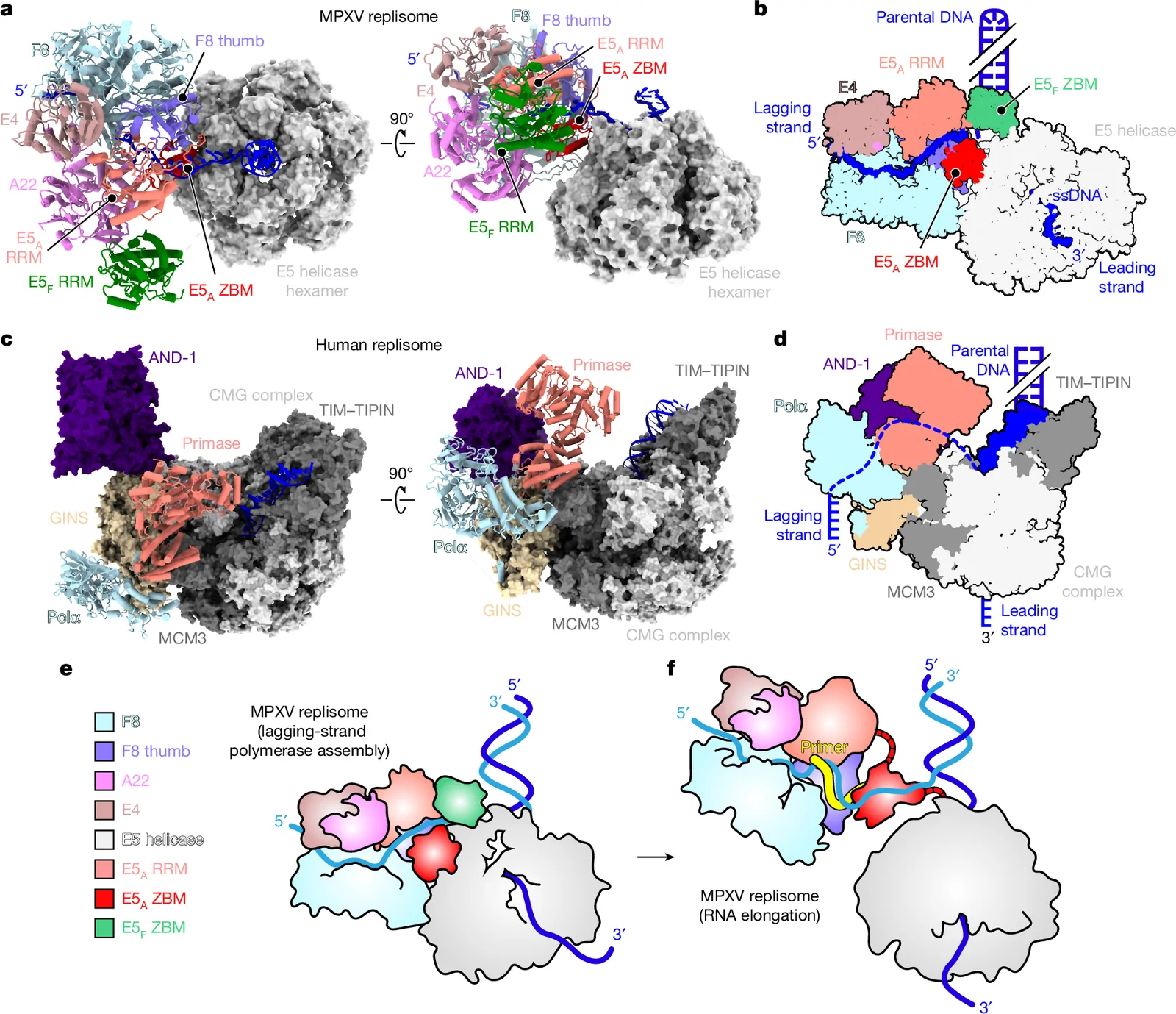
Comparison of the MPXV and human replisomes and mechanism of replication initiation. **a**,**b**, Organization of the MPXV replisome bound to forked DNA substrate shown as ribbon and surface representations (**a**) and as a schematic (**b**). **c**,**d**, Organization of the human replisome (PDB: 8B9D)^35^ bound to a forked DNA substrate shown as ribbon and surface representations (**c**) and as a schematic (**d**). The human replisome contains the CMG complex (Cdc45–Mcm2–7–GINS), Polα–primase, TIMELESS (TIM)–TIPIN, AND-1 and CLASPIN^35^. The Mcm2–7 helicase, part of the CMG complex, is an SF6 helicase that unwinds dsDNA in the 3′–5′ direction on the leading strand, the same direction as poxvirus E5. Polα–primase is found on the lagging strand, where it interacts with Mcm3, GINS and AND-1. The dashed blue line in **d** shows the expected path of the ssDNA. **e**,**f**, Working model of MPXV E5 helicase–primase and polymerase loading (**e**) and DNA unwinding followed by RNA primer addition (**f**). The E5_F_ RRM, which interacts with A22, is not shown as it would be in the front plane of the page.

One structure of a viral replisome (bacteriophage T7)^34^ and two structures of eukaryotic (*Saccharomyces cerevisiae* and human) replisomes with polymerase–primases bound to lagging strands are available^35^. Although the human replisome is more complex, the positioning of the helicase, primase and polymerase at the replication fork in MPXV resembles that of the human replisome, highlighting evolutionarily conserved DNA replication mechanisms (Fig. 5a–d).

The MPXV replisome structures enable us to propose a model for poxvirus replication initiation. A recent study suggested head-to-head dimers of E5 hexamers may facilitate DNA unwinding initiation^36^—a step that could provide ssDNA for replisome loading. Initially, the helicase activity of E5 is inhibited by its primase domains. After recruitment of MPXV polymerase to the E5 hexamer, the primase domains are reorganized to open the central channel of the E5 hexamer, enabling E5 helicase activity (Fig. 5e). As the E5 hexamer continues to unwind dsDNA, a longer lagging strand is generated and bound by the MPXV polymerase F8 thumb and E5_A_ primase. The bound lagging-strand ssDNA serves as a template for RNA primer synthesis by the polymerase–primase core complex as a starting point for generation of Okazaki fragments (Fig. 5f).

**Extended Data Fig. 10:**
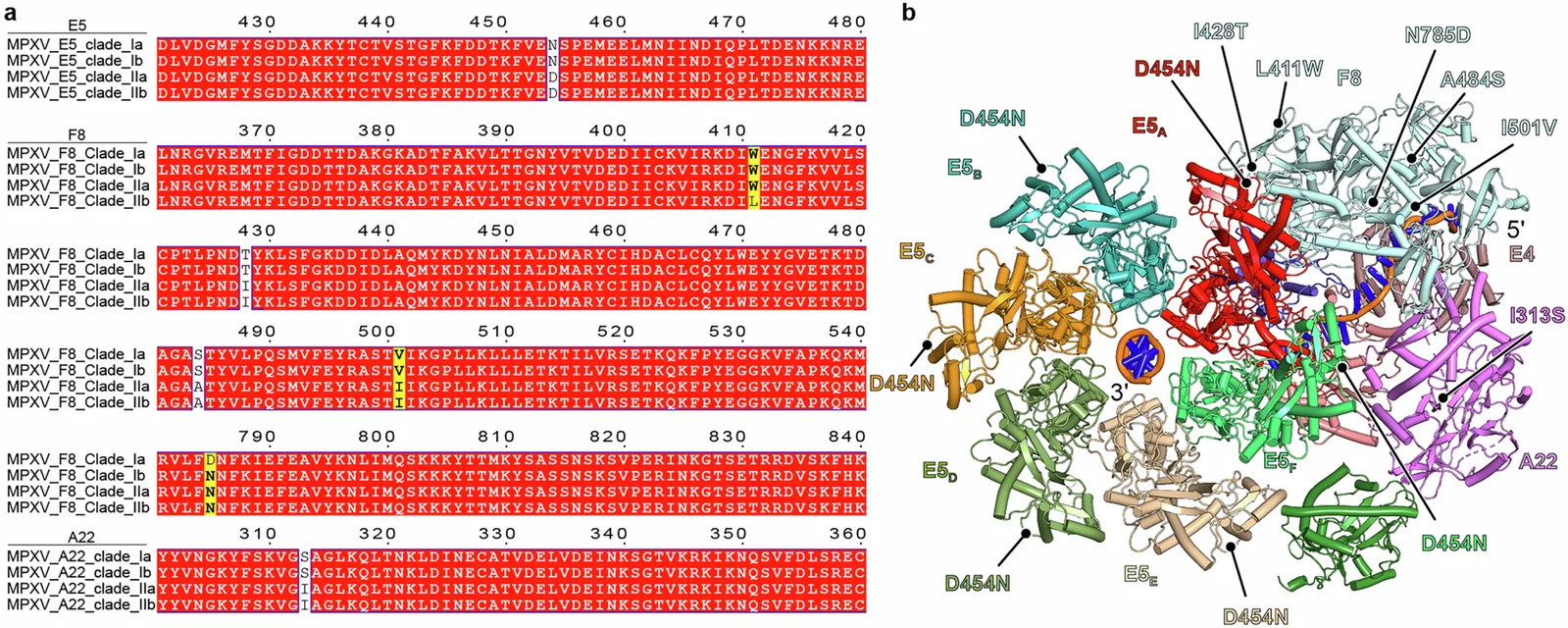
Sequence comparison of MPXV replisome subunits in different clades. **a**, Amino acid sequence alignment of E5, F8, A22 in four clades. GenBank accession numbers for sequences used for the indicated MPXV clades are as follows: clade Ia (NC_003310); clade Ib (PQ762586); clade IIa (PV122073); clade IIb (PV034565). **b**, Variable residues are indicated on the cryo-EM structure of the ssDNA-bound MPXV replisome, which is for a clade IIb isolate. None of the substitutions involve residues found at protein-protein interfaces.

The helicase–primase E5 and the DNA polymerase subunits are highly conserved across MPXV clades; the variable amino acids among MPXV clades and sublineages are not located at the intersubunit interfaces and would not be expected to affect replisome assembly (Extended Data Fig. 10).

A limitation of the study is the high force that we used to tether DNA in the single-molecule assays (52 pN). However, we observed no discernible helicase activity at lower, more physiologically relevant forces (5 and 20 pN) (Supplementary Fig. 2c,d). With the more active E5(ΔRRM) construct, we observed weaker activity at 5 and 20 pN (Supplementary Fig. 3). We believe the higher forces may lower the energy barrier for viral helicase activity under these experimental conditions. Future work will be required to determine whether using a forked DNA substrate or adding the poxviral ssDNA-binding protein in single-molecule experiments influences helicase efficiency.

Notably, a previous study suggested that poxviruses can recruit PCNA (and its associated factors) to the sites of poxvirus DNA replication^37^, but our working model suggests that, with a few exceptions (such as cellular topoisomerase II)^38,39^, poxviruses encode all the machinery necessary to carry out DNA replication. Our findings help to elucidate the molecular mechanisms governing poxvirus DNA replication, potentially providing targets for antiviral therapies. The conformations visualized may serve as a starting point for structure-based virtual screens for small-molecule inhibitors^40,41^. Inhibitors may be used to stabilize or lock conformational intermediates of the complex. This strategy is analogous to the helicase–primase inhibitors against herpesviruses, which bind at the interface of the helicase and primase domains and lock the viral helicase–primase in an inactive conformation^42–45^.

## Methods

### Cells

We maintained Expi293F cells (Thermo Fisher Scientific, A14527) in Expi293 Expression Medium (Thermo Fisher Scientific, A1435101) according to the manufacturer’s instructions. The absence of mycoplasma contamination was verified using the e-Myco PCR detection kit (Bulldog Bio, 25234), with testing performed monthly.

### Protein expression and purification

All MPXV protein sequences were derived from MPXV isolate hMPXV/P12/2022 (clade IIb). The sequence encoding full-length MPXV E5 (GenBank: XNX20584.1, residues 1–785), E5(ΔRRM) (GenBank: XNX20584.1, residues 238–785), F8 (GenBank: XNX20538.1, residues 1–1006), F8 with a C-terminal deletion (GenBank: XNX20538.1, residues 1–984) or E4 (GenBank: XNX20583.1, residues 1–218) was cloned into the pCAGGS vector containing a maltose-binding protein (MBP) at the N terminus followed by HRV 3C cleavage site (LEVLFQGP). The sequence encoding wild-type A22 (GenBank: XNX20615.1, residues 1–426) or A22 with the F217A and F263A substitutions (GenBank: XNX20615.1, residues 1–426) were cloned into a pCAGGS vector without tag. Plasmids encoding MPXV polymerase subunits (F8, A22 and E4) were co-transfected into Expi293F cells maintained in suspension using polyethylenimine (PEI) (25000 MW, Polysciences), and E5 was transfected into Expi293F cells grown in suspension using PEI (25000 MW, Polysciences) when the cells reached a density of 2 × 10^6^ cells per ml.

For wild-type MPXV polymerase holoenzyme (F8–A22–E4) and MPXV polymerase holoenzyme mutant purification, after culturing at 37 °C for 72 h, cells were collected by centrifugation at 4,000*g* for 20 min. Cells were lysed using lysis buffer containing 50 mM HEPES-NaOH, pH 7.5, 300 mM NaCl, 0.5% (v/v) Triton X-100, 5 mM MgCl_2_, 0.5 mM EDTA, 1 mM DTT and protease inhibitor (cOmplete, Mini, EDTA-free protease inhibitor cocktail, Millipore Sigma, 11836170001). Cell debris was removed through centrifugation 50,000*g* for 2 h on the Ti50.2 rotor. The supernatant was incubated with amylose resin (NEB, E8021S) at 4 °C for 1 h and washed with wash buffer (25 mM HEPES-NaOH, pH 7.5, 300 mM NaCl, 5 mM MgCl_2_, 0.5 mM EDTA and 1 mM DTT). Bound MPXV F8–A22–E4 complex was subjected to on-column digestion overnight with HRV-3C protease (TaKaRa, 7360) at 4 °C. The eluate fractions within the elution buffer (25 mM HEPES-NaOH, pH 7.5, 150 mM NaCl, 2 mM MgCl_2_ and 1 mM DTT) were concentrated to 500 μl for further purification using Superdex 200 increase column (Cytiva, 28990944). Fractions containing MPXV polymerase holoenzyme were pooled, concentrated to about 1.5 μg μl^−1^ and stored for the further structural and functional studies. For exonuclease-defective polymerase mutant, residues Asp166 and Glu168 in F8 were substituted to alanine using site-directed mutagenesis of the wild-type F8 pCAGGS vector, and the same purification strategy was used to purify the polymerase holoenzyme.

For MPXV E5 or E5(ΔRRM) purification, after culturing at 37 °C for 72 h, cells were collected by centrifugation at 4,000*g* for 20 min. Cells were lysed using lysis buffer containing 50 mM HEPES-NaOH, pH 7.5, 500 mM NaCl, 0.5% (v/v) Triton X-100, 5 mM MgCl_2_, 0.5 mM EDTA, 1 mM DTT and protease inhibitor (cOmplete, Mini, EDTA-free protease inhibitor cocktail, Millipore Sigma, 11836170001). Cell debris was removed through centrifugation 50,000*g* for 2 h with Ti50.2 rotor. The supernatant was incubated with amylose resin (NEB, E8021S) at 4 °C for 1 h and washed with wash buffer (25 mM HEPES-NaOH, pH 7.5, 500 mM NaCl, 5 mM MgCl_2_, 0.5 mM EDTA and 1 mM DTT). Bound MPXV E5 or E5(ΔRRM) were subjected to on-column digestion overnight with HRV-3C protease (TaKaRa, 7360) at 4 °C. The eluate fractions with the elution buffer (25 mM HEPES-NaOH, pH 7.5, 150 mM NaCl, 2 mM MgCl_2_ and 1 mM DTT) were concentrated to 1 ml for further purification using the Superdex 200 increase column (Cytiva, 28990944). The fractions containing MPXV E5 or E5(ΔRRM) hexamers were pooled, concentrated to about 3 μg μl^−1^ and stored for the further structural and functional studies. For the primase-dead E5 mutant, Asp70 in E5 was substituted to alanine using site-directed mutagenesis of the wild-type E5 pCAGGS vector, and the same purification strategy was used. All purified proteins and complexes were analysed using SDS–PAGE.

### MPXV replisome assembly

To determine the structure of the ssDNA-bound MPXV replisome, we used an 80 nucleotide DNA template (5′-TTTTTTTTTTTTTTTTTTTTTTTTTTTTTTTTTTTTTTTTGGCTCCCGCGTCGGAGTCGTTTCGACTCCGACGCGGGAGC-3′) (template 1) (Extended Data Fig. 1c and Supplementary Table 2). The E5 helicase–primase, polymerase (F8–A22–E4) and DNA were mixed at 1:1.2:1.2 molar ratio in a buffer containing 25 mM HEPES-NaOH, pH 7.5, 150 mM NaCl, 2 mM MgCl_2_ and 1 mM DTT overnight. The mixture was applied onto the Superdex 200 increase column (Cytiva, 28990944). The fractions containing MPXV replisome were pooled and concentrated to about 0.3 μg μl^−1^ for subsequent analyses.

To determine the structure of the forked DNA-bound MPXV replisome, we used an 83 nucleotide DNA template (5′-TTTTTTTTTTTTTTTTTTTTTTTTTTTTTTGTCGGAGTCGTTTCGACTCCGACTTTTTTTTTTTTTTTTTTTTTTTTTTTTTT-3′) (template 2) (Extended Data Fig. 5b and Supplementary Table 2). The E5 helicase–primase, polymerase (F8–A22–E4) and DNA were mixed at 1:1.2:1.2 molar ratio in a buffer containing 25 mM HEPES-NaOH, pH 7.5, 150 mM NaCl, 2 mM MgCl_2_ and 1 mM DTT overnight. The mixture was applied onto the Superdex 200 increase column (Cytiva, 28990944). The fractions containing MPXV replisome were pooled and concentrated to about 0.3 μg μl^−1^ for subsequent analyses.

To determine the structure of the MPXV replisome bound to RNA–DNA hybrid, we used a 58 nucleotide DNA oligo (5′-TTTTTTTTTTTTTTTTTTTTTTTTTTTTTTTTTTTTTTTTGCTCCCGCGTCGGAGTCG-3′) and an 18 nucleotide RNA oligo (5′-CGACUCCGACGCGGGAGC-3′) (template 3) (Extended Data Fig. 9b and Supplementary Table 2). The E5 helicase–primase, polymerase (F8–A22–E4) and RNA–DNA hybrid were mixed at 1:1.2:1.2 molar ratio in a buffer containing 25 mM HEPES-NaOH, pH 7.5, 150 mM NaCl, 2 mM MgCl_2_ and 1 mM DTT with overnight incubation. The mixture was passed onto the Superdex 200 increase column (Cytiva, 28990944). The fractions containing MPXV replisome were pooled and concentrated to about 0.3 μg μl^−1^ for subsequent structural analysis.

### Cryo-EM sample preparation and data collection

Samples in the elution buffer (25 mM HEPES-NaOH, pH 7.5, 150 mM NaCl, 2 mM MgCl_2_ and 1 mM DTT) were vitrified using a Vitrobot Mark IV (Thermo Fisher Scientific), with samples maintained at 100% humidity at room temperature. We applied 4 µl of sample to Quantifoil Au 1.2/1.3 300 mesh (EMS Q450CR1.3) grids that were previously plasma treated in a PELCO easiGlow discharge cleaning system at 0.39 mBar, 15 mA, for 30 s and used blot times of 3 s.

We collected datasets using EPU (v.2.13) on a Titan Krios (Thermo Fisher Scientific) operating at 300 kV and a Falcon 4 detector with Selectris energy filter (Thermo Fisher Scientific) in counting mode at ×165,000 magnification, corresponding to calibrated pixel size of 0.74 Å px^−1^. For the ssDNA-bound (template 1) MPXV replisome dataset, 20,997 micrographs were collected at a dose rate of 8.88 e^–^ px^−1^ s^−1^. The total exposure time of 3.28 s was divided into 56 frames (total dose of approximately 54 e^−^ Å^−2^). For the forked DNA-bound (template 2) MPXV replisome dataset, 45,400 micrographs were collected with the pixel size of 0.74 Å px^−1^ at a dose rate of 10.92 e^−^ px^−1^ s^−1^. The total exposure time of 2.55 s was divided into 87 frames (total dose of approximately 51 e^−^ Å^−2^). For the structure of the MPXV replisome bound to RNA–DNA hybrid (template 3), 27,692 micrographs were collected with the pixel size of 0.74 Å px^−1^ at a dose rate of 11.02 e^−^ px^−1^ s^−1^. The total exposure time of 2.52 s was divided into 43 frames (total dose of approximately 51 e^−^ Å^−2^).

### Cryo-EM data processing

We performed all image processing using Relion 3.0 (v.3.1.4)^47^ and cryoSPARC (v.4.4.1)^48^. For datasets collected on the MPXV replisome with DNA, video frames were gain-normalized and motion-corrected using MotionCor2 (v.1.5.0)^49^. Contrast transfer function (CTF) correction was performed using CTFFind4.1 (v.4.1.14)^50^, as implemented in Relion 3.0.

For the ssDNA-bound MPXV replisome, we performed automated particle picking with 4,073,693 particles (Extended Data Fig. 1d). After several rounds of two-dimensional (2D) classification, a subset of particles (186,468 in total) generated from the first round of heterogeneous refinement of 1,210,465 particles was subjected to second-round heterogeneous refinement. A subset of particles (81,800 in total) generated from the second round of heterogeneous refinement was subjected to a third round 3D classification, and a subset of particles (56,874 in total) generated from the 3D classification was processed for homogeneous refinement, yielding a final map of 4.1 Å resolution. To obtain higher-resolution maps for MPXV replisome, we masked the E5 helicase module and polymerase–primase module for further local refinement, yielding the final maps of 3.8 Å and 3.5 Å with improved density, respectively (Extended Data Fig. 1d–g). After several rounds of 2D classification, a subset of particles (30,240 in total) generated from the first round of heterogeneous refinement of 1,210,465 particles was subjected to local refinement, yielding a MPXV polymerase–primase core complex map of 4.0 Å resolution (Extended Data Fig. 1h).

For the forked DNA-bound MPXV replisome, after several rounds of 2D classification, a subset of particles (31,005 in total), which had been generated from the second round of heterogeneous refinement of 175,757 particles, was subjected to homogeneous refinement, yielding a final map of 6.5 Å resolution (Extended Data Fig. 5a). To obtain higher-resolution maps for forked DNA-bound MPXV replisome, we masked the E5 helicase module and polymerase–primase module for further local refinement, yielding the final maps of 4.5 Å and 4.1 Å with improved density, respectively (Extended Data Fig. 5c–e).

For the structure of the MPXV polymerase–primase core complex bound to RNA–DNA hybrid, after several rounds of 2D classification, a subset of particles (25,351 in total), which had been generated from the second round of heterogeneous refinement of 61,436 particles, was subjected to homogeneous refinement, yielding a final map of 3.8 Å resolution (Extended Data Fig. 9c,d).

### Model building, refinement and figure generation

Two previous cryo-EM structures of ssDNA-bound E5 hexamers differed in the polarity of the ssDNA within E5 (refs. ^3,4^). Of these previous structures, to aid with initial model building, we used the higher-resolution structure (PDB: 8XJ7)^4^, as the ssDNA polarity was consistent with that observed in other superfamily-3 helicases^51,52^. We also used the cryo-EM structure of MPXV polymerase (PDB: 8HG1)^5^. These were fitted as initial models into cryo-EM map using UCSF Chimera X (v.1.2)^53^. E5_B_, E5_C_, E5_D_ and E5_E_ residues 1–323, which comprise the primase domain of E5, could also not be visualized in cryo-EM maps of the complex. To build the model of the ssDNA-bound replisome, we used the 3.5 Å map with mask focused on the polymerase–primase region to build the model of polymerase, E5_A_ primase and E5_F_ RRM. The E5_F_ RRM had weak density but we could nonetheless dock the corresponding coordinates from the E5 cryo-EM structure (PDB: 8HWA)^3^ based on clear density for the α-helices and β-sheets. For E5 helicase, we used the 3.8 Å helicase-focused map for model building. For the interface between E5_A_ ZBM and E5 helicase, we used the 4.1 Å overall map for model building. For the forked DNA-bound MPXV replisome, we used the structure we generated of the ssDNA-bound replisome, which was from higher-resolution maps, as a starting point for model building. For the polymerase–primase core complex bound to RNA–DNA hybrid, we used the 3.8 Å map for model building and rigid body docking, respectively. We performed manual adjustment and iterative model building and real space refinement using Coot (v.0.9.8.8)^54^ and PHENIX (v.1.21-5207)^55^. Figures were generated using PyMol (v.2.5.4) and UCSF Chimera X (v.1.2)^53^. For the structure of the polymerase–primase core complex bound to RNA–DNA hybrid, a longer segment (including 4 bp) for which density could be modelled but was too poor to be deposited was used for figure generation.

### MP analysis

MP analyses were carried out using the Refeyn Two^MP^ mass photometer (Refeyn) at room temperature. Glass coverslips and gaskets were cleaned with HPLC-grade water and isopropanol and dried under filtered gas before use. MPXV E5 alone, polymerase alone, E5 and polymerase with or without DNA substrate and E5 and polymerase containing mutant 2 or 3 subunits were diluted to 200 nM in elution buffer (25 mM HEPES-NaOH, pH 7.5, 150 mM NaCl, 2 mM MgCl_2_ and 1 mM DTT). Next, 18 μl of buffer was used to find the camera focus before loading 2 μl of the sample onto the gasket. The acquisition camera image size was set to medium. Data were collected as a 1 min video and then processed using ratiometric imaging. To correlate ratiometric contrast to mass, the Refeyn Two^MP^ instrument was calibrated using molecular standards of monomeric BSA (66 kDa), dimeric BSA (132 kDa) and thyroglobulin (660 kDa) with a molecular mass error of less than 5%. Data were analysed using Discover^MP^ v.2.3 (Refeyn).

### Helicase activity assay

For helicase activity assays with DNA containing a 5′ overhang, the oligonucleotides 5′-TTTTTTTTTTAGCTACCATGCCTGCACGAATTAAGCAATTCGTAATCATGGTCATAGCT-3′ and 5′-AGCTATGACCATGATTACGAATTGCTTAATTCGTGCAGGCATG-3′ with 5′-labelled 6-FAM were annealed to generate the 5′-overhang-containing DNA substrate (Supplementary Table 2). Helicase assays with 6-FAM-labelled substrates (50 nM) and E5 (0.2, 0.5, 1 and 1.5 μM) were performed in 25 mM HEPES pH 8.0, 50 mM NaCl, 10 mM MgCl_2_, 5 mM ATP and 1 mM dNTP, which was named the E5-only group. For E5-polymerase group, assays were performed with 6-FAM-labelled substrates (50 nM), E5 (1 μM) and polymerase (0.5, 1 and 2 μM) in 25 mM HEPES pH 8.0, 50 mM NaCl, 10 mM MgCl_2_, 5 mM ATP and 1 mM dNTP. The E5-only group and E5–polymerase group were incubated at 4 °C for 1 h, respectively, and then were incubated at 37 °C for 2 h. To terminate reactions, we added 20 mM EDTA, 0.5% (v/v) SDS, 0.2% (v/v) bromophenol blue and 2 μM of an unlabelled DNA strand and transferred to 4 °C for 30 min, and proteins were digested by 2 mg ml^−1^ proteinase K (Roche, 3115887001) at room temperature for 30 min. Products were separated on a 20% polyacrylamide–TBE gel, and gels were exposed to Typhoon FLA 9500 (GE Healthcare) for imaging. We used Image Studio Lite (v.5.2) for gel quantification. To quantify relative ssDNA products, a box of equivalent size to that used for other bands of the same template was drawn at the ssDNA position on the negative-control lane and used to set the background to 0. The resulting values were then normalized to the corresponding value from the lane in which the most positive result was obtained (1 μM E5 with 2 μM polymerase), which was set to 1.

For helicase activity assays with forked DNA substrate, the oligonucleotides 5′-TTTTTTTTTTTTTTTTTTTTAGCTACCATGCCTGCACGAATTAAGCAATTCGTAATCATGGTCATAGCT with 3′-labelled 6-FAM and 5′-AGCTATGACCATGATTACGAATTGCTTAATTCGTGCAGGCATGTTTTTTTTTTTTTTTTTTTT-3′ were annealed to generate the forked DNA substrate (Supplementary Table 2). Helicase assays with 6-FAM-labelled substrates (50 nM) and E5 (0.5, 1 and 1.5 μM) were performed in 25 mM HEPES pH 8.0, 50 mM NaCl, 10 mM MgCl_2_, 5 mM ATP and 1 mM dNTP, which was named the E5-only group. For the E5–polymerase group, assays were performed with 6-FAM-labelled substrates (50 nM), E5 (1 μM) and polymerase holoenzyme (0.5, 1 and 2 μM) in 25 mM HEPES pH 8.0, 50 mM NaCl, 10 mM MgCl_2_, 5 mM ATP and 1 mM dNTP. The E5-only group and E5–polymerase group were incubated at 4 °C for 1 h and were then incubated at 37 °C for 2 h. For all reactions under various conditions, 20 mM EDTA, 0.5% (v/v) SDS and 0.2% (v/v) bromophenol blue were added to terminate the reaction, and the samples were transferred to 4 °C for 30 min. Products were separated on a 20% polyacrylamide–TBE gel, and gels were exposed to Typhoon FLA 9500 (GE Healthcare) for imaging. We used Image Studio Lite (v.5.2) for gel quantification. To quantify relative ssDNA products, a box of equivalent size to that used for other bands of the same templates was drawn at the ssDNA position on the negative-control lane and used to set the background to 0. The resulting values were then normalized to the corresponding value from the lane in which the most positive result was obtained (1 μM E5 with 2 μM polymerase), which was set to 1.

The helicase assays evaluating the effect of polymerase–E5 interface mutations were performed with a DNA substrate containing a 5′ overhang. The oligonucleotides 5′-TTTTTTTTTTAGCTACCATGCCTGCACGAATTAAGCAATTCGTAATCATGGTCATAGCT and 5′-AGCTATGACCATGATTACGAATTGCTTAATTCGTGCAGGCAT G-3′ with 5′-labelled 6-FAM were annealed to generate the 5′-overhang DNA substrate (Supplementary Table 2). Helicase assays were performed with E5 alone (1 μM), E5 (1 μM) with WT polymerase (2 μM) and E5 with polymerase containing mutant 1, 2 or 3 (2 μM) in 25 mM HEPES pH 8.0, 50 mM NaCl, 10 mM MgCl_2_, 5 mM ATP and 1 mM dNTP. The helicase assays comparing wild-type E5 with E5(ΔRRM) were performed with a DNA substrate containing a 5′ overhang and E5 alone (1 μM), E5 (1 μM) with WT polymerase (1 μM) and E5(ΔRRM) (1 μM) in 25 mM HEPES pH 8.0, 50 mM NaCl, 10 mM MgCl_2_, 5 mM ATP and 1 mM dNTP. All of the reactions were incubated at 4 °C for 1 h and then were incubated at 37 °C for 2 h. The time-course helicase assays were performed with E5 (1 μM) with polymerase (2 μM) using 5′-overhang DNA substrate or forked DNA in 25 mM HEPES pH 8.0, 50 mM NaCl, 10 mM MgCl_2_, 5 mM ATP and 1 mM dNTP. To terminate reactions, we added 20 mM EDTA, 0.5% (v/v) SDS, 0.2% (v/v) bromophenol blue and 2 μM of an unlabelled DNA strand and transferred to 4 °C for 30 min. The protein was digested by 2 mg ml^−1^ proteinase K (Roche, 3115887001) at room temperature for 30 min. Products were separated on a 20% polyacrylamide–TBE gel, and gels were imaged on the Typhoon FLA 9500 (GE Healthcare) system. We used Image Studio Lite (v.5.2) for gel quantification. To quantify relative ssDNA products, a box of equivalent size to that used for other bands of the same templates was drawn at the ssDNA position on the negative-control lane and used to set the background to 0. For helicase assays with polymerase mutants, the resulting values were then normalized to the corresponding value from the lane in which the most positive result was obtained (1 μM E5 with 2 μM wild-type polymerase), which was set to 1. For time-course helicase assays using 5′-overhang DNA substrate and forked DNA, the resulting values were then normalized to the corresponding value from the lane in which the most positive result was obtained (1 μM E5 with 2 μM wild-type polymerase using 5′-overhang DNA substrate at 60 min), which was set to 1. For helicase assays with E5(ΔRRM), the resulting values were then normalized to the corresponding value from the lane in which the most positive result was obtained (1 μM E5(ΔRRM)), which was set to 1. We used GraphPad Prism (v.10.1.2) for figure generation and statistical analysis of biochemical assays.

### Polymerase DNA elongation assay

The oligonucleotides 5′-TTTTTTTTTTTTTTTTTTTTAGCTACCATGCCTGCACGAATTAAGCAATTCGTAATCATGGTCATAGCT-3′ and 5′-AGCTATGACCATGATTACGAATTGCTTAATTCGTGCAGGCATG-3′ with 5′-labelled 6-FAM were annealed to generate the 5′-overhang-containing DNA substrate (Supplementary Table 2). Elongation assays were performed with polymerase alone (1 μM), polymerase (1 μM) with WT E5 (1 μM) and polymerase (1 μM) with E5(ΔRRM) (1 μM) in 25 mM HEPES pH 8.0, 50 mM NaCl, 10 mM MgCl_2_ and 5 mM dNTP. The reaction was incubated at 4 °C for 1 h and then was incubated at 37 °C for 2 h. All reactions under various conditions were added 20 mM EDTA, 0.5% (v/v) SDS and 0.2% (v/v) bromophenol blue to terminate the reaction and transferred to 4 °C for 30 min. The protein was digested by 2 mg ml^−1^ proteinase K (Roche, 3115887001) at room temperature for 30 min. Products were separated on a 20% polyacrylamide–TBE gel, and gels were exposed to Typhoon FLA 9500 (GE Healthcare) for imaging. We used Image Studio Lite (v.5.2) for gel quantification. To quantify DNA polymerase elongation activity, a box of equivalent size to that used for other bands of the same templates was drawn at the dsDNA position on the negative-control lane and used to set the background to 0. The resulting values were then normalized to the corresponding value from the lane in which the most positive result was obtained (1 μM polymerase alone), which was set to 1.

### Optical tweezer experiments

Single-molecule experiments were performed on a C-trap (LUMICKS) integrating optical tweezers and microfluidics. The five-channel laminar flow cell was used for experiments after passivation using 0.5% (w/v) Pluronic F127 in PBS, and subsequently with BSA (1 mg ml^−1^).

We performed the experiment with wild-type E5 and exonuclease-defective MPXV polymerase (F8–A22–E4). Streptavidin-coated polystyrene beads (0.005% (w/v); 4.35 µm, Spherotech, SVP-40-5) were injected into channel 1. Biotin-labelled 17 kb DNA molecule containing two nicks (5 kb apart) on one of the strands (about 2 pM) (LUMICKS, 00027) was flowed into channel 2. Buffer A, containing 25 mM HEPES (pH 7.5), 150 mM NaCl, 0.1 mg ml^−1^ BSA and 1 mM DTT, was injected into channel 3. E5 was diluted to 10 nM in buffer A and injected into channel 4. In the end, E5 and polymerase (exonuclease deficient) were diluted to 10 nM and 20 nM in buffer A, respectively, and injected into channel 5.

We conducted single-molecule experiments using wild-type E5 and an exonuclease-deficient polymerase on a five-channel microfluidic chip, loading reagents sequentially as follows: streptavidin-coated polystyrene beads (0.005% (w/v), 4.35 µm; Spherotech) were introduced into channel 1, a biotin-labelled 17 kb DNA substrate containing two nicks spaced around 5 kb apart on one strand (around 2 pM; Lumicks) was flowed into channel 2, and buffer A (25 mM HEPES, pH 7.5, 150 mM NaCl, 0.1 mg ml^−1^ BSA, 1 mM DTT) was loaded into channel 3 for baseline establishment and subsequent washing/equilibration; channels 4 and 5 were then used for protein delivery, whereby E5 alone (10 nM), polymerase alone (20 nM), E5 (10 nM) + polymerase (20 nM), E5 (10 nM) + polymerase (100 nM), and an E5(ΔRRM) (10 nM) were alternately introduced into the same channel(s) across measurements, and whenever switching between different protein conditions within a given channel, the system was extensively flushed with buffer A and re-equilibrated to minimize carryover and ensure consistent assay conditions.

All experiments were performed at room temperature (28 °C). The optical traps were calibrated using power spectrum of Brownian motion of the trapped beads to achieve a trap stiffness of 0.16–0.18 pN nm^−1^. After optically trapping two beads, the DNA molecule was tethered between the beads under flow in channel 2. The presence of a single DNA tether was verified by measuring a force–extension curve at a constant pulling rate of 0.2 µm s^−1^ and comparing it to worm-like chain model of dsDNA. The tethered DNA molecule was extended beyond the contour length of the DNA (6.34 μm) to around 8.5 μm and held for 10 s in the presence of flow to melt away a piece of ssDNA leaving behind a gap of 5 kb on the tethered DNA molecule. Subsequently, the DNA tether was moved to the protein channel (channel 5) and incubated for 10–30 s. In most of the experiments, this loading step was performed using DNA held at very low force (~1 pN). After protein loading, unwinding experiments were performed in the same channel under a constant force of 52 pN, with the resulting changes in distance between beads recorded using BlueLake software (v.2.6.4) for subsequent analysis. To ensure reproducibility across different conditions, the experiments were repeated for a total of 33 times for the E5:polymerase 1:2 ratio; 19 times for the E5:polymerase 1:10 ratio; and 25 times for the E5(ΔRRM) mutant (Supplementary Figs. 1d and 2a,b). Notably, measurements conducted with an E5:polymerase ratio of 1:2 at lower forces of 5 pN and 20 pN (*n* = 12 and 10, respectively) yielded no detectable unwinding activity, and measurements conducted with the E5(ΔRRM) mutant at 5 pN and 20 pN (*n* = 8 and 9, respectively) yielded weak unwinding activity (Supplementary Figs. 2c,d and 3).

To confirm the mechanical stability of the substrate, we performed control experiments at 52 pN in the absence of protein (Supplementary Fig. 1a). These measurements showed no evidence of spontaneous DNA unwinding or mechanical rupture, confirming that the observed activity was protein dependent. Moreover, we conducted controls using E5 alone (*n* = 19) and polymerase alone (*n* = 12) (Supplementary Fig. 1b,c), neither of which showed processive unwinding comparable to the MPXV E5 with polymerase or the E5(ΔRRM) protein alone.

### Data acquisition and analysis

All data analysis was carried out in Python using custom-written scripts and Lakeview (v.1.3). Helicase activity, recorded as the change in distance between optically trapped beads, was converted to the number of nucleotides unwound^42^. From each raw trace, multiple 15 s windows (around 250 datapoints per window) were extracted. These segments were smoothed using a Savitzky–Golay filter, after which a linear regression was performed on each window to estimate the unwinding rates. To identify pause states, the raw data were first filtered using the Savitzky–Golay filter to reduce noise. The discrete derivative of the unwound nucleotides with respect to time was then computed. A threshold of 2 nucleotides per second was used. The timepoints with instantaneous unwinding rates below this threshold were classified as paused. The first 60 s of the unwinding trace was used for quantification of paused state duration (Supplementary Fig. 1e). Statistical comparisons between experimental conditions were performed using Welch’s *t*-tests. Pairwise comparisons were conducted as indicated in the text. All statistical analyses were implemented in Python using standard scientific libraries.

### E5 primase RNA synthesis assay

To measure nucleotide synthesis, 2 µM of WT E5 and 4 µM or 0.02–4 µM of WT polymerase holoenzyme were incubated with 15 µM of M13 ssDNA (NEB, N4040S) on ice for 60 min. To measure nucleotide synthesis inhibition by mutant E5 or polymerase, 2 µM of E5(D70A) (primase dead) or E5(ΔRRM), and 10 µM WT, mutant 2 or mutant 3 polymerase holoenzyme were incubated with 15 µM of M13 ssDNA on ice for 60 min. The protein–DNA mixture was incubated with unlabelled 1,000 µM ATP (Jena Bioscience), 250 µM GTP, 250 µM UTP, 25 µM CTP (New England Biolabs, N0450L) and 0.5 µM α^32^P-labelled CTP (Perkin Elmer/Revvity, approximately 1 µCi) in reaction buffer (25 mM Tris-HCl pH 7.5, 30 mM KCl, 10 mM MgCl_2_, 10 mM DTT, 100 µg ml^−1^ BSA) at 37 °C for 60 min. Reaction mixtures (6 µl) were boiled at 80 °C for 10 min and terminated with addition of 0.5 µl of Quick CIP phosphatase (New England Biolabs, M0525S) at 37 °C for 90 min to remove terminal phosphate groups from unreacted nucleotides. Where indicated, reactions were additionally treated with 1 µl DNase I (Thermo Fisher Scientific, EN0521) or 1 µl RNase T1 (Thermo Fisher Scientific, AM2283) and 1 µl RNase A (NEB, T3018L). To visualize the ladder, 25 µM ssRNA (IDT) with scrambled sequence of varying nucleotide lengths (10, 15, 20, 25, 30, 40, 50, 60 nucleotides) was labelled with 10 U of T4 PNK (NEB, M0201L), 0.5 µM γ^32^P-labelled ATP (Perkin Elmer/Revvity, approximately 20 µCi) in reaction buffer (70 mM Tris-HCl pH 7.6, 10 mM MgCl_2_, 5 mM DTT) at 37 °C for 60 min. Each reaction was denatured with 2× STOP loading dye (95% (v/v) deionized formamide, 20 mM EDTA, 0.01% (v/v) bromophenol blue and xylene cyanol) and analysed on a 20% urea–PAGE gel and exposed to a phosphor-screen before imaging on the Typhoon Trio Variable Mode Imager (GE Healthcare).

### Reporting summary

Further information on research design is available in the Nature Portfolio Reporting Summary linked to this article.

## Online content

Any methods, additional references, Nature Portfolio reporting summaries, source data, extended data, supplementary information, acknowledgements, peer review information; details of author contributions and competing interests; and statements of data and code availability are available at https://doi.org/10.1038/s41586-026-10937-2.

## Supplementary information

Supplementary information Supplementary Tables 1–6 and Supplementary Figs. 1–4, and their accompanying legends. The tables report MP measurements, the results of previously published mutational analyses on E5 primase, the sequences of DNA substrates and cryo-EM data collection and processing statistics. The figures show optical tweezer unwinding traces and analyses and uncropped gel images.

Reporting Summary

Supplementary Data Source data for Supplementary Fig. 3.

Peer Review File

## Source data

Source Data Fig. 2

Source Data Extended Data Fig. 7

## Data Availability

PDB and Electron Microscopy Data Bank (EMDB) identification numbers for the cryo-EM structures and maps reported in this Article are available under accession codes 9NSP and EMD-49747 (MPXV replisome bound to ssDNA); 10WP and EMD-75504 (MPXV replisome bound to forked DNA); 11WZ and EMD-76148 (MPXV polymerase–primase core complex with RNA–DNA hybrid). Images of uncropped gels are provided (Supplementary Fig. 4). Source data are provided with this paper.

## Code availability

The script used for C-trap data analysis is available at Zenodo^56^ (https://doi.org/10.5281/zenodo.21400187).
