# Supplementary material for: Structure and operating principles of a monkeypox virus replisome
Source: Nature. 2026 Sep 2;658(8135):547–55. doi: 10.1038/s41586-026-10937-2 (PMC13645618; doi:10.1038/s41586-026-10937-2)
Supplement: Supplementary file 1 [file 41586_2026_10937_MOESM1_ESM.pdf]

nature portfolio

https://doi.org/10.1038/s41586-026-10937-2

Supplementary information

Structure and operating principles of a monkeypox virus replisome

In the format provided by the authors and unedited

Structure and operating principles of an mpox virus replisome

Zishuo Yu[1], Pradeep Sathyanarayana[2], Joel M.J. Tan[1,3], Side Hu[1], Xiaoyi Fan[1], Angela Gao[1,3], Philip J. Kranzusch[1,3], Joseph J. Loparo[2], Jonathan Abraham[1,4,5,6,*]

[1]Department of Microbiology, Harvard Medical School, Boston, MA, USA.
[2]Department of Biological Chemistry and Molecular Pharmacology, Blavatnik Institute, Harvard Medical School, Boston, MA, USA.
[3]Department of Cancer Immunology and Virology, Dana-Farber Cancer Institute, Boston, MA, USA.
[4]Department of Medicine, Division of Infectious Diseases, Brigham & Women's Hospital, Boston, MA, USA.
[5]Center for Integrated Solutions for Infectious Diseases, Broad Institute of Harvard and MIT, Cambridge, MA, USA.
[6]Howard Hughes Medical Institute, Boston, MA, USA.

*correspondence: jonathan_abraham@hms.harvard.edu

Table of contents:

Supplementary Table 1. Expected masses of subunits and complexes and mass photometry results. 2

Supplementary Table 2. DNA substrates for functional assays and cryo-EM structure determination. 2

Supplementary Table 3. Cryo-EM data collection and image processing for MPXV replisome bound to ssDNA. 3

Supplementary Table 4. Cryo-EM data collection and image processing for MPXV replisome bound to forked DNA. 4

Supplementary Table 5. Summary of previously published mutational analyses of poxvirus primase active site. 5

Supplementary Table 6. Cryo-EM data collection and image processing for MPXV polymerase-primase core complex (RNA/DNA hybrid). 5

Supplementary Fig. 1. Optical tweezer traces for experiments performed with no protein, E5 alone, polymerase alone, or E5 with polymerase in a 1:2 ratio. 6

Supplementary Fig. 2. Optical tweezer traces for experiments performed with E5 and polymerase 1:10 ratio, E5$^{\Delta RRM}$, and E5 and polymerase at lower forces. 7

Supplementary Fig. 3. Optical tweezer traces and unwinding lengths for experiments performed with E5$^{\Delta RRM}$ at different forces. 8

Supplementary Fig. 4. Uncropped gel images. 9

Supplementary Table 1. Expected masses of subunits and complexes and mass photometry results.

Complex	Subunit masses	Expected stoichiometry	Expected Complex mass	Measured mass
MPXV E5 helicase–primase	90 kDa (E5)	Hexamer	540 kDa	478 kDa s.d. of ± 30 kDa
MPXV polymerase (F8/A22/E4)	117 kDa (F8)<br>49 kDa (A22)<br>25 kDa (E4)	1:1:1 ratio of F8:A22:E4	191 kDa	193 kDa s.d. of ± 14.5 kDa
DNA-bound MPXV helicase–primase and polymerase (E5+F8/A22/E4) + DNA template 1	90 kDa (E5)<br>117 kDa (F8)<br>49 kDa (A22)<br>25 kDa (E4)<br>25 kDa (DNA)	6:1:1:1 ratio of E5:F8:A22:E4	756 kDa	726 kDa s.d. of ± 53 kDa

Supplementary Table 2. DNA substrates for functional assays and cryo-EM structure determination.

	Sequence
Template 1, DNA used for replisome assembly and ssDNA bound replisome structure	5′-TTT TTT TTT TTT TTT TTT TTT TTT TTT TTT TTT TTT TTT TGG CTC CCG CGT CGG AGT CGT TTC GAC TCC GAC GCG GGA GC-3′
Template 2, DNA used for forked DNA-bound replisome structure	5′-TTT TTT TTT TTT TTT TTT TTT TTT TTT TTT GTC GGA GTC GTT TCG ACT CCG ACT TTT TTT TTT TTT TTT TTT TTT TTT TTT TT-3′
Template 3, DNA used for structure of RNA-DNA hybrid-bound complex	5′-TTT TTT TTT TTT TTT TTT TTT TTT TTT TTT TTT TTT TTT TG CTC CCG CGT CGG AGT CG -3′
RNA primer used for structure of RNA-DNA hybrid-bound complex	5′- CGA CUC CGA CGC GGG AGC -3′
DNA used for helicase assay with 5′ overhang-containing substrate	5′-TTT TTT TTT TAG CTA CCA TGC CTG CAC GAA TTA AGC AAT TCG TAA TCA TGG TCA TAG CT-3′
Primer for helicase assay with 5′ overhang-containing substrate	5′/6-FAM/-AGC TAT GAC CAT GAT TAC GAA TTG CTT AAT TCG TGC AGG CAT G-3′
Unlabeled capture strand	5′-AGC TAT GAC CAT GAT TAC GAA TTG CTT AAT TCG TGC AGG CAT G-3′
DNA used for helicase assay with forked DNA substrate	5′-AGC TAT GAC CAT GAT TAC GAA TTG CTT AAT TCG TGC AGG CAT GTT TTTTTT TTT TTT TTT TTT T-3′
DNA primer for helicase with forked DNA substrate	5′-TTT TTT TTT TTT TTT TTT TTA GCT ACC ATG CCT GCA CGA ATT AAG CAA TTC GTA ATC ATG GTC ATA GCT-3′/6-FAM/
DNA used for polymerase DNA elongation assay with 5′ overhang-containing substrate	5′-TTT TTT TTT TTT TTT TTT TTA GCT ACC ATG CCT GCA CGAATT AAG CAA TTC GTA ATC ATG GTC ATA GCT-3′
Primer for polymerase DNA elongation assay with 5′ overhang-containing substrate	5′/6-FAM/-AGC TAT GAC CAT GAT TAC GAA TTG CTT AAT TCG TGC AGG CAT G-3′

Supplementary Table 3. Cryo-EM data collection and image processing for MPXV replisome bound to ssDNA.

	Replisome bound to ssDNA (EMDB-49747) (PDB 9NSP)			Polymerase–E5 primase core complex (DNA) (EMDB- 76148)
**Data collection and processing**				
Magnification		165,000		165,000
Voltage (kV)		300		300
Electron exposure (e–/Å^2)		54		54
Defocus range (μm)		-1.0 to -2.5		-1.0 to -2.5
Pixel size (Å)		0.74		0.74
Symmetry imposed		C1		C1
Initial particle images (no.)		4,073,693		4,073,693
Final particle images (no.)		56,874		30,240
Maps	Overall map	Helicase domain	Primase domains and polymerase	Polymerase–E5 primase core complex (DNA)
Map resolution (Å)	4.1	3.8	3.5	4.0
FSC threshold		0.143		0.143
**Refinement**				
Initial model used (PDB code)		PDB: 8HG1, PDB: 8HWA, and AlphaFold 3		PDB: 8HG1, PDB: 8HWA, and AlphaFold 3
R.m.s. deviations				
Bond lengths (Å)		0.003		
Bond angles (°)		0.890		
Validation				
Clashscore		5		
Ramachandran plot				
Favored (%)		97.4		
Allowed (%)		2.6		
Disallowed (%)		0		

Supplementary Table 4. Cryo-EM data collection and image processing for MPXV replisome bound to forked DNA.

	Replisome bound to forked DNA (EMDB-75504) (PDB 10WP)		
**Data collection and processing**			
Magnification		165,000	
Voltage (kV)		300	
Electron exposure (e–/Å^2)		54	
Defocus range (μm)		-1.0 to -2.5	
Pixel size (Å)		0.74	
Symmetry imposed		C1	
Initial particle images (no.)		10,274,660	
Final particle images (no.)		17,441	
Maps	Overall map	Helicase domain	Primase domains and polymerase
Map resolution (Å)	6.5	4.5	4.1
FSC threshold		0.143	
**Refinement**			
Initial model used (PDB code)	PDB: 8HG1, PDB: 8HWA, and AlphaFold 3		
R.m.s. deviations			
Bond lengths (Å)		0.009	
Bond angles (°)		1.382	
Validation			
Clashscore		10	
Ramachandran plot			
Favored (%)		94	
Allowed (%)		6	
Disallowed (%)		0	

Supplementary Table 5. Summary of previously published mutational analyses of poxvirus primase active site.

Virus	Primase mutant	Structural role	Effects	Reference
MPXV	D70A<br>D72A<br>D170A	Mg^{2+}-coordinating residues	Decreased primase activity (recombinant protein)	Zhang et al.[4]
MPXV	K130A	Interacts with nucleotide phosphate group	No effect on primase activity (recombinant protein)	Zhang et al.[4]
MPXV	H134A<br>R181A<br>K187A	Interacts with nucleotide phosphate group	Decreased primase activity (recombinant protein)	Zhang et al.[4]
VACV	D70A<br>D72A<br>D170A	Interacts with nucleotide phosphate group	Decreased DNA replication (viral replication assays)	De Silva et al.[13]
VACV	D170A	Mg^{2+}-coordinating residue	Decreased primase activity (recombinant protein)	De Silva et al.[13]

Supplementary Table 6. Cryo-EM data collection and image processing for MPXV polymerase-primase core complex (RNA/DNA hybrid).

	Polymerase-primase core complex (RNA/DNA hybrid) (EMDB-76148) (PDB 11WZ)
**Data collection and processing**	
Magnification	165,000
Voltage (kV)	300
Electron exposure (e–/Å^2)	54
Defocus range (μm)	-1.0 to -2.5
Pixel size (Å)	0.74
Symmetry imposed	C1
Initial particle images (no.)	4,638,938
Final particle images (no.)	25,351
Maps	Polymerase–primase core complex (RNA/DNA hybrid)
Map resolution (Å)	3.8
FSC threshold	0.143
**Refinement**	
Initial model used (PDB code)	PDB: 8HG1, PDB: 8HWA and AlphaFold 3
R.m.s. deviations	
Bond lengths (Å)	0.003
Bond angles (°)	0.854
Validation	
Clashscore	4
Ramachandran plot	
Favored (%)	96.8
Allowed (%)	3.2
Disallowed (%)	0

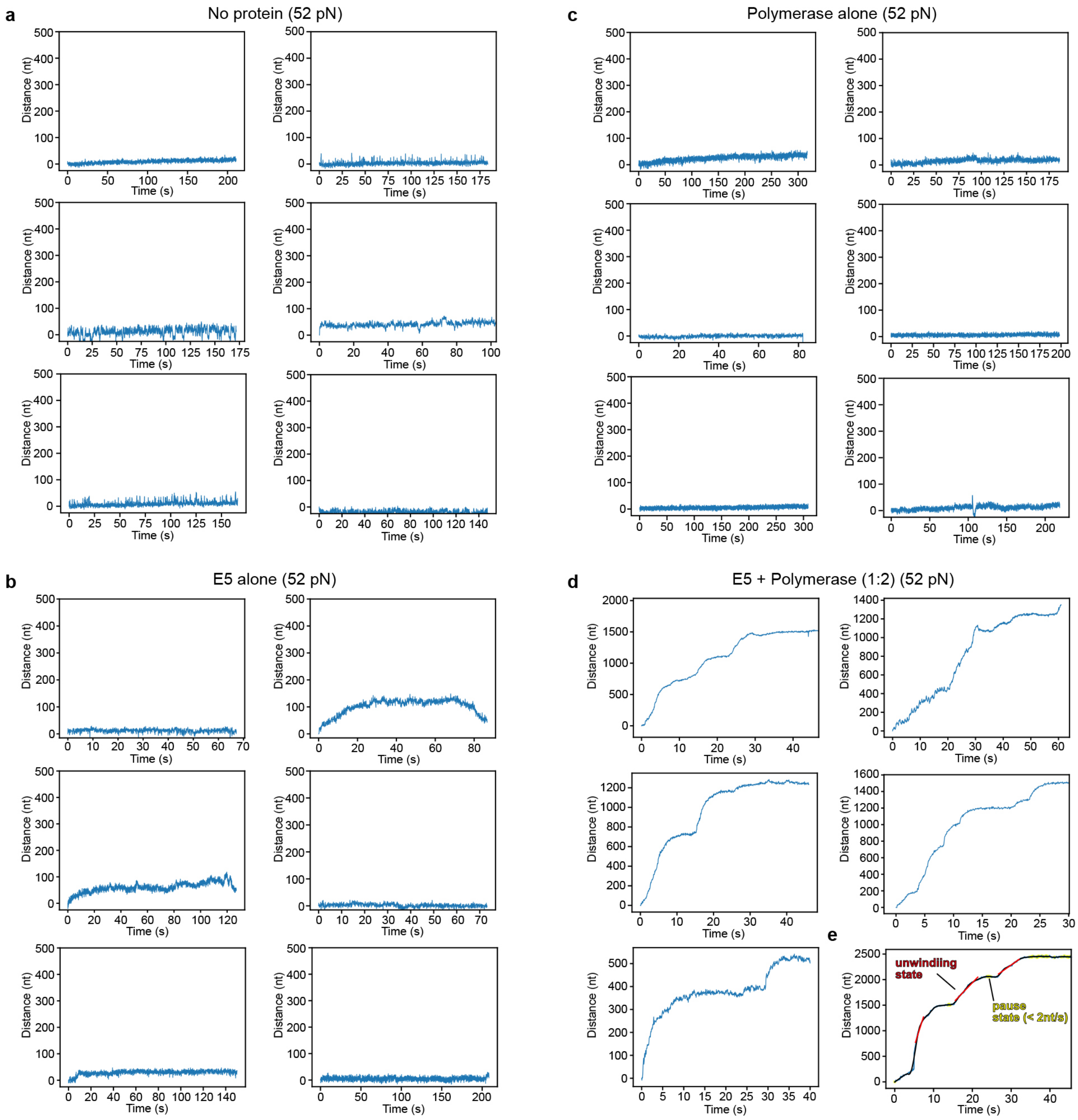

Supplementary Fig. 1. Optical tweezer traces for experiments performed with no protein, E5 alone, polymerase alone, or E5 with polymerase in a 1:2 ratio.
a, Representative traces measured in optical tweezer experiments at 52 pN with no proteins. **b**, Representative traces of unwinding activity in the presence of 10 nM E5 alone. **c**, Representative traces of unwinding activity in the presence of 20 nM polymerase (Exo-deficient F8) alone. **d**, Representative traces of unwinding activity in the presence of 10 nM E5 and 20 nM polymerase (Exo-deficient F8). **e**, Representative trace of optical tweezer data analysis. The paused and unwinding states are labeled after classification based on the instantaneous unwinding rate (see Methods). The original trace is from an experiment that included E5 and polymerase (Exo-deficient) at a 1:2 ratio. In all panels, the force used during the experiments is indicated in parentheses.

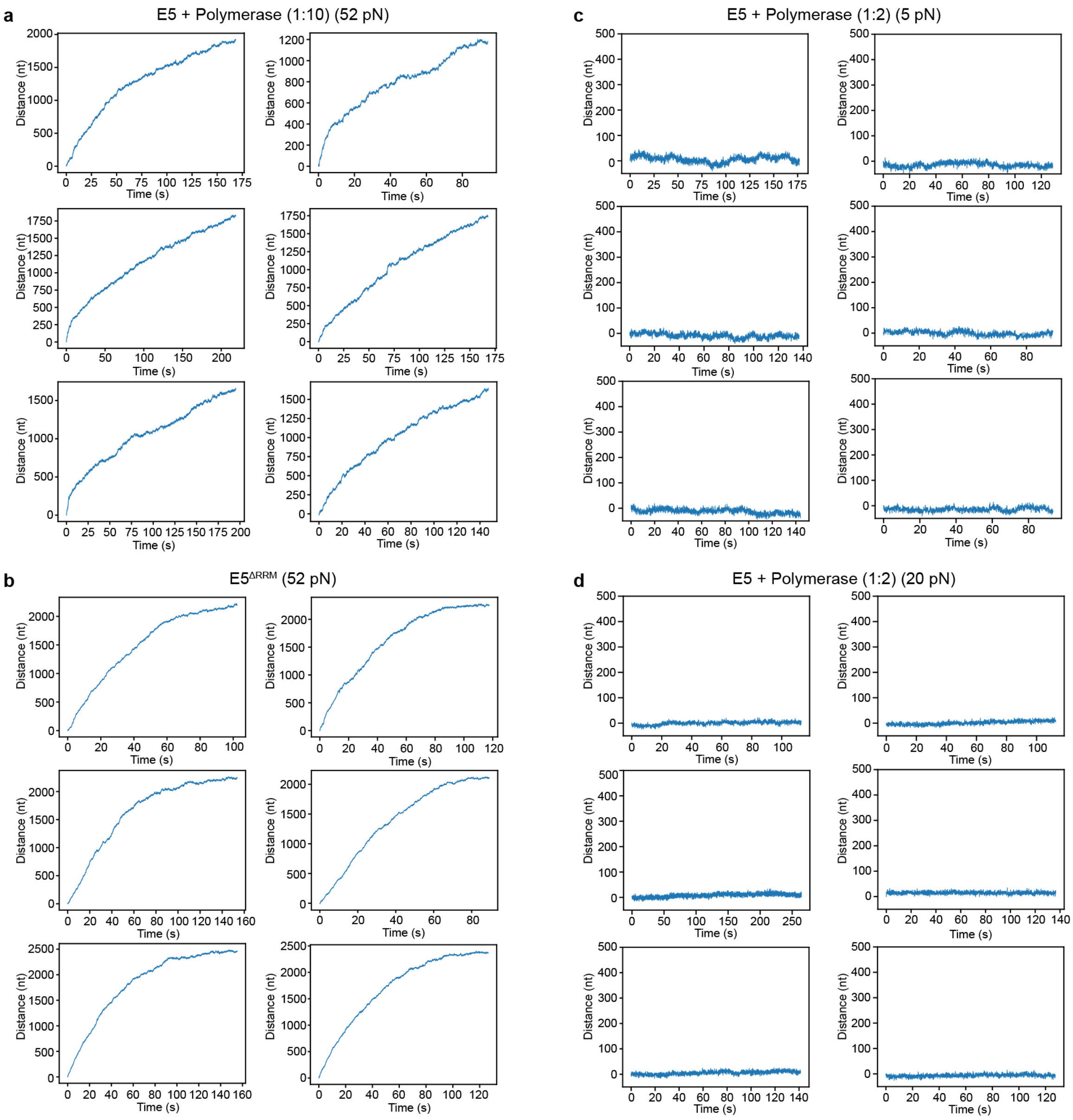

Supplementary Fig. 2. Optical tweezer traces for experiments performed with E5 and polymerase 1:10 ratio, E5$^{\Delta RRM}$, and E5 and polymerase at lower forces. a, Representative traces of unwinding activity in the presence of 10 nM E5 and 100 nM polymerase holoenzyme (Exo-deficient F8). **b**, Representative traces of unwinding activity in the presence of 10 nM E5$^{\Delta RRM}$. **c**, Representative traces of unwinding activity in the presence of 10 nM E5 and 20 nM polymerase (containing Exo-deficient F8) measured at 5 pN. **d**, Representative traces of unwinding activity in the presence of 10 nM E5 and 20 nM polymerase (containing Exo-deficient F8) measured at 20 pN. In all panels, the force used during the experiments is indicated in parentheses.

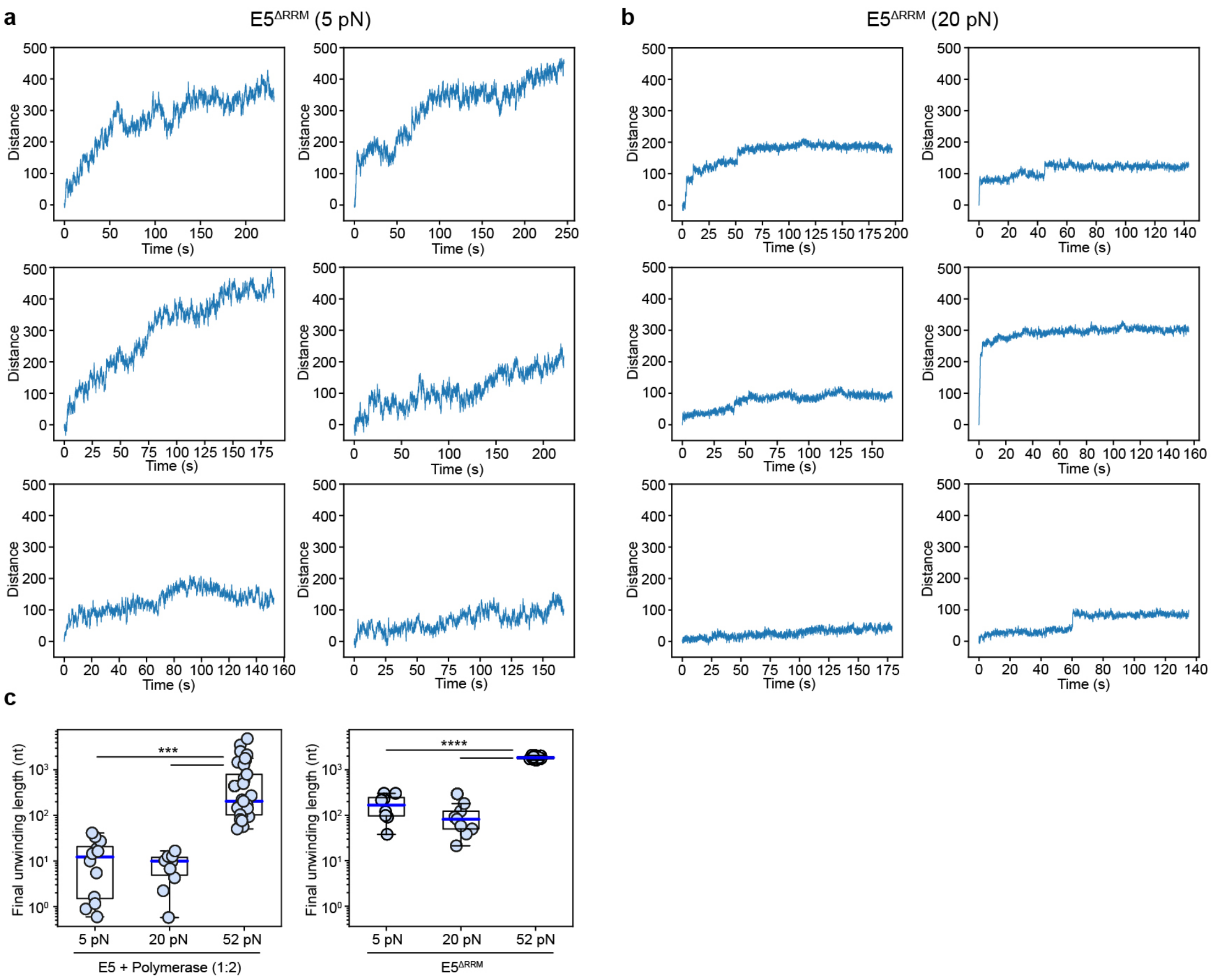

Supplementary Fig. 3. Optical tweezer traces and unwinding lengths for experiments performed with E5$^{\Delta RRM}$ at different forces. a, Representative traces of unwinding activity in the presence of 10 nM E5$^{\Delta RRM}$ measured at 5 pN. **b**, Representative traces of unwinding activity in the presence of 10 nM E5$^{\Delta RRM}$ measured at 20 pN. **c**, Distribution of the unwinding length at 60 s time point of a E5 with polymerase 1:2 ratio or E5$^{\Delta RRM}$ at 5 pN, 20 pN and 52 pN. In **c**, the box spans the interquartile range (IQR; 25th to 75th percentile), the blue line marks the median, and the whiskers extend to the minima and maxima within 1.5 times the IQR. Individual points represent raw data. Statistical comparisons between experimental conditions were performed using Welch's t-test. All statistical tests are two-tailed comparisons. ****$P < 0.0001$, ***$P = 0.000452$ (5 pN) and 0.000416 (20 pN), respectively.

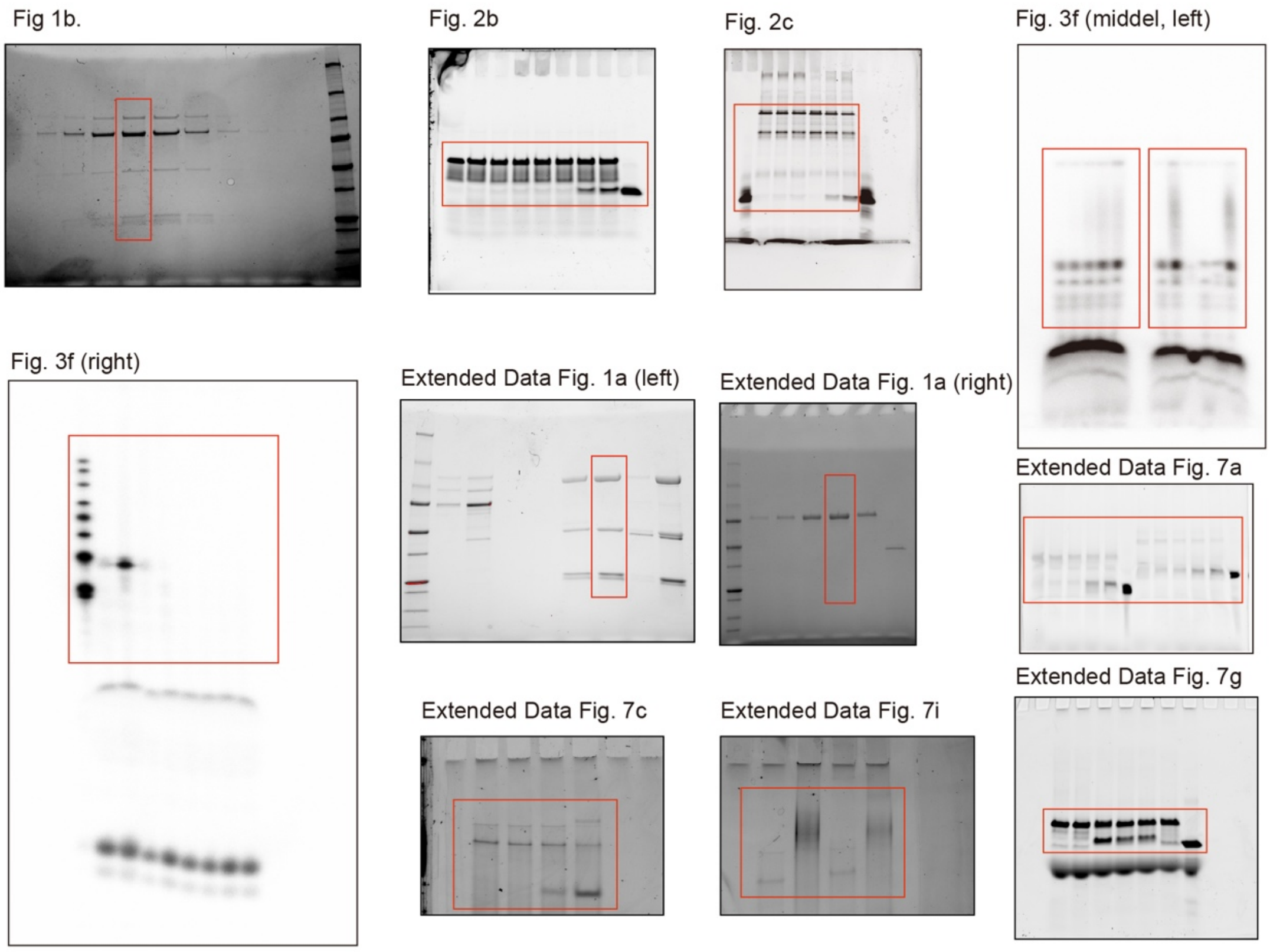

Supplementary Fig. 4. Uncropped gel images.
Red boxes represent the area presented in figures.
