# Supplementary material for: Structure and operating principles of a monkeypox virus replisome
Source: Nature. 2026 Sep 2;658(8135):547–55. doi: 10.1038/s41586-026-10937-2 (PMC13645618; doi:10.1038/s41586-026-10937-2)
Supplement: Supplementary file 2 [file 41586_2026_10937_MOESM2_ESM.pdf]

nature portfolio

Corresponding author(s): Jonathan Abraham

Last updated by author(s): Jul 13, 2026

Reporting Summary

Nature Portfolio wishes to improve the reproducibility of the work that we publish. This form provides structure for consistency and transparency in reporting. For further information on Nature Portfolio policies, see our Editorial Policies and the Editorial Policy Checklist.

Statistics

For all statistical analyses, confirm that the following items are present in the figure legend, table legend, main text, or Methods section.

n/a	Confirmed	
☐	☒	The exact sample size (*n*) for each experimental group/condition, given as a discrete number and unit of measurement
☐	☒	A statement on whether measurements were taken from distinct samples or whether the same sample was measured repeatedly
☐	☒	The statistical test(s) used AND whether they are one- or two-sided<br>*Only common tests should be described solely by name; describe more complex techniques in the Methods section.*
☐	☒	A description of all covariates tested
☐	☒	A description of any assumptions or corrections, such as tests of normality and adjustment for multiple comparisons
☐	☒	A full description of the statistical parameters including central tendency (e.g. means) or other basic estimates (e.g. regression coefficient) AND variation (e.g. standard deviation) or associated estimates of uncertainty (e.g. confidence intervals)
☐	☒	For null hypothesis testing, the test statistic (e.g. *F*, *t*, *r*) with confidence intervals, effect sizes, degrees of freedom and *P* value noted<br>*Give P values as exact values whenever suitable.*
☒	☐	For Bayesian analysis, information on the choice of priors and Markov chain Monte Carlo settings
☒	☐	For hierarchical and complex designs, identification of the appropriate level for tests and full reporting of outcomes
☒	☐	Estimates of effect sizes (e.g. Cohen's *d*, Pearson's *r*), indicating how they were calculated

Our web collection on statistics for biologists contains articles on many of the points above.

Software and code

Policy information about availability of computer code

Data collection	Bluelake (version 2.6.4), EPU (version 2.13).
Data analysis	GraphPad Prism (version 10.1.2), Lakeview (version 1.3), RELION (version 3.1.4), cryoSPARC (version 4.4.1), DeepEMhancer (version 20210511), UCSF ChimeraX (version 1.2), PyMOL (version 2.5.4), Coot (version 0.9.8.8), PHENIX (version 1.21-5207). GitHub web-link:https://github.com/pradeep882006/mPOX-replisome-optical-tweezer-script.git

For manuscripts utilizing custom algorithms or software that are central to the research but not yet described in published literature, software must be made available to editors and reviewers. We strongly encourage code deposition in a community repository (e.g. GitHub). See the Nature Portfolio guidelines for submitting code & software for further information.

Data

Policy information about availability of data

All manuscripts must include a data availability statement. This statement should provide the following information, where applicable:

- Accession codes, unique identifiers, or web links for publicly available datasets
- A description of any restrictions on data availability
- For clinical datasets or third party data, please ensure that the statement adheres to our policy

Structural coordinates and maps are deposited under PDB:9NSP, EMDB:49747, PDB: 10WP; EMDB:75504; PDB:11WZ; EMDB:76148

Research involving human participants, their data, or biological material

Policy information about studies with human participants or human data. See also policy information about sex, gender (identity/presentation), and sexual orientation and race, ethnicity and racism.

Reporting on sex and gender	N/A
Reporting on race, ethnicity, or other socially relevant groupings	N/A
Population characteristics	N/A
Recruitment	N/A
Ethics oversight	N/A

Note that full information on the approval of the study protocol must also be provided in the manuscript.

Field-specific reporting

Please select the one below that is the best fit for your research. If you are not sure, read the appropriate sections before making your selection.

☒ Life sciences ☐ Behavioural & social sciences ☐ Ecological, evolutionary & environmental sciences

For a reference copy of the document with all sections, see nature.com/documents/nr-reporting-summary-flat.pdf

Life sciences study design

All studies must disclose on these points even when the disclosure is negative.

Sample size	For cryo-EM, data collection was determined by sample quality, microscope availability, and the number of particles required to achieve sufficient resolution for reliable reconstruction and model building. For biochemical assays, sample sizes and replicate numbers were based on standard practice in the field and are indicated in the figure legends. For single-molecule C-Trap experiments, the number of molecules/ events analyzed was determined by the availability of stable DNA tethers that met quality-control criteria, and the observed effects were consistent across independent molecules and experiments.
Data exclusions	No data from biochemical assays were excluded.<br>Trajectories of protein diffusing on the DNA in optical tweezers experiments were not included in the statistics if the diffusion was shorter than 5 seconds (not enough data points to fit linear mean-square-displacement).<br>Particles without high-resolution features were discarded during data analysis as noted in the Extended Data. This is standard practice in the field of cryo-EM.
Replication	Helicase assay were performed at least three times independently. RNA synthesis assay were performed at least two times independently. Optical tweezers experiments of MPXV E5 with polymerase with (1:2 ratio) under 52 pN were performed multiple times (n=33 independent experiment),Optical tweezers experiments of MPXV E5 with polymerase with (1:10 ratio) under 52 pN were performed multiple times (n=19 independent experiment),Optical tweezers experiments of MPXV E5 alone under 52 pN were performed multiple times (n=19 independent experiment),Optical tweezers experiments of MPXV polymerase alone under 52 pN were performed multiple times (n=12 independent experiment).Optical tweezers experiments of MPXV E5 RRM deletion under 52 pN were performed multiple times (n=25 independent experiment).Optical tweezers experiments with no protein under 52 pN were performed multiple times (n=16 independent experiment).Optical tweezers experiments of MPXV E5 with polymerase with (1:2 ratio) under 5 pN were performed multiple times (n=12 independent experiment).Optical tweezers experiments of MPXV E5 with polymerase with (1:2 ratio) under 20 pN were performed multiple times (n=10 independent experiment).Optical tweezers experiments of MPXV E5 RRM deletion under 5 pN were performed multiple times (n=8 independent experiment).Optical tweezers experiments of MPXV E5 RRM deletion under 20 pN were performed multiple times (n=9 independent experiments).All attempts at replication were successful.
Randomization	Experiments involved structural, biochemical, and single-molecule analyses of purified proteins and in vitro reconstituted complexes, where samples were not assigned to experimental groups and no treatment allocation was involved. Controls were included in all cases. Therefore, randomization was not relevant to the study.
Blinding	Blinding is not relevant to the experiments presented in this study because the data were generated using biochemical assays and structural analyses with objective, quantitative readouts. Cryo-EM, biochemical data and optical tweezers data were collected and processed identically under the same experimental conditions in an unbiased manner, and sample information did not lead to bias on any sample during the analysis

Reporting for specific materials, systems and methods

We require information from authors about some types of materials, experimental systems and methods used in many studies. Here, indicate whether each material, system or method listed is relevant to your study. If you are not sure if a list item applies to your research, read the appropriate section before selecting a response.

Materials & experimental systems

n/a	Involved in the study
☒	☐ Antibodies
☐	☒ Eukaryotic cell lines
☒	☐ Palaeontology and archaeology
☒	☐ Animals and other organisms
☒	☐ Clinical data
☒	☐ Dual use research of concern
☒	☐ Plants

Methods

n/a	Involved in the study
☒	☐ ChIP-seq
☒	☐ Flow cytometry
☒	☐ MRI-based neuroimaging

Eukaryotic cell lines

Policy information about cell lines and Sex and Gender in Research

Cell line source(s)

Expi293F ThermoFisher Scientific Cat# A14527; Expi293 Expression Medium (Thermo Fisher Scientific Cat#: A1435101)

Authentication

The cell line was obtained directly from commercial vendors but not authenticated once received.

Mycoplasma contamination

We confirmed the absence of mycoplasma in the cell line through monthly testing using an e-Myco PCR detection kit.

Commonly misidentified lines
(See ICLAC register)

None.

Plants

Seed stocks

Report on the source of all seed stocks or other plant material used. If applicable, state the seed stock centre and catalogue number. If plant specimens were collected from the field, describe the collection location, date and sampling procedures.

Novel plant genotypes

Describe the methods by which all novel plant genotypes were produced. This includes those generated by transgenic approaches, gene editing, chemical/radiation-based mutagenesis and hybridization. For transgenic lines, describe the transformation method, the number of independent lines analyzed and the generation upon which experiments were performed. For gene-edited lines, describe the editor used, the endogenous sequence targeted for editing, the targeting guide RNA sequence (if applicable) and how the editor was applied.

Authentication

Describe any authentication procedures for each seed stock used or novel genotype generated. Describe any experiments used to assess the effect of a mutation and, where applicable, how potential secondary effects (e.g. second site T-DNA insertions, mosiacism, off-target gene editing) were examined.
