# Supplementary material for: Structure and operating principles of a monkeypox virus replisome
Source: Nature. 2026 Sep 2;658(8135):547–55. doi: 10.1038/s41586-026-10937-2 (PMC13645618; doi:10.1038/s41586-026-10937-2)
Supplement: Supplementary file 3 [file 41586_2026_10937_MOESM4_ESM.pdf]

nature portfolio

Peer Review File

Structure and operating principles of an mpox virus replisome

Corresponding Author: Dr Jonathan Abraham

Any redactions in this file are there to maintain patient confidentiality, the confidentiality of unpublished data, or to remove third-party material.

This file contains all reviewer reports in order by version, followed by all author rebuttals in order by version.

Version 1:

Reviewer comments:

Referee #1

(Remarks to the Author)
This manuscript presents cryoEM structures of the MPXV replicative helicase (D5) and polymerase (trimeric F8/A22/E24) in complex with ssDNA or an RNA/DNA hybrid. Additionally, it examines the stimulation of helicase and primase activities of D5 by the presence of the polymerase. Recent studies have already reported the structures of the helicase and polymerase, as well as the auto-inhibition of the D5 helicase activity by its primase (RRM) and ZBM domains. The current manuscript introduces the following findings, (1) identifying the interface between helicase D5 (primarily through its RRM and ZBM domains) and the thumb domain of the polymerase F8 subunit, and (2) characterizing the activities of D5 and a low-resolution structure of RNA synthesis facilitated by the polymerase. While these findings complement the existing knowledge, the advances appear incremental, and inconsistencies alongside incomplete structural and functional data (as detailed in the comments below) suggest that the manuscript may not meet the standards for publication in Nature at this time.

1. The DNA molecule used in the structural determination of MPXV helicase-polymerase assembly is a hairpin duplex with a 5′ overhang of 40 Ts (ED Fig. 1c), and not a forked DNA with both 5′ and 3′ overhang, as suggested in Fig. 2a.
2. In the cryoEM structure, the observed DNA appears as two separate pieces of ssDNA without any duplex region. The segments, 10 and 17 nt in length, are associated with D5 and polymerase, respectively, but are not connected in the cryoEM volume. It is unclear if they originate from the same DNA molecule. What is the molecular rationale for interpreting them as the leading (bound by D5) and lagging strand (bound by polymerase) DNA? Where is the duplex DNA? Without a DNA fork, how can a replisome form, pre-initiation or initiating?
3. Is there DNA polymerase activity on the DNA substrate used in the cryoEM study (the hairpin duplex with a 5′ 40-T overhang) in the presence of D5? Do the primase and DNA polymerase compete for the DNA substrate?
4. The DNA used in the solution helicase activity assay is a forked DNA (Fig. 3c) with overhangs on both 5′ and 3′ ends. Why wasn't this DNA used in structural studies? Moreover, the DNA used in helicase activity assays by optical tweezer (Fig. 3d) has an overhang on either the 5′ or 3′ end, but not both. Previous studies on the D5 helicase activity suggest that it exhibits much higher activity on a forked DNA substrate than on substrates with either 5′ or 3′ overhang (Ref. #5). The use of different DNA substrates across various assays (structural studies and two helicase activity assays) complicates direct comparisons, akin to comparing apples and oranges.
5. Previous studies have reported that deleting the primase (RRM) and ZBM domains from D5 removes the autoinhibition of D5 and activates its helicase activity (ref. #4, assayed at a 2:1 enzyme-to-DNA ratio). In the current study, helicase activity is only detectable at 20:1 enzyme-to-DNA ratio (Fig. 3c). Does removal of all six RRM and ZBM domains from the hexameric D5 activate the helicase activity more effectively than the addition of polymerase? How many RRM and ABM are required to block DNA substrate binding to D5? The authors should consider comparing the effects of RRM+ZBM deletion with that of polymerase addition.
There are other issues that need attention:
(a) How are the terms "initiating replisome" (as mentioned in the title) and "pre-initiation state replisome" (as mentioned in the text) defined? These terms do not appear to be standard in the DNA replication field.
(b) Why is the MP measured size of the polymerase trimer 152 kDa, rather than the expected 192 kDa (ED Fig. 1b)?
(c) What are the sizes of RNA synthesized by the primase activity (Fig. 4f)?

(d) How might the two cryoEM structures reported in this study aid in the development of anti-poxviral drugs?

Referee #2

(Remarks to the Author)
Yu et al. use a combination of structural biology, bulk biochemical assays, and single-molecule assays to elucidate the configuration and mechanisms of the MPOX virus replisome. These experiments are important and well-executed, yielding a high-impact study. The authors demonstrate super-complex formation between the Helicase/Primase hexamer D5 and the viral DNA polymerase, which is required for D5 helicase activity. They further show that D5 primase activity is stimulated by polymerase binding. The authors also present a lower-resolution structure of the D5-pol complex that they claim is consistent with the priming/synthesis conformation. The homology to other primases and DNA polymerases, as well as the structural comparisons, is compelling.
Major comments: Mutational analysis is missing. While the difficulty of protein expression and purification is understood, only wild-type proteins are shown despite extensive modeling of protein-protein, protein-DNA, and active sites in the figures. At minimum, mutations (such as interface or active sites) that could test their models should be proposed. This would also allow the authors to address important mechanistic questions, such as whether polymerase binding is sufficient for helicase activity even in a polymerase active site mutant. The structural configurations observed, their similarity to the human replisome, and the activity-based assays are strong. However, given the low-resolution maps, if molecular details are emphasized, they must be supported by mutational data—otherwise, the interpretations should be toned down or the maps should be shown explicitly where interactions and active sites are discussed or modeled.
Small molecules that target large-scale conformational changes are notoriously poor immediate drug candidates. Given the conservation of active site residues and chemistry between viral and cellular replisomes, the drug development implications of this work are not strongly supported. However, this does not diminish the importance of the study or its suitability for Nature. If the authors wish to make claims about drug development, these should be supported with data, such as structural evaluation of molecules that inhibit helicase, primase, or polymerase activity or expanded discussion.
Order-of-addition experiments would further support the authors' structural models with biochemical evidence.
Points to address:
1. Can the authors clarify their argument/models regarding Okazaki fragment/discontinuous synthesis versus the rolling-hairpin model with a figure?
2. Can the authors provide examples of other antiviral development efforts using similar approaches? While they argue that their study will aid drug development, this discussion should be expanded beyond general commentary on large-scale conformational changes and protein-protein interactions.
3. The authors should state that future mutational analysis is required to support the presented structures. Alternatively, they should conduct such analyses or focus the manuscript on general configuration and biochemistry rather than specific molecular interactions.
4. When discussing amino acid interactions (e.g., Fig. 2b–h and Extended Data 8e), the authors should show the corresponding densities to compare with the AlphaFold3 model.
5. Move Extended Data Figures 9 and 10 to the main figures, and relocate Fig. 2b, c, d, e, g, h to the supplement. The structural alignments in Extended Data 10, showing comparisons between viral and cellular replisomes, are of broader interest than Figure 2. Similarly, Extended Data 9 is more relevant for a general audience.
6. Figures 1 and 5 (purification, complex formation, and overall cryo-EM structure): include reported resolutions.
7. Figure 2 (protein-protein and protein-DNA interactions): consider showing orientations -and a zoom out figure of the entire complex- between panels more clearly, as it's difficult to understand the spatial relationships. Most of this figure could be moved to the supplement. In contrast, Extended data Figure S9e–j is essential to the authors' argument and should be in the main figures.
8. Figure 4: Biochemistry demonstrates RNA priming activity of D5, requiring Mg^{2+}, DNA, Pol, and helicase. The product is confirmed as RNA (not DNA) based on RNase vs. DNase sensitivity. However, the authors align D5 with PrimPol but do not mutate key residues—mutational analysis of predicted active site residues should be performed to validate RNA production. At the very least, note that such analysis is required.
9. Figure 5: Structural model of the MPXV replisome. This is a filtered lower-resolution map intended to resolve the DNA/RNA hairpin and is distinct from the structure in Figure 1. Clarify this in the figure legend and provide the resolution.
10. Can the authors show whether binding is separable from helicase activity? For example, can D5 or Pol bind the substrate without catalysis? Given that proteins and substrates are in hand, these experiments should be feasible.
11. Additional biochemical assays would strengthen the manuscript, especially for Figures 3 and 5. Gel shift/DNA binding experiments and order-of-addition assays would help establish whether distinct replication states exist. For example:
o Use rNTPs alone to allow RNA synthesis without dNTPs
o Supply only 3 of the 4 required rNTPs to trap intermediates
o Include crosslinking or mutational trapping strategies These assays are critical given the low resolution of the structures and their central role in the authors' argument about helicase, priming, and synthesis transitions.
12. The authors claim their structures support Okazaki fragment formation, in contrast to the prevailing rolling-hairpin model that lacks lagging strand synthesis. However, Figure 5 could be better supported with figures from Extended data 9 and especially 10 directly to the models of Okazaki fragment formation. Lines 193–197: These residues are modeled based on related structures, and if the goal is to revise the replication model, mutational support is essential or at least more thorough discussion and supportive figures.
13. Mutations in D5A that block polymerase binding would provide biochemical support for the structural model. Such mutations should impair both polymerase binding and helicase activity (as shown in Figure 3). Discuss what mutations could be tested or do the authors have such data?
14. Calculate and report 3D-FSCs for all structures.

15. Include a figure in the Introduction comparing the rolling-hairpin model and the lagging-strand/Okazaki fragment model.

Minor notes:

- In the abstract, clarify that Mpox is a double-stranded DNA virus.
- Extended Data 1b: Some bands are unlabeled. Add a table showing expected vs. observed masses, and note that discrepancies fall within error.
- Extended Data 1c: It's unclear which panel is being referenced—please clarify.
- Figure 1b is referred to before 1a—reorder or correct the reference.
- Label nucleic acid polarity in experimental figures (e.g., Fig. 3) and in structural models (e.g., Fig. 5).
- Page 4, line 101 should be rephrased for clarity. Lines 115–117 are too long—split into two sentences.
- Line 191 and elsewhere: Replace "courses" with "is positioned."
- Lines 209–230: The authors state that the Pol-helicase complex forms only in the presence of DNA (Fig. 1), but order-of-addition data is not provided. Gel shift assays comparing Pol alone, D5 alone, and the complex would strengthen this point, or a citation should be provided if this has been shown previously.
- Lines 252–255: The authors reference mutational analysis of conserved D5 primase residues in the literature. It would be interesting to know whether these residues affect binding and helicase activity, particularly since both Pol and helicase are required for function.
- Figure 4: Since polymerase is shown to promote activity in a dose-dependent manner, the concentration of D5 should be included in the figure and legend so readers can assess relative stoichiometry without referencing the Methods.

Referee #3

(Remarks to the Author)
I co-reviewed this manuscript with one of the reviewers who provided the listed reports.

Referee #4

(Remarks to the Author)
A. Summary - Yu et al. used single particle cryoelectron microscopy to image a complex of MPOX virus proteins. The assemblage comprises the core of the machinery that would be needed to initiate RNA priming and the first steps in DNA synthesis. Perhaps the most important discovery is that the structure shows how a hexamer composed of D5 helicase primase subunits, interacts with an F8/A22/E24 processive polymerase complex. In forming the complex it exposes a channel in the helicase hexamer which then activates the helicase and facilitates primase activity. The work is supported by extensive experimental documentation including mass photometry and helicase activity measurements using an optical tweezer system. Alphafold modeling was also used to extract further insights into the parallels with homologous primer polymerases. Models are proposed to suggest ways in which various structures are loaded, assembled, and elongated.

B. Originality - The paper describes original and significant research. In particular it resolves longstanding controversies over why it's been so difficult to "prove" D5 is a catalytically active helicase. It also provides a solid enzymological explanation for how poxviruses might initiate genome and lagging strand DNA replication.

C. Data and methodology - The conclusions are convincing and supported by reams of state-of-the-art experimental data.

D. Appropriate use... - The authors have taken care not to over interpret their data, informing the reader of situations where the models might fail due to uncertainties in the experimentally-derived structures.

E. Conclusions - The conclusions seem to be sound. Interesting and testable models are proposed.

F. Suggestions - The literature review covers most of the more critical literature relating to poxvirus DNA replication. From the wording, it might be implied that the rolling hairpin model is still the "prevailing model" for virus replication (lines 102-3). Realistically, the Moyer-Graves model is only of historical importance and has long fallen into disfavor for the reasons the authors cite. Missing from the introduction is the observation that the polymerase-encoded proofreading exonuclease also plays a critical role in catalyzing virus recombination (PMID: 19224992; PMID: 36015016). There is an incomplete sentence that mentions the virus ligase (line 111), in that regard there are several papers showing that the SSB (I3 in VACV) is another essential replication protein (e.g. PMID: 29093092).

The authors describe dimers of F8/A22/E4 heterotrimers as "inactive forms" (lines 126-7). Many replication forks incorporate a pair of DNA polymerases (sometimes different gene products), super assemblages that are collectively responsible for coincident leading and lagging strand synthesis. Is it conceivable that these are actually biologically relevant structures?

G. References - See (F) for a few comments. It has been claimed that poxviruses can recruit PCNA, and so presumably its associated factors, to the factories that are sites of DNA replication (PMID: 28467896). Despite various efforts this controversial observation has never been replicated. I'd argue that the work described in the current manuscript supports the

contention that, with few exceptions (cellular topoisomerase II), poxviruses do actually encode all the core machinery necessary to catalyze DNA replication. There is no need to posit the recruitment of host systems.

H Clarity - the manuscript is well written and the figures are nicely organized and well drawn. I did have some trouble orienting myself when inspecting the figures. Admittedly the structure figures are already quite complicated, but could the authors please always tab the 5'- and 3'-ends of the templates and also indicate the N- and/or C-termini of the DNA polymerase where possible? It would help orient the reader.

David Evans

Referee #5

(Remarks to the Author)
Yu et al. provide evidence that supports the hypothesis that the MPXV D5 helicase-primase RNA polymerase and helicase activities are stimulated by the presence of the viral DNA polymerase (F8-A22-D4). This work is of importance because it has been declared an international public health emergency by the WHO. The set of reported structures in this study contrasts differences between previously reported structures in the literature. They resolve the structure of the entire replisome bound to a DNA template using single-particle cryoEM. The structures reveal that the D5 RRM undergoes a significant conformational change in the presence of DNA polymerase, allowing access to the helicase domain. Furthermore, they also provide biochemical and single-molecule data confirming their hypothesis. Overall, the manuscript is well written and provides clear evidence to support the author's hypothesis. We would like the authors to consider a few points outlined below. This work may be suitable for the broad range of readers at Nature; however, several concerns need to be addressed prior to further editorial processing.

Mass photometry data (Figure 1c, extended Figure 1b):
The mass photometry data shows several peaks for the polymerase-only sample. It is unclear which peak corresponds to what because the authors don't state the expected weights for the individual subunits. Please add the expected weights for each subunit to the manuscript. Furthermore, the authors propose that the peak observed at 152 kDa corresponds to the fully assembled polymerase, despite their expectation of a peak at 192 kDa. This would correspond to an error of about 20%. Refeyn reports an expected measurement error of ~5%. How can you explain this discrepancy? Without knowing the expected size of all subunits, it is difficult to judge whether the 152 kDa peak is lacking a subunit. Additionally, a very small peak is observed at the expected size of ~200 kDa. Could this be the fully assembled polymerase? The peak that the authors suggested to be the polymerase dimer is also ~20% off from the expected size. Please clarify.

Mass photometry experiments are not consistent across figures. For example, in Figure 1c, the replisome is estimated to be 680 kDa, but in Extended Data Figure 1, the replisome is now 752 kDa, a 72 kDa difference that is quite significant given the sizes of the smaller components, such as F8/A22/E4. Could the authors rationalise what is causing this? SDS-PAGE of each component will show its purity and help identify any impurities that may cause this.

Helicase activity (Figure 3c, Extended Data Figure 5):
• Important controls are missing:
o Individual components for Fork DNA (e.g., blue and red ssDNA) – can you stain the gel to show the purity of the other strand?
o Assembled Fork DNA on its own (e.g., without D5 and polymerase)
• What are these intermediate bands that are present? Authors identify the topmost band as Fork DNA but as the reaction progresses, the intensity of the band doesn't visually decrease much. Further quantification of this experiment with properly identified bands and products will be required.
• Authors describe enhanced helicase activity when polymerase is at a 2:1 ratio with D5, given that structurally, it appears as a 1:1 complex. Do the authors have an explanation for this observation?

Single-molecule experiments (Figure 3d-h, Extended Data Figure 5-7):
A missed opportunity for the single-molecule experiment would have been to correlate fluorescence with the force measurements that are presented. Fluorescent labelling of D5 and polymerase would allow you to correlate complex formation with helicase activity. This would address several questions:
• Is the pausing seen (Ext Data 7) due to polymerase dissociation (loss of polymerase fluorescence)?
• How many polymerases are present (increased polymerase fluorescence intensity)?
• How likely is D5 alone to unwind (Ext Data 5 shows some traces that are active)? Is this actually D5 unwinding, or is it just the melting of the DNA junction?
• Control experiments of holding the DNA alone with no proteins present will clarify if it is spontaneously melting at such high forces.

Helicase activity has been previously reported at lower forces (~20 pN) – Burnham et al. (2019) Nat Comms [PMID: 31089141]. Could authors explore the force dependence for helicase activity?
• Are the higher forces presented in this study physiologically representative of MPX replication?
• Would you observe helicase activity at low forces (5 pN)?
• If you utilise a fork DNA substrate as the ensemble experiment, would you see helicase activity at low forces (5 pN)?

Line 216: "Unwinding experiments were conducted in a constant-force mode with a high-frequency feedback system adjusting the position of a bead to maintain the desired

force set point (usually 45–52 pN).” Can authors clarify why this range was used?

Line 223: “…no evident helicase activity was observed across all examined traces (n = 23)…” Examining the extended Figure 5, it appears that the helicase on its own exhibits some minor helicase activity. In my opinion, this is still in line with your overall
hypothesis and worth mentioning. I would replace “no evident helicase activity” with “minor helicase activity” or something similar. You could also elaborate on the limited processivity of that helicase activity (e.g., a maximum of 300 bp unwound).

In line 227: the statement “67 examined events from 29 traces” is unclear. What do the authors classify as an event? Are they separated by long pauses? And if yes, how long does the pause have to be for it to be a separate event?

We found the mechanism figure unclear:
• Re-order the figure in a more linear format because it is unclear where the mechanism starts at first glance
• d-f seems to be the mechanism, utilise a subtle box to indicate this
• g-h comparisons should be moved to the Extended Data Figure for clarity

Minor comments:
• Line 111: The sentence wasn’t finished.
• Fig. 3g: There is a typo: it should be 10 nM D5 and not 10nm.
• Line 781: You probably meant Pluronic F-127. (Pluronics F128 does not exist.)
• Across the manuscript, distances aren’t clearly defined. For example, page 8, lines 202-203 says: “The D5A RRM is displaced by 85 Å to interact with the F8 thumb, and the D5F RRM is 203 displaced by 70 Å to interact with A22.” – are these RMSDs? Clarify these instances across the manuscript.
• Figure 3c: clearer indication of FAM positioning on the substrate – e.g., FAM could be in green and a bit larger.
• Page 9, line 226: “we observed rapid DNA unwinding events at an average rate of 46 bp s-1” – Comparison to rates of other replicative helicases would be required to justify why the authors determine the MPXV helicase to be rapid.
• Ensemble and single-molecule experiments use concentrations across orders of magnitude. For example, in bulk, helicase activity requires μM concentrations, but in the single-molecule experiment, it can be observed at nM concentrations. Can authors justify this?

Referee #6

(Remarks to the Author)
(Per the editor’s request, I have focused my comments on the single-molecule experiments in this study.)

In this manuscript, Yu et al. presented cryo-EM structures of the DNA-bound MPXV replisome in pre-initiation and RNA elongation states. Compared to a previous structure of the helicase-primase (D5) in isolation, these new structures showed that D5 undergoes a large conformational change upon binding to the polymerase, exposing the ssDNA entry channel within the helicase. In addition, they performed bulk helicase assays and single-molecule optical tweezers experiments, both showing robust unwinding activities only with the helicase-polymerase complex but not with the helicase alone. These results nicely corroborate the structural data and thus are a valuable addition to the study. However, the single-molecule data need further analysis to be fully validated.

Major points:
(1) The unwinding traces were obtained at 52 pN of force. This is an unusually high force, almost reaching the overstretching regime of dsDNA. The authors need to provide evidence that the helicase-polymerase complex can unwind DNA at a lower force or explain why such a high force is required.
(2) Line 222: “In experiments containing MPXV D5 alone, no evident helicase activity was observed across all examined traces…” However, some traces in Extended Data Fig. 5 clearly exhibited limited unwinding. This is different from the polymerase alone which showed no activity (Extended Data Fig. 6). This distinction should be stated. Presumably these data were also collected at 52 pN of force. These constructs should be compared at a more physiological force.
(3) The example traces suggest that unwinding is not a uniform process, frequently interrupted by pauses (Extended Data Fig. 7). What is the origin of these pauses? Is the reported unwinding rate (46 bp/s) pause-free velocity?

Minor points:
(1) D5 without the primase domain is supposed to be active in unwinding. It would be valuable to conduct single-molecule experiments with this construct if it’s available.
(2) Line 225: “with both D5 and polymerase present, we observed rapid DNA unwinding events … with a markedly greater extent of DNA unwinding compared to D5 alone.” The maximum unwinding distance for these conditions should be quantified.

Version 2:

Reviewer comments:

Referee #2

(Remarks to the Author)
The authors have done an admirable job addressing my concerns regarding validating the structures with mutational studies. In addition, they have increased the rigor in their analysis, validating it with figure/densities. Statistical/validation analyses are also now complete. I recommend publishing and commend the authors on a much improved and impactful manuscript.

(Remarks on code availability)
I did not test the code for the C-trap analysis.

Referee #3

(Remarks to the Author)
I co-reviewed this manuscript with one of the reviewers who provided the listed reports.

(Remarks on code availability)

Referee #4

(Remarks to the Author)
The authors have addressed my earlier comments and concerns in a satisfactory manner. A description of any associated statistics has been provided in all of the figure legends.

(Remarks on code availability)

Referee #5

(Remarks to the Author)
The authors have done a great job addressing our concerns.

However, there are a few (minor) comments we would like to add.

Supplementary Table 1: For someone who isn’t familiar with the field, it would be helpful to also include the stoichiometry of the subunits and clarify the ratio of each subunit in the complex. This would help with help the reader understand how these masses were determined.

Figure 2: The abbreviations in the figure should be defined/included in the caption. E.g. is cont the DNA-only control?

(Remarks on code availability)

Referee #6

(Remarks to the Author)
The authors have done substantial work during the revision process, which significantly improved the manuscript. Technical difficulties associated with the single-molecule experiments, including fluorescent labeling without interfering with activity, use of a forked DNA substrate, and detecting activity at lower force in the presence of single-stranded DNA binding protein, left this part still somewhat unsatisfactory. Nonetheless, I still think it is a valuable complement to the structural work and together they represent a significant advance in the understanding of this important viral machinery. I do request two additional experiments that could further strengthen the manuscript:

(1) Compare the kinetics of DNA unwinding between 5' overhang and forked DNA substrates in the bulk helicase assay (Fig. 2b, c). Currently only the final normalized ssDNA levels are presented, which do not fully support the conclusion that DNA geometry does not affect the helicase activity.

(2) Given the higher activity of the E5dRRM construct, it should be tested further in the single-molecule assay to see if unwinding can be observed at lower forces.

(Remarks on code availability)

Referee #7

(Remarks to the Author)
I co-reviewed this manuscript with one of the reviewers who provided the listed reports.

(Remarks on code availability)

Version 3:

Reviewer comments:

Referee #6

(Remarks to the Author)
The authors have addressed my remaining concerns. I recommend publication of the revised manuscript.

(Remarks on code availability)

Open Access This Peer Review File is licensed under a Creative Commons Attribution 4.0 International License, which permits use, sharing, adaptation, distribution and reproduction in any medium or format, as long as you give appropriate credit to the original author(s) and the source, provide a link to the Creative Commons license, and indicate if changes were made.
In cases where reviewers are anonymous, credit should be given to 'Anonymous Referee' and the source.
The images or other third party material in this Peer Review File are included in the article's Creative Commons license, unless indicated otherwise in a credit line to the material. If material is not included in the article's Creative Commons license and your intended use is not permitted by statutory regulation or exceeds the permitted use, you will need to obtain permission directly from the copyright holder.
To view a copy of this license, visit https://creativecommons.org/licenses/by/4.0/

We thank the reviewers for their constructive criticism of the manuscript, and include our point-by-point responses below. Please note that we have changed the viral helicase name from “D5” to “E5.” While both D5 and E5 have been used in prior publications by other groups (and the prototype vaccinia virus homologous helicase is D5), we have chosen E5 to designate the mpox virus helicase gene product.

Referee #1 (Remarks to the Author)

This manuscript presents cryoEM structures of the MPXV replicative helicase (D5) and polymerase (trimeric F8/A22/E24) in complex with ssDNA or an RNA/DNA hybrid. Additionally, it examines the stimulation of helicase and primase activities of D5 by the presence of the polymerase. Recent studies have already reported the structures of the helicase and polymerase, as well as the auto-inhibition of the D5 helicase activity by its primase (RRM) and ZBM domains. The current manuscript introduces the following findings, (1) identifying the interface between helicase D5 (primarily through its RRM and ZBM domains) and the thumb domain of the polymerase F8 subunit, and (2) characterizing the activities of D5 and a low-resolution structure of RNA synthesis facilitated by the polymerase. While these findings complement the existing knowledge, the advances appear incremental, and inconsistencies alongside incomplete structural and functional data (as detailed in the comments below) suggest that the manuscript may not meet the standards for publication in Nature at this time.

1. The DNA molecule used in the structural determination of MPXV helicase-polymerase assembly is a hairpin duplex with a 5' overhang of 40 Ts (ED Fig. 1c), and not a forked DNA with both 5' and 3' overhang, as suggested in Fig. 2a.

We thank the reviewer for bringing up this important point. This comment prompted us to re-examine our prior cryo-EM maps. As the reviewer notes, the DNA substrate we used for structural analysis in the initial submission was a hairpin duplex DNA molecule with a 5′ overhang of 40 Ts (see Extended Data Fig. 1c, which is included below).

After closer re-examination of the maps at low contour before the post-processing step, it appears that the complex we visualized with the hairpin duplex DNA molecule with a 5′ overhang of 40 Ts is assembled on the ssDNA portion. In maps before post-processing, we can see density for the intervening ssDNA (see Extended Data Fig. 2b and below). This observation suggests that the density for the connecting ssDNA segment, which is likely flexible, was weakened during post-processing. Importantly, the connecting ssDNA segment is solvent-exposed, and there is sufficient space at this position to accommodate dsDNA as part of a replication fork.

To address this reviewer comment, we determined a new cryo-EM structure of the polymerase holoenzyme–helicase complex bound to a forked DNA substrate that contains both 5′ and 3′ overhangs (see Fig. 1f, Extended Data Fig. 5b,f, and below). The forked DNA-bound complex has an overall resolution of 6.5 Å, with local resolutions of 4.1 Å for the polymerase and 4.5 Å for the helicase. This is in comparison to the higher resolution structure of the ssDNA bound complex (overall resolution of 4.1 Å, with local resolutions of 3.5 Å for the polymerase and 3.8 Å for the helicase). Nonetheless, the maps of the DNA-fork bound complex allow clear visualization of the position of the DNA fork.

In the new map, we observe density for the duplex region of the forked DNA at the position we had inferred from the structure of the ssDNA-bound complex. The positioning and conformations of helicase and the polymerase are essentially identical to that seen in our original ssDNA bound structure.

These new data demonstrate that the MPXV replisomes assembled on a ssDNA segment or on a forked DNA substrate are near identical.

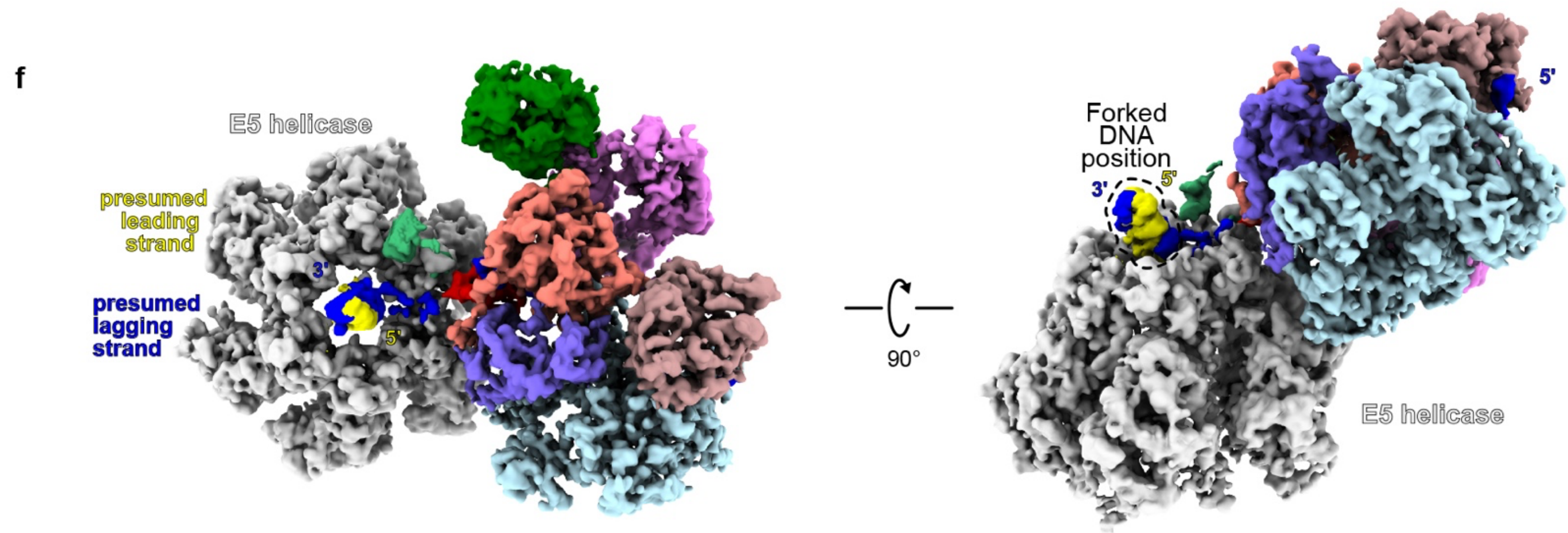

Fig. 1f. Composite cryo-EM map of the MPXV replisome bound to forked DNA. Overall map resolution is 6.5 Å; a composite map comprising maps focused on the helicase (4.5 Å) and primase-bound polymerase (4.1 Å) post-processed with DeepEMhancer[47] is shown. Polymerase subunits are colored as in **e**, and the E5 helicase domains are shown in gray.

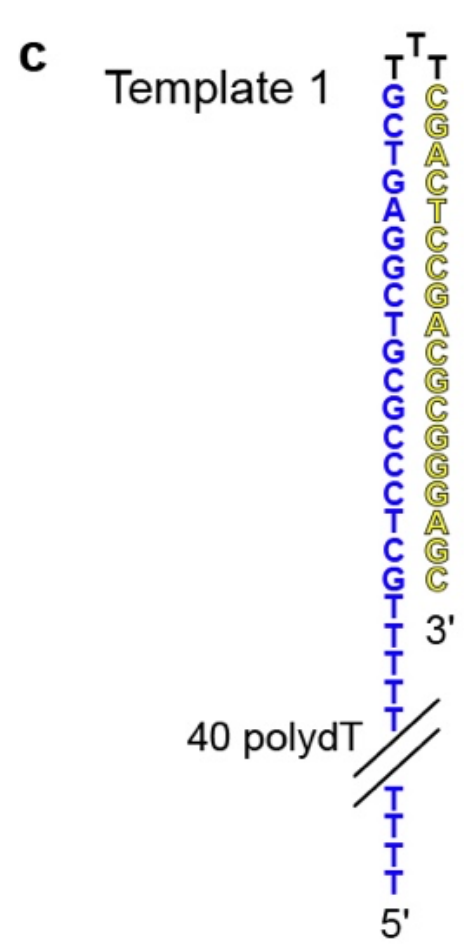

Extended Data Fig. 1c.
Schematic diagram of the hairpin DNA (template 1) used to determine the structure of the ssDNA-bound MPXV replisome.

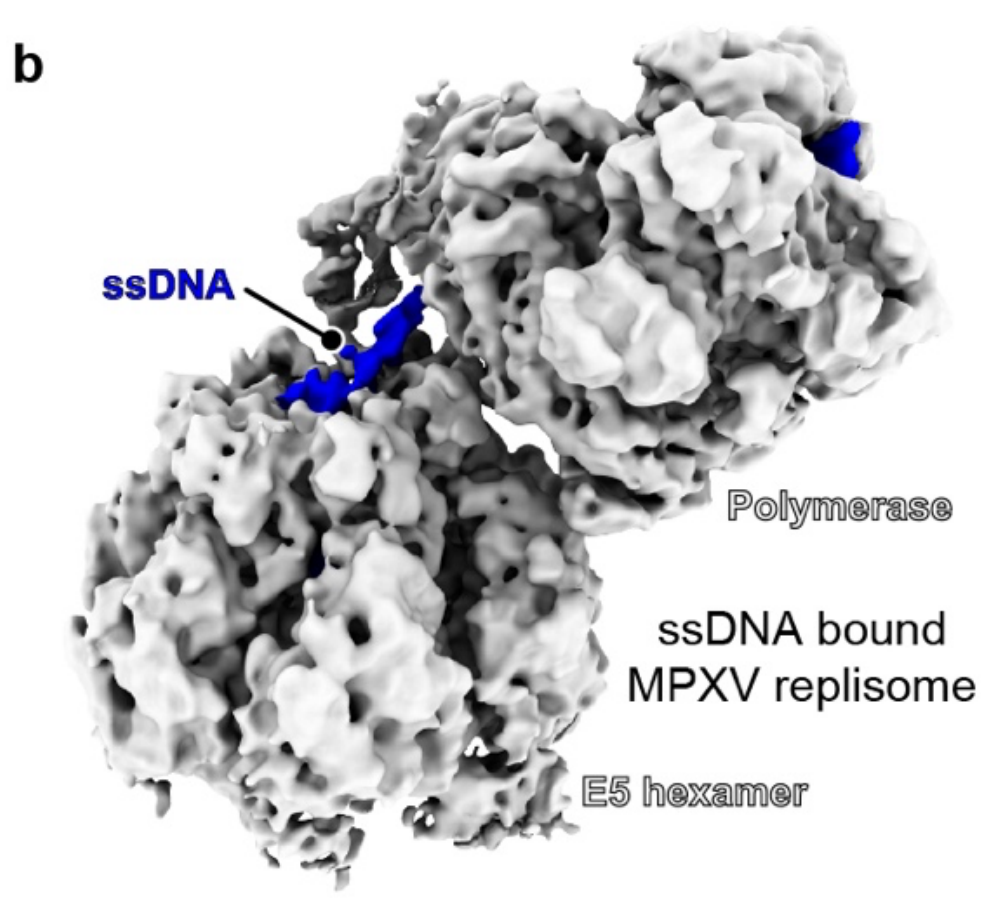

Extended Data Fig. 2b.

b, Cryo-EM map of the ssDNA-bound MPXV replisome, showing the location of ssDNA density bridging the helicase and polymerase, which can be observed at low map contour levels in the map before post-processing (overall map resolution 4.1 Å).

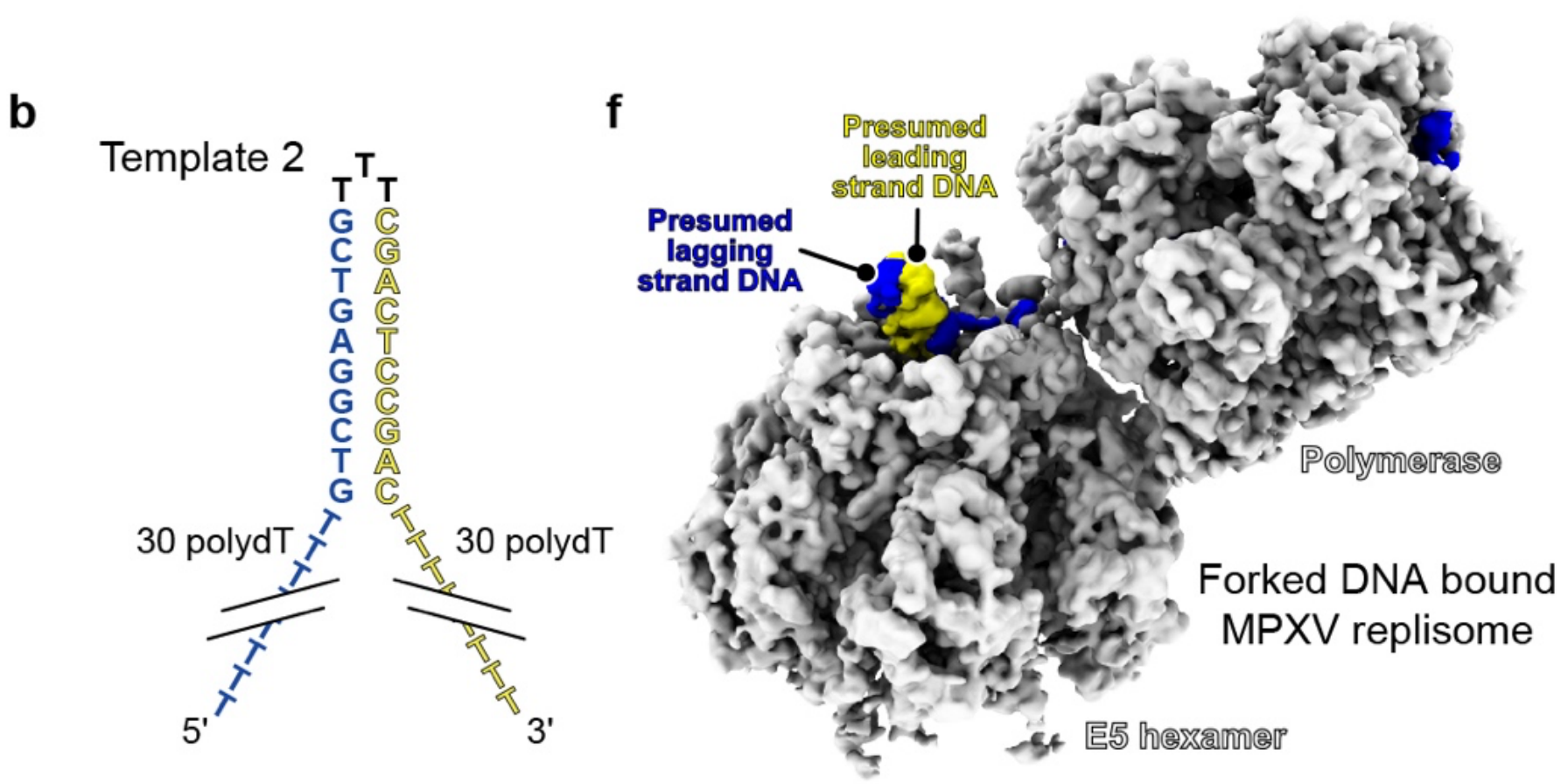

Extended Data Fig. 5b, f.

b, Schematic diagram of the forked hairpin DNA (template 2) used to determine the structure of the forked DNA-bound MPXV replisome.

f, Composite cryo-EM map of the forked DNA-bound MPXV replisome, post-processed with DeepEMhancer[47].

2. In the cryoEM structure, the observed DNA appears as two separate pieces of ssDNA without any duplex region. The segments, 10 and 17 nt in length, are associated with D5 and polymerase, respectively, but are not connected in the cryoEM volume. It is unclear if they originate from the same DNA molecule. What is the molecular rationale for interpreting them as the leading (bound by D5) and lagging strand (bound by polymerase) DNA? Where is the duplex DNA? Without a DNA fork, how can a replisome form, pre-initiation or initiating?

This important point is related to comment 1 above. As noted in our response to that comment, in the revised manuscript we describe how the initial cryo-EM structure was determined with the complex assembled on ssDNA that can be visualized as connecting the 10 and 17 nucleotide segments in the cryo-EM volume (features of this connecting segment are clearly visible in the map before post-processing).

Additionally, we have obtained a new cryo-EM structure with a polymerase–helicase complex assembled on a forked DNA substrate, which allows us to visualize the DNA fork (see Extended Data Fig. 5b). The DNA fork is positioned where the weak ssDNA bridge density was previously apparent.

The molecular rationale for interpreting the leading strand as bound by E5 and the lagging strand as bound by the polymerase is based on the known directionality of the helicase, which is a superfamily-3 (SF3) helicase that is expected to translocate in the 3′ to 5′ direction on DNA. This ssDNA binding mode and directionality for the helicase is also supported by a high-resolution structure of MPXV E5 bound to ssDNA (2.7 Å) (PMID: 38914567). In our working model, this assembly mode would allow the primase-bound polymerase to synthesize RNA primers on the presumed lagging strand (see Figs. 5e,f and below). We clarify in the revised manuscript our rationale for interpreting each strand as such in the working model. See lines 395–404.

We have also removed the terms “pre-initiation” and “initiating” from the manuscript as we agree that these terms were confusing. We instead describe in the revised manuscript the conformations that are observed.

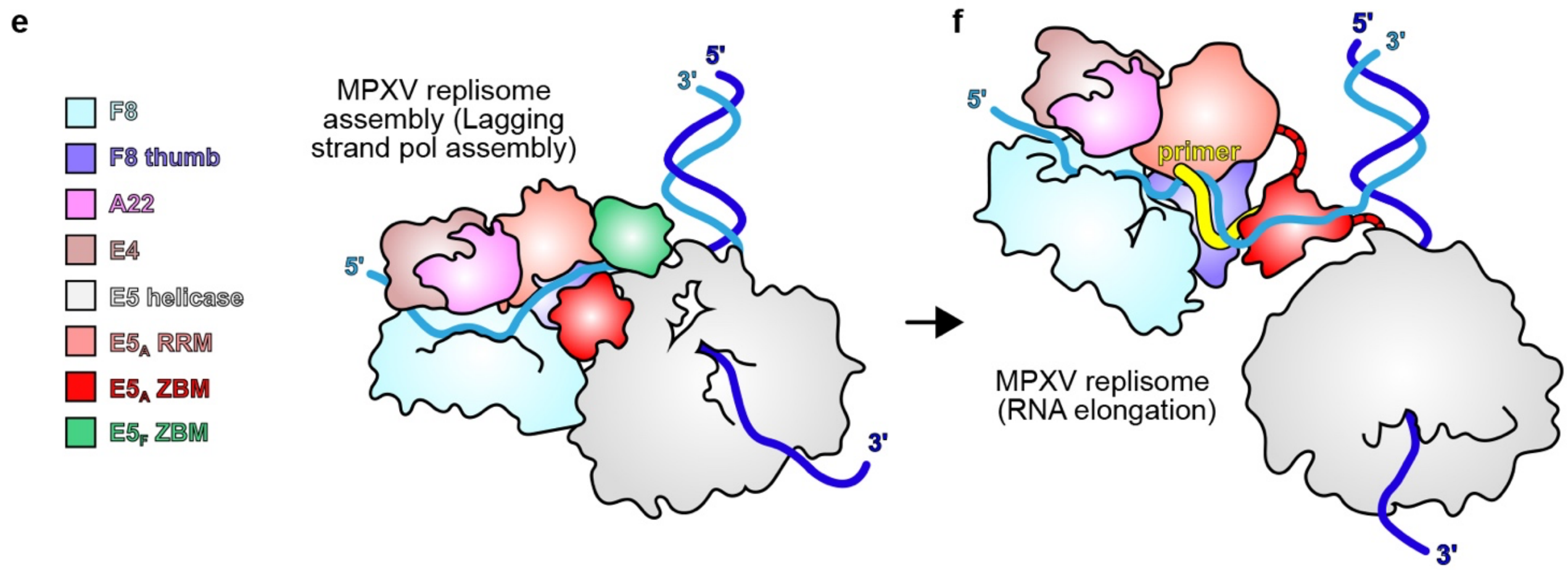

Fig. 5e, f.

e,**f**, Working model of MPXV E5 helicase–primase and polymerase loading (**e**) and DNA unwinding followed by RNA primer addition (**f**). The $E5_F$ RRM, which interacts with A22, is not shown as it would be in the front plane of the page.

3. Is there DNA polymerase activity on the DNA substrate used in the cryoEM study (the hairpin duplex with a 5' 40-T overhang) in the presence of D5? Do the primase and DNA polymerase compete for the DNA substrate?

We thank the reviewer for this interesting question. The hairpin-containing duplex DNA substrate with the 5′ 40-T overhang we used for cryo-EM structure determination has a free 3′ end that could be elongated by DNA polymerase. Based on the structure, we would indeed hypothesize that the E5 primase domain and DNA polymerase would compete for the DNA substrate–specifically, the E5 primase domain would bind the polymerase F8 thumb if added before dNTPs and would interfere with DNA synthesis. We used DNA elongation assays to test the hypothesis that addition of E5 would DNA elongation of the substrate. The results of these DNA elongation assays suggest that addition of E5 inhibits DNA polymerase elongation in the presence of dNTPs (see Extended Data Fig. 7g, h and below). Interestingly, in these experiments, we also included an E5 mutant in which the RRM domain (which includes the primase domain) is deleted ($E5^{\Delta RRM}$). $E5^{\Delta RRM}$ is not expected to be able to interact with the polymerase. Adding $E5^{\Delta RRM}$ allowed the polymerase to retain some DNA elongation activity, albeit reduced compared to the polymerase without any E5 added, likely because $E5^{\Delta RRM}$ would still interfere with DNA elongation by competing for the substrate.

Overall, these findings suggest that binding of the E5 RRM to the polymerase thumb interferes with DNA elongation in the presence of dNTPs, and that DNA polymerase and helicase for substrate binding in these assays. These new results are discussed in lines 361–365 of the revised manuscript.

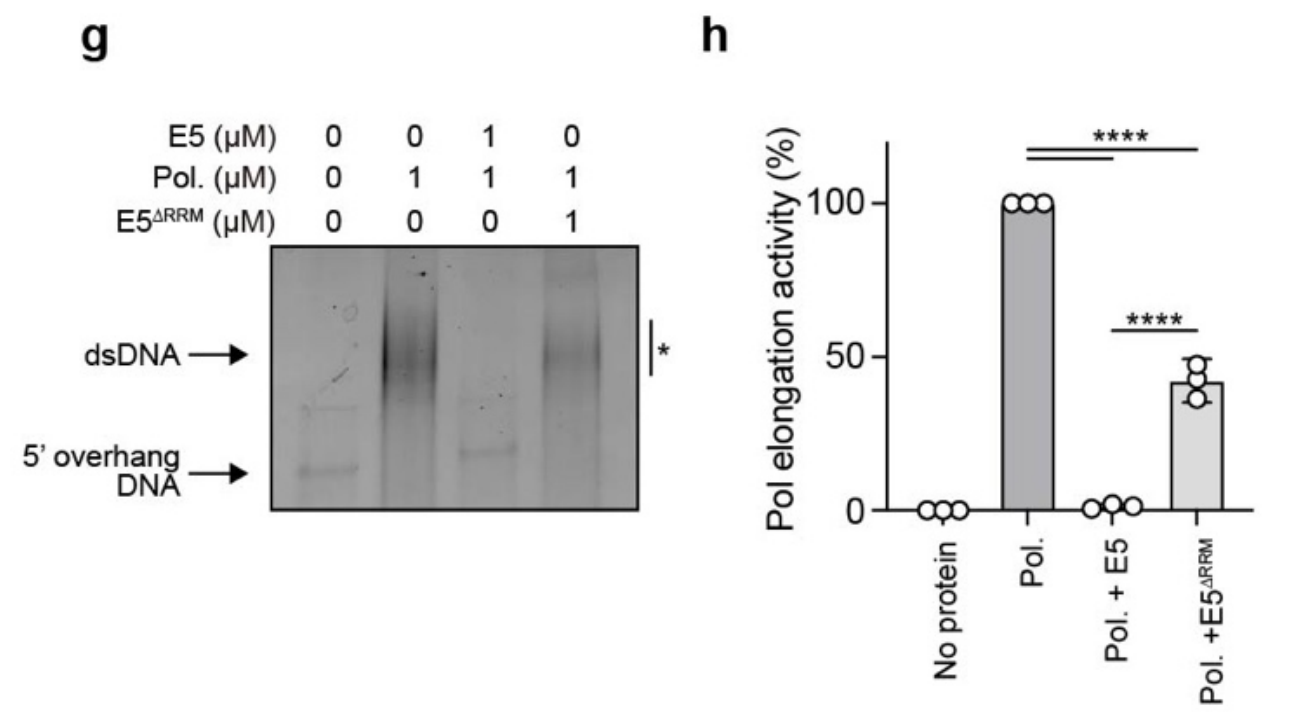

Extended Data Fig. 7g, h.

g,**h**, Polymerase DNA elongation assay performed with polymerase alone or co-incubated with wild type E5 or E5$^{\Delta RRM}$ using a DNA substrate containing a 5′ overhang (**g**). Quantification is shown (**h**). This experiment was performed three times (n = 3), and a representative gel is shown. One-way ANOVA with Tukey's multiple comparison test (****$P < 0.0001$).

4. The DNA used in the solution helicase activity assay is a forked DNA (Fig. 3c) with overhangs on both 5' and 3' ends. Why wasn't this DNA used in structural studies? Moreover, the DNA used in helicase activity assays by optical tweezer (Fig. 3d) has an overhang on either the 5' or 3' end, but not both. Previous studies on the D5 helicase activity suggest that it exhibits much higher activity on a forked DNA substrate than on substrates with either 5' or 3' overhang (Ref. #5). The use of different DNA substrates across various assays (structural studies and two helicase activity assays) complicates direct comparisons, akin to comparing apples and oranges.

We thank the reviewer for this important comment. The reviewer notes that we used DNA substrates that differ in their configuration in different assays, which limits the ability to compare results across assays. To address this concern, in the revised manuscript we provide a new cryo-EM structure and new sets of activity assays that allow easier comparisons across structural studies, biochemical assays, and optical tweezer experiments.

As noted in the response to comment #1, we also now include a structure of the complex bound to a DNA-forked substrate (see Fig. 1f and Extended Data Fig. 5b).

We have also performed a new set of activity assays with a substrate that contains only a 5′ overhang, so that it is similar to the DNA substrate we used for our cryo-EM structure of the ssDNA-bound complex included in the original submission (see Fig. 2b, which includes new data, as compared to Fig. 2c, which shows the prior data with the forked-DNA substrate). These new results demonstrate that E5 DNA unwinding activity is also activated by polymerase on a DNA substrate that contains a 5′ overhang, not just on a forked-DNA substrate.

In terms of the DNA substrate we used for optical tweezers experiments, we were limited by the commercial kit we used that are available for the LUMICKS C-trap instrument. The kit (LUMICKS Cat# SKU 00027) contains a biotin-labeled 17 kb DNA molecule with two nicks that are 5 kb apart. The ssDNA segment is removed by pulling on the DNA molecule. Although the substrate we used for the single-molecule assays does not contain both 5′ or 3′ overhangs, in the revised manuscript we used multiple substrates for in bulk biochemical assays and for structural studies. We also note that the single-molecule assays and in bulk assays include controls to allow internal comparisons for effects observed on the individual substrates.

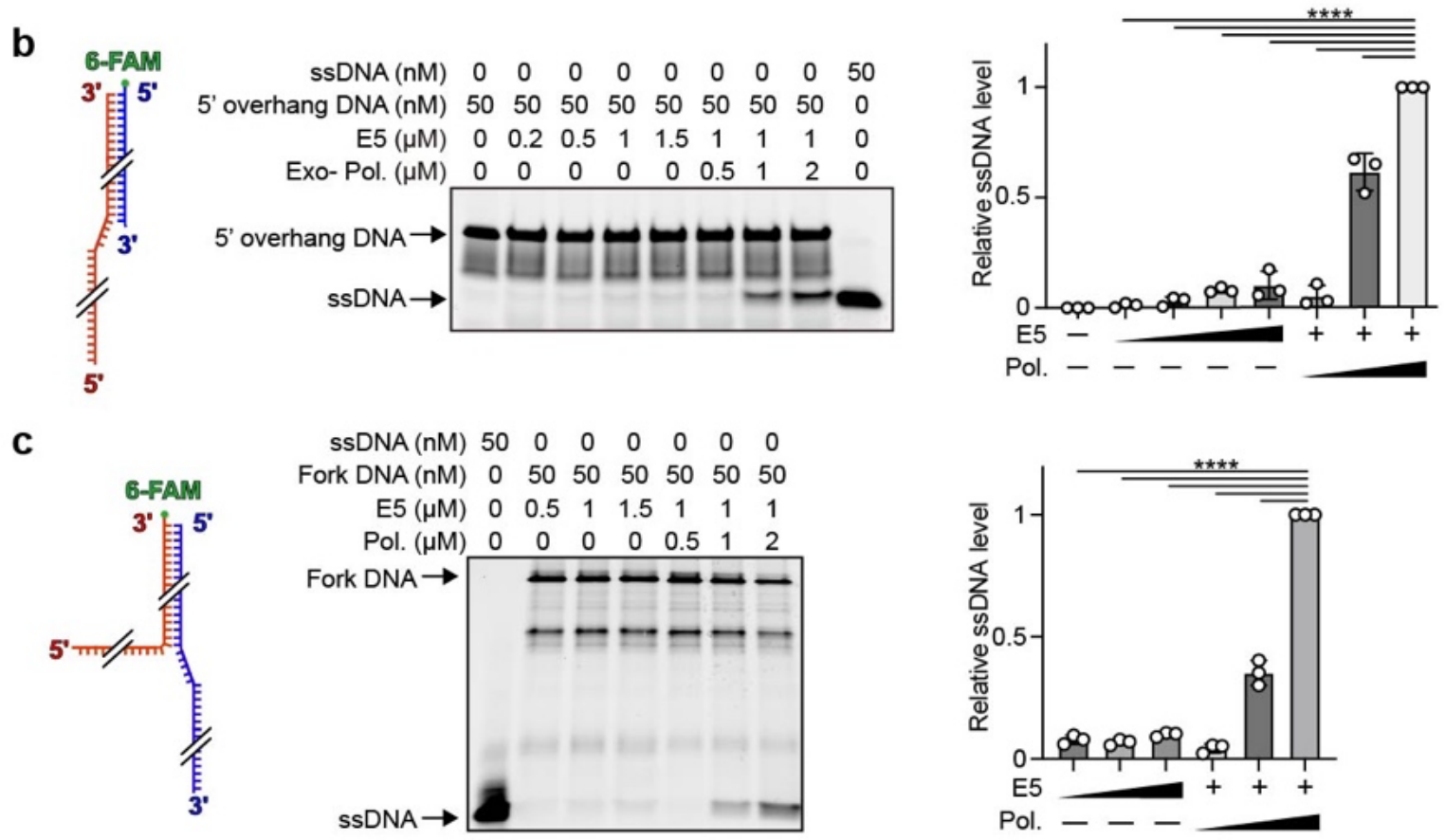

Figs. 2b, c

b, c, Helicase activity assay performed with MPXV E5 alone or co-incubated with polymerase using DNA substrates containing a 5′ overhang (**b**) or a forked-DNA substrate (**c**) visualized with a polyacrylamide-TBE gel. ssDNA control is the unannealed primer. Schematic diagrams of the DNA substrates and quantification of the ssDNA bands are shown. Data are mean ± s.d.; each experiment was performed three times (n = 3), and representative gels are shown. One-way ANOVA with Tukey's multiple comparison test (****P < 0.0001). 6-FAM, 6-carboxyfluorescein. Pol.: polymerase.

5. Previous studies have reported that deleting the primase (RRM) and ZBM domains from D5 removes the autoinhibition of D5 and activates its helicase activity (ref. #4, assayed at a 2:1 enzyme-to-DNA ratio). In the current study, helicase activity is only detectable at 20:1 enzyme-to-DNA ratio (Fig. 3c). Does removal of all six RRM and ZBM domains from the hexameric D5 activate the helicase activity more effectively than the addition of polymerase? How many RRM and ABM are required to block DNA substrate binding to D5? The authors should consider comparing the effects of RRM+ZBM deletion with that of polymerase addition.

The reviewer makes the important point that our activity assays were performed with 20:1 enzyme-to-DNA ratio while prior work with an E5 mutant that lacks the RRM domains (residues 238–785; E5$^{\Delta RRM}$) from former ref. #4 (PMID: 38177671, which is now ref. #3) required only a 2:1 enzyme-to-DNA ratio. Regarding the enzyme-to-DNA ratio, we believe the requirement for a 20:1 ratio in our study, versus the 2:1 ratio reported in that prior work, likely stems from inherent differences in protein specific activity following purification.

Prior structural analyses suggests that 2–3 RRM domains and two ZBM domains, based on the conformations captured in cryo-EM structures, may block DNA substrate binding to E5 (PMID: 38914567, PMID: 38177671).

We agree with the reviewer that including the E5$^{\Delta RRM}$ mutant in our assays could allow direct comparison of how removal of the six RRM domains activates the helicase as compared to the addition of the polymerase to full-length E5. We have performed this experiment and include new data in the manuscript, which show that E5$^{\Delta RRM}$ has more robust activity than full-length E5 in the presence of polymerase (see Extended Data Fig. 7a,b, and below).

In response to comments from other reviewers, we have also included E5$^{\Delta RRM}$ in the optical tweezer experiments. These experiments demonstrate that full-length E5 in the presence of polymerase appears to pause on DNA in a manner that depends on the concentration of polymerase, suggesting that the pauses are related to polymerase dissociation events (see Fig. 2j). E5$^{\Delta RRM}$, however, pauses less on DNA, which may explain why E5$^{\Delta RRM}$ has more robust activity in biochemical assays.

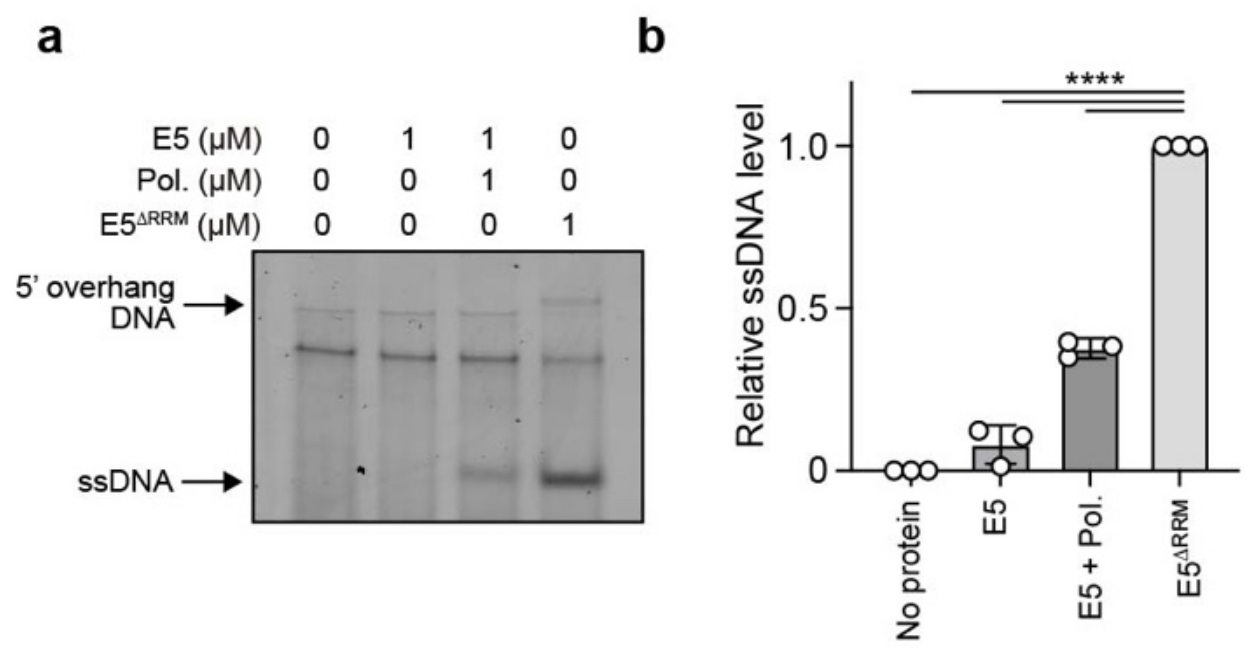

Extended Data Fig. 7a, b.

a,**b**, Helicase activity assay performed with MPXV E5 alone or co-incubated with polymerase or E5$^{\Delta RRM}$ alone using DNA substrate with a 5′ overhang (**a**). Quantification of the ssDNA, with mean ± s.d., is shown (**b**). This experiment was performed three times (n = 3), and a representative gel is shown. One-way ANOVA with Tukey's multiple comparison test (****P < 0.0001).

There are other issues that need attention:

(a) How are the terms "initiating replisome" (as mentioned in the title) and "pre-initiation state replisome" (as mentioned in the text) defined? These terms do not appear to be standard in the DNA replication field.

We agree with the reviewer that these non-standard terms should be removed for clarity. The revised title of the manuscript is "Structures and operating principles of an mpox virus replisome." The terms "initiating replisome" and "pre-initiation state replisome" have been removed from the manuscript.

(b) Why is the MP measured size of the polymerase trimer 152 kDa, rather than the expected 192 kDa (ED Fig. 1b)?

We believe the prior discrepancy (a measurement of 152 kDa ±10 kDa) were due to issues related to mass photometry instrument calibration during those experiments.

We have repeated the mass photometry experiments and the measured size of the polymerase holoenzyme heterotrimer in those experiments was 193 kDa, which is much closer to the expect 191 kDa.

We have provided the new data in Extended Data Fig. 1b. We also provide a summary or estimated and measured masses in Supplementary Table 1.

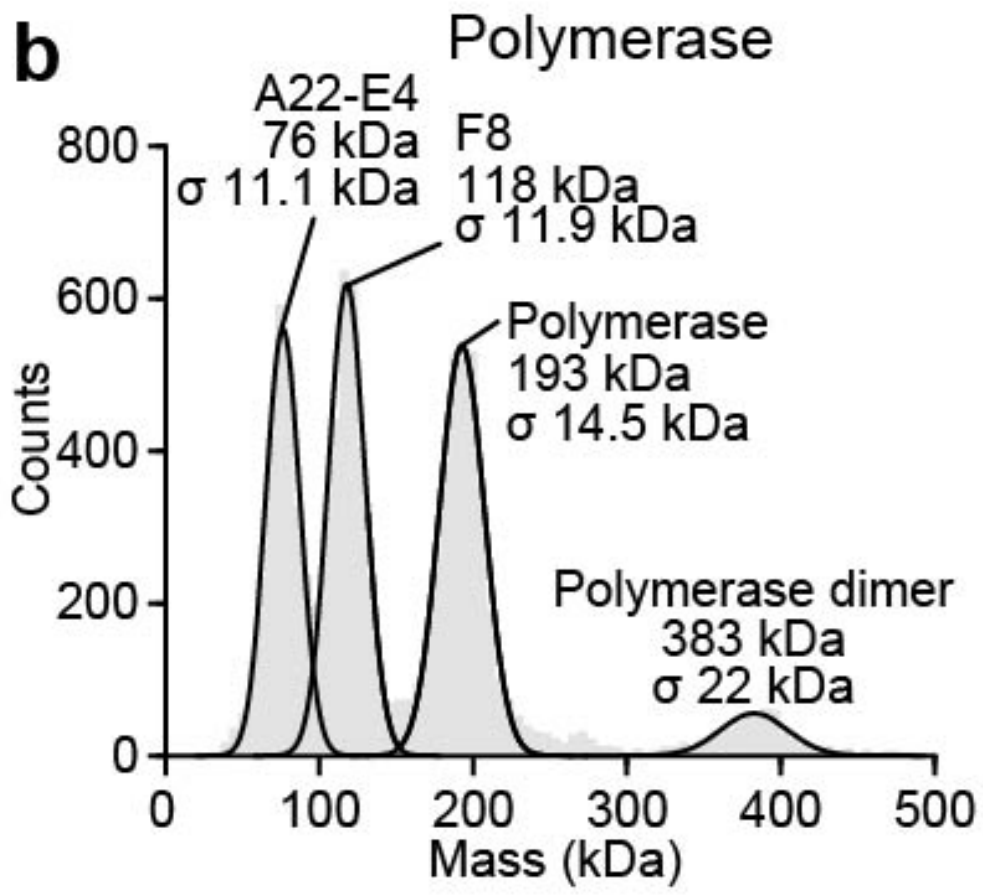

Extended Data Fig. 1b.

b, Mass photometry of the polymerase alone, E5 alone, or E5 and polymerase co-incubated without DNA. The experiment was performed twice (*n* = 2) and representative data are shown.

(c) What are the sizes of RNA synthesized by the primase activity (Fig. 4f)?

We have repeated the primase assays, and the new assays include a ladder, which reveals that the major products of the RNA synthesis step by E5 in our reactions are smaller than 15 nt (see Fig. 3f). The bands are, therefore, similar in size to those observed by De Silva et al. in their studies of the primase activity of vaccinia virus D5 (PMID: 18000036). Activity assays performed as part of that study observed a prominent radioactive band near the 14-nt phosphorylated marker, with additional material that migrates more rapidly.

Both the prior gels and the gel with the markers are included in the revised manuscript. We also note in lines 287–289 that the products we observed in our assays are similar to products observed in the De Silva et al. study with vaccinia virus D5.

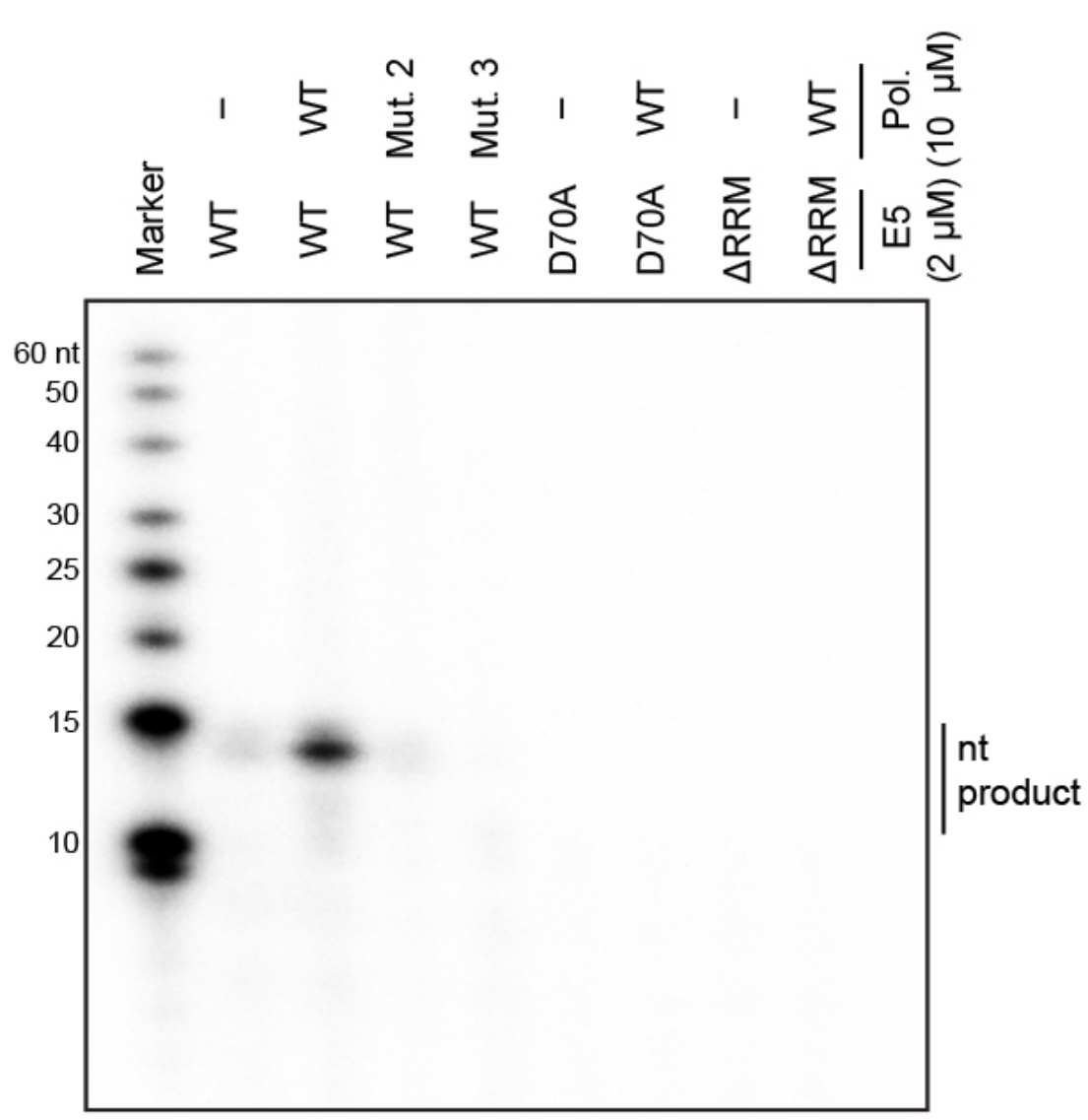

Fig. 3f (rightmost panel).

f, MPXV E5 primase RNA synthesis assay performed in the presence or absence of wild-type or mutant polymerases with M13 ssDNA and α-^{32}P-labeled CTP with radioactive products migrated on a urea-PAGE gel. Each experiment was performed twice (*n* = 2) with representative gels shown. nt: nucleotide; Mg: magnesium; WT: wild-type; Pol.: polymerase; Mut.: mutant.

(d) How might the two cryoEM structures reported in this study aid in the development of anti-poxviral drugs?

We agree with the reviewer that the manuscript should provide explicit information on how the cryo-EM structures reported in the manuscript could aid in the design of anti-poxviral drugs. This is also a concern that was raised by an additional reviewer. To address this concern, we have toned down our claims that the structure could aid in the rational design of inhibitors by removing this notion from the abstract and introduced the following text into the manuscript Discussion that describes the structures serving as starting points for virtual screens for small molecule inhibitors (lines 424–430).

"The conformations visualized could serve as a starting point for structure-based virtual screens for small-molecule inhibitors[42,43]. Inhibitors could be used to stabilize or lock conformational intermediates of the complex. This strategy could, at least in part, be analogous to the helicase–primase inhibitors against herpesviruses, another group of important human pathogenic DNA viruses. Those inhibitors bind at the interface of the helicase and primase domains and lock the viral helicase–primase in an inactive conformation to exert their antiviral activity[44-46]."

Referee #2 (Remarks to the Author):

Yu et al. use a combination of structural biology, bulk biochemical assays, and single-molecule assays to elucidate the configuration and mechanisms of the MPOX virus replisome. These experiments are important and well-executed, yielding a high-impact study. The authors demonstrate super-complex formation between the Helicase/Primase hexamer D5 and the viral DNA polymerase, which is required for D5 helicase activity. They further show that D5 primase activity is stimulated by polymerase binding. The authors also present a lower-resolution structure of the D5-pol complex that they claim is consistent with the priming/synthesis conformation. The homology to other primases and DNA polymerases, as well as the structural comparisons, is compelling.

Major comments: Mutational analysis is missing. While the difficulty of protein expression and purification is understood, only wild-type proteins are shown despite extensive modeling of protein-protein, protein-DNA, and active sites in the figures. At minimum, mutations (such as interface or active sites) that could test their models should be proposed. This would also allow the authors to address important mechanistic questions, such as whether polymerase binding is sufficient for helicase activity even in a polymerase active site mutant. The structural configurations observed, their similarity to the human replisome, and the activity-based assays are strong. However, given the low-resolution maps, if molecular details are emphasized, they must be supported by mutational data—otherwise, the interpretations should be toned down or the maps should be shown explicitly where interactions and active sites are discussed or modeled.

Small molecules that target large-scale conformational changes are notoriously poor immediate drug candidates. Given the conservation of active site residues and chemistry between viral and cellular replisomes, the drug development implications of this work are not strongly supported. However, this does not diminish the importance of the study or its suitability for Nature. If the authors wish to make claims about drug development, these should be supported with data, such as structural evaluation of molecules that inhibit helicase, primase, or polymerase activity or expanded discussion.

Order-of-addition experiments would further support the authors' structural models with biochemical evidence.

Points to address:

1. Can the authors clarify their argument/models regarding Okazaki fragment/discontinuous synthesis versus the rolling-hairpin model with a figure?

We thank the reviewer for this suggestion. While in the initial submission we presented the rolling-hairpin model as the prevailing model for poxvirus DNA replication, Reviewer #3 noted that the rolling-hairpin model has already been disfavored in the field (see Reviewer #4, comment A). We have thus removed the language in the text regarding this older model and instead discuss the RNA-primed DNA replication model as the prevailing one.

2. Can the authors provide examples of other antiviral development efforts using similar approaches? While they argue that their study will aid drug development, this discussion should be expanded beyond general commentary on large-scale conformational changes and protein-protein interactions.

This concern was also raised by another reviewer. We agree that identifying small molecules that target large-scale conformations may be a poor immediate drug target. To address this concern, we have removed from the abstract the drug-design implications of the work and expanded the Discussion to include more general information on how the structural studies could aid in anti-poxvirus drug development as part of future research directions.

The revised manuscript section is included below (lines 424–430).

"The conformations visualized could serve as a starting point for structure-based virtual screens for small-molecule inhibitors[42,43]. Inhibitors could be used to stabilize or lock conformational intermediates of the complex. This strategy could, at least in part, be analogous to the helicase–primase inhibitors against herpesviruses, another group of important human pathogenic DNA viruses. Those inhibitors bind at the interface of the helicase and primase domains and lock the viral helicase–primase in an inactive conformation to exert their antiviral activity[44-46]."

3. The authors should state that future mutational analysis is required to support the presented structures. Alternatively, they should conduct such analyses or focus the manuscript on general configuration and biochemistry rather than specific molecular interactions.

We thank the reviewer for this important comment. In the revised manuscript, we have conducted mutational analysis that focuses on evaluating the contribution of contacts between the helicase–primase and the polymerase observed in the cryo-EM structures. The mutations we studied include: (1) Mutations of A22 residues (F263 and F217) that interact with hydrophobic residues on the $E5_F$ RRM (see Extended Data Fig. 3g) and (2) deletion of an F8 thumb C-terminal region (residues 985–1006) that interacts with the $E5_A$ ZBM and RRM (see Extended Data Fig. 3e,f).

We generated recombinant polymerase holoenzyme complexes that contain mutant A22 or F8 subunits for use in functional assays (see Extended Fig. 7c). We then used the recombinant proteins in mass photometry experiments, helicase activity assays, and primase activity assays. In mass photometry experiments involving co-incubation with E5, polymerase mutants 1, 2, and 3, as compared to wild-type polymerase, did not form larger peaks consistent in size with replisomes (see Extended Fig. 7d and below). We also observed reductions in polymerase-mediated helicase activation in helicase assays (see Extended Fig. 7e,f and below). Additionally, polymerase mutants 2 and 3 were unable to enhance primase activity when co-incubated with E5 (see Fig. 3f and below).

We believe this new mutational analysis strongly supports the functional relevance of the interfaces visualized in the cryo-EM structures as they relate to MPXV replisome assembly, helicase activation, and primase activation.

c

Polymerase mutant	Mutation	Predicted effect based on structure
Mutant 1	A22 F217A + F263A	Disrupt interface between A22 and $E5_F$ RRM
Mutant 2	F8 C-terminal deletion (985–1006)	Disrupt interface between F8 thumb and $E5_A$ ZBM and RRM
Mutant 3	A22 F217A + F263A, F8 Cter deletion (985–1006)	Disrupt interfaces of A22-$E5_F$ RRM and F8 thumb-$E5_A$ ZBM

Extended Data Fig. 7c

c, Summary of the mutations designed to disrupt specific interfaces between the polymerase and E5. See also Extended Data Fig. 2e–g.

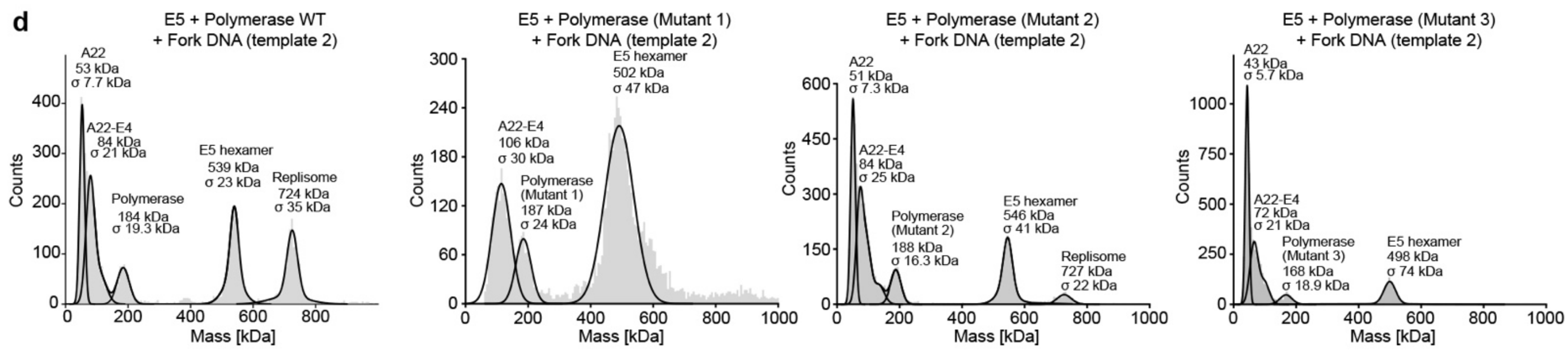

Extended Data Fig. 7d.

d, Mass photometry results of wild type MPXV replisome and MPXV replisome with mutant 1, 2 or 3. This experiment was performed twice (n = 2), with representative data shown.

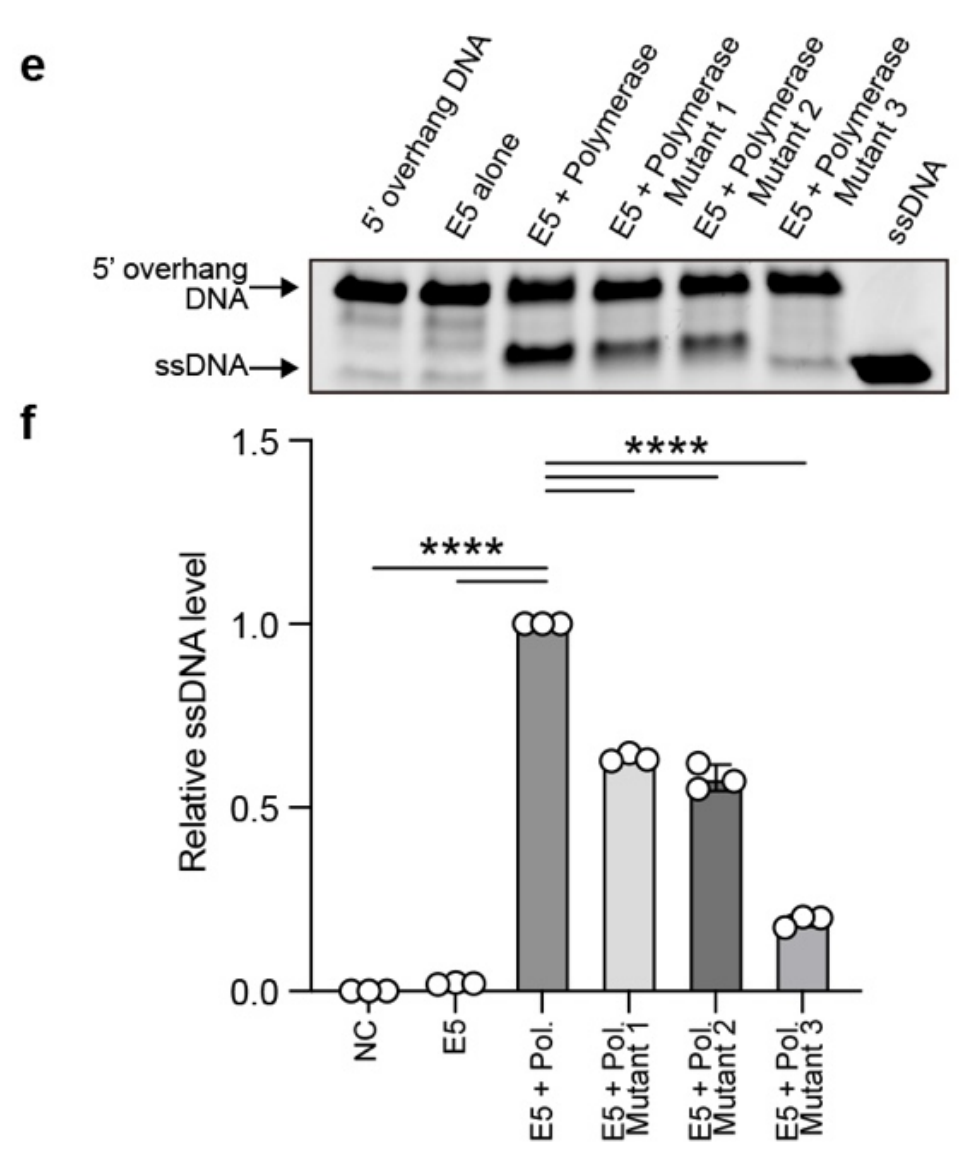

Extended Data Fig. 7e,f.

e,**f**, Helicase activity assay performed with MPXV E5 alone or co-incubated with wild-type or mutant polymerases using DNA substrate with a 5′ overhang (**e**). Quantification of the ssDNA band, with mean ± s.d., is shown (**f**). This experiment was performed three times (n = 3), and a representative gel is shown. One-way ANOVA with Tukey's multiple comparison test (****$P < 0.0001$).

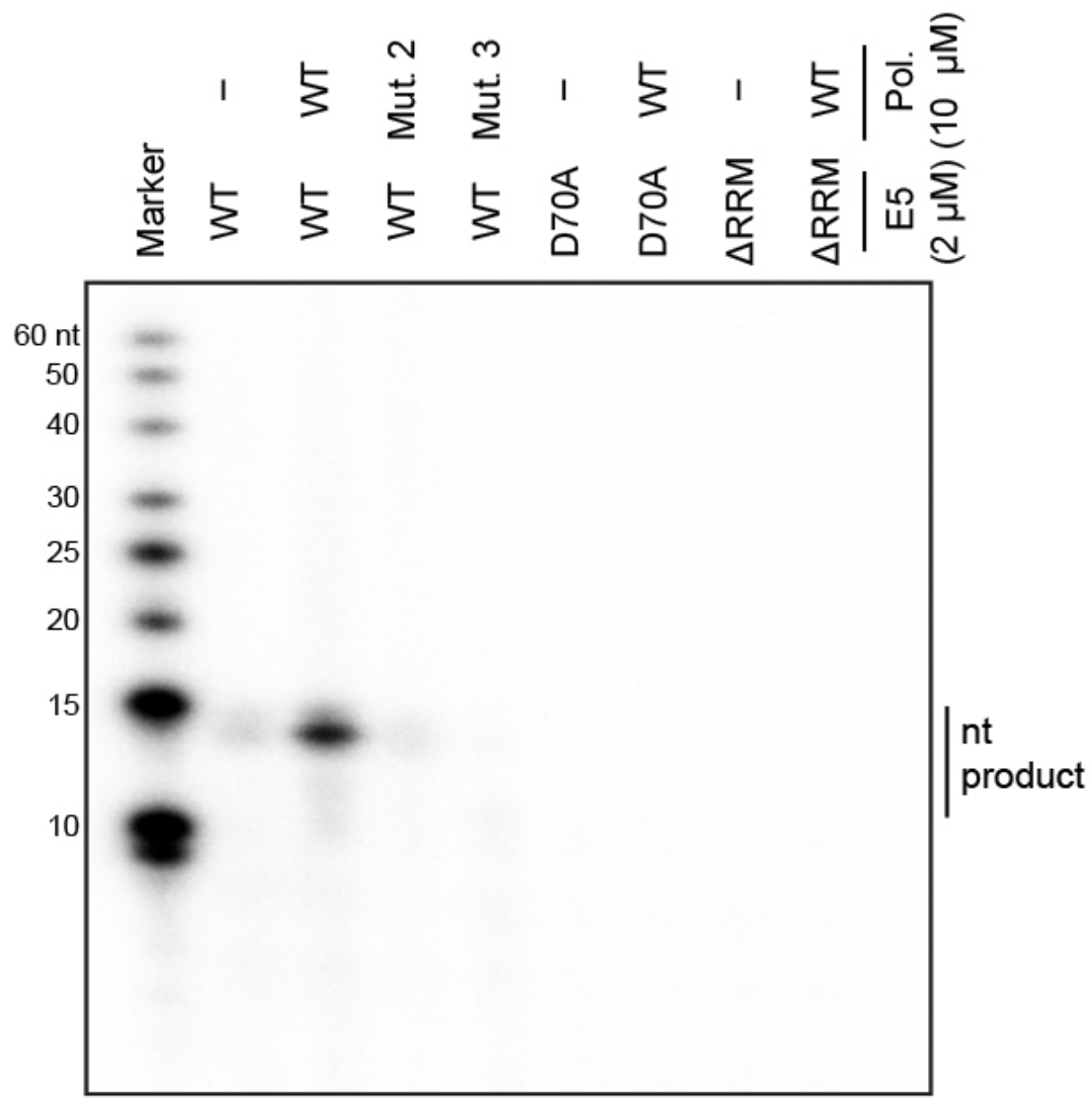

Fig. 3f.

f, MPXV E5 primase RNA synthesis assay performed in the presence or absence of wild-type or mutant polymerases with M13 ssDNA and α-^{32}P-labeled CTP with radioactive products migrated on a urea-PAGE gel. Each experiment was performed twice (*n* = 2) with representative gels shown. nt: nucleotide; Mg: magnesium; WT: wild-type; Pol.: polymerase; Mut.: mutant.

4. When discussing amino acid interactions (e.g., Fig. 2b–h and Extended Data 8e), the authors should show the corresponding densities to compare with the AlphaFold3 model.

In the revised manuscript, we have added new figure panels that show the corresponding cryo-EM density overlaid with the amino acid interactions (see Extended Data Fig. 3d,g and below). We have also removed certain interactions that were shown in the original manuscript in cases where the cryo-EM density was too poor to confidently visualize interactions. The density was poor for the interactions between $E5_F$ RRM and A22, but we also included density for this region for the readers to have easy access to this information (see Extended Data Fig. 3g and below)

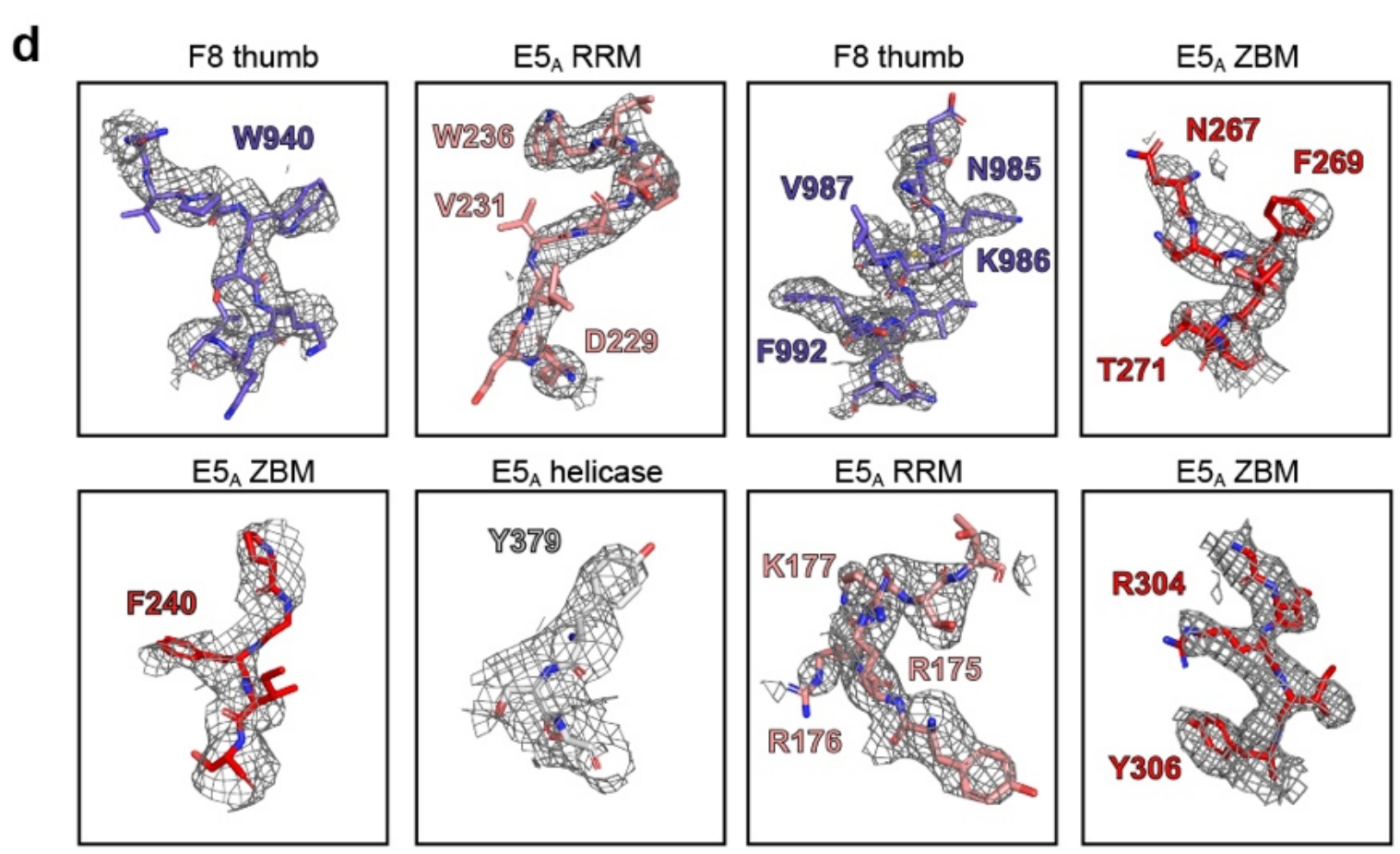

Extended Data Fig. 3d.

Cryo-EM density of the residues involved in polymerase and primase domain interactions during MPXV replisome assembly from an E5 primase and polymerase masked (3.5 Å) map post-processed with DeepEMhancer[47]. Residues are shown as sticks.

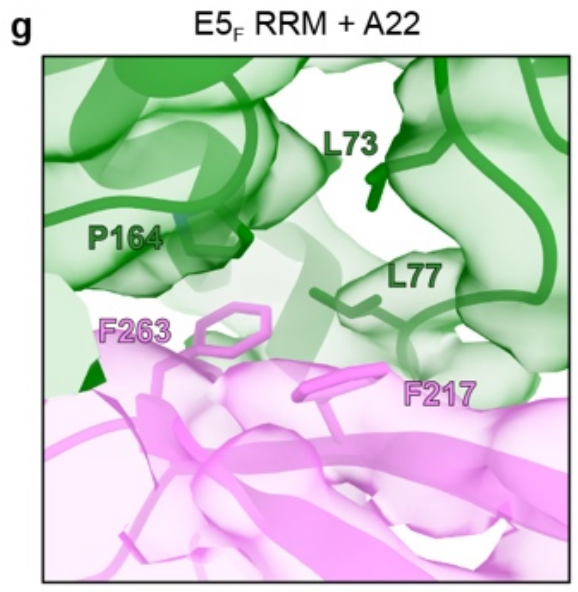

Extended Data Fig. 3g.
Interactions between $E5_F$ RRM and polymerase A22 subunit.

5. Move Extended Data Figures 9 and 10 to the main figures, and relocate Fig. 2b, c, d, e, g, h to the supplement. The structural alignments in Extended Data 10, showing comparisons between viral and cellular replisomes, are of broader interest than Figure 2. Similarly, Extended Data 9 is more relevant for a general audience.

We appreciate the reviewer's thoughtful suggestions regarding the organization of the figures in the manuscript. We agree that the structural comparisons between viral and cellular replisomes, as well as the broader functional implications shown in the Extended Data figures, are more relevant for a general audience.

In the revised manuscript, we have moved panels that were originally presented in Extended Data Figures 9 and 10 into the main text Figures 4 and 5. These panels include modeling of RNA-to-DNA elongation transitions based on the new cryo-EM structures and previously available structures (now Figs. 4d–g), and comparisons between the MPXV and cellular replisomes (now Figs. 5a–d).

We have also moved the detailed structural views that were previously presented in Figure 2b, c, d, e, g, and h to Extended Data Fig. 3.

6. Figures 1 and 5 (purification, complex formation, and overall cryo-EM structure): include reported resolutions.

We have added the overall resolutions to the figure legends for any figure in which a cryo-EM map is presented. Please note that the relevant figure legends in the revised manuscript are Fig. 1d, f, Fig. 4a, Extended Fig. 11a, 11e.

7. Figure 2 (protein-protein and protein-DNA interactions): consider showing orientations -and a zoom out figure of the entire complex- between panels more clearly, as it's difficult to understand the spatial relationships. Most of this figure could be moved to the supplement. In contrast, Extended data Figure S9e–j is essential to the authors' argument and should be in the main figures.

As noted above, we have reorganized the manuscript so that most of the panels that were previously presented as Fig. S9g–j are now presented in the main text Fig. 4d–g. We have moved Fig. 2 to the Extended Data, and it is now Extended Data Fig. 3.

To improve clarity and part of the revisions we have also merged two panels (content from Fig. 2g and 2h is now part of Extended Data Fig. 3c) and removed a panel due to poor cryo-EM density (that is, the previous Fig. 2c).

8. Figure 4: Biochemistry demonstrates RNA priming activity of D5, requiring Mg^{2+}, DNA, Pol, and helicase. The product is confirmed as RNA (not DNA) based on RNase vs. DNase sensitivity. However, the authors align D5 with PrimPol but do not mutate key residues—mutational analysis of predicted active site residues should be performed to validate RNA production. At the very least, note that such analysis is required.

To address this reviewer comment, we have performed primase activity assays including a mutant in which a key active site aspartate residue (D70) that would interact with metal in the active site is mutated to alanine (D70A, Fig. 3c). The E5 D70A mutant cannot synthesize RNA in the assays (Fig. 3f).

We also note that this residue had been mutated in a study examining the active site of MPXV E5 using in RNA synthesis assays (PMID: 38914567). That study had examined E5 residues D70, D72, D170, H134, R181, and K187, and found them to be important for RNA synthesis, which is also consistent with the AF3 model presented in Fig. 3c. A study on vaccinia virus E5 also showed that residues D70, D72, and D170 are required for DNA replication in cells (PMID: 18000036). These prior findings are now summarized in a Supplementary Table 2.

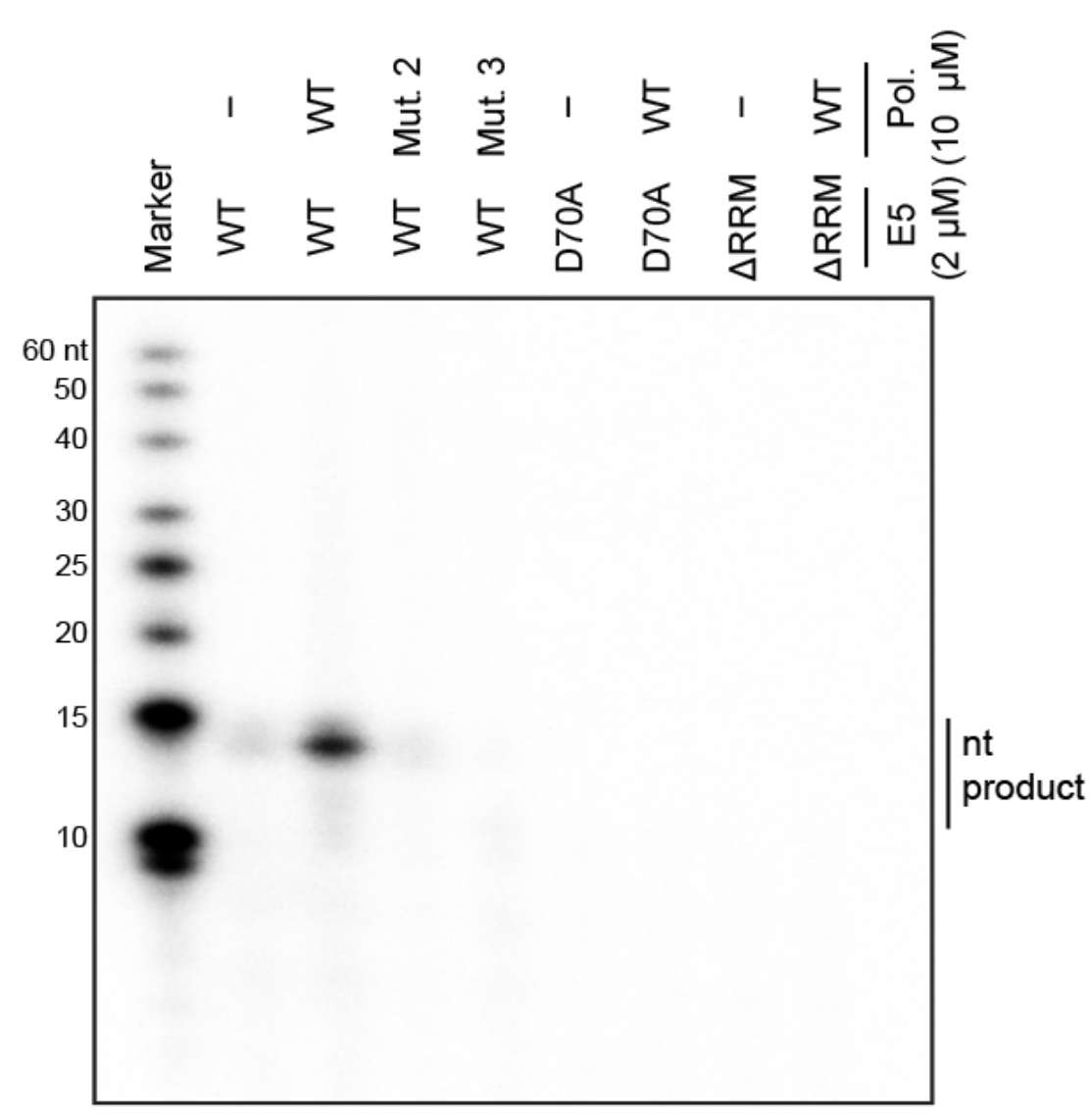

Fig. 3f
f, MPXV E5 primase RNA synthesis assay performed in the presence or absence of wild-type or mutant polymerases with M13 ssDNA and α-^{32}P-labeled CTP with radioactive products migrated on a urea-PAGE gel. Each experiment was performed twice (*n* = 2) with representative gels shown. nt: nucleotide; Mg: magnesium; WT: wild-type; Pol.: polymerase; Mut.: mutant.

9. Figure 5: Structural model of the MPXV replisome. This is a filtered lower-resolution map intended to resolve the DNA/RNA hairpin and is distinct from the structure in Figure 1. Clarify this in the figure legend and provide the resolution.

The panel originally presented in Fig. 5a was a ribbon diagram derived from a 5.6 Å cryo-EM structure of a subcomplex containing the polymerase and two primase subdomains (the RRM domains of $E5_A$ and $E5_F$). During revision of the manuscript, we obtained a higher-resolution map of this subcomplex and now present a 3.8 Å map of the complex. This new map and its associated ribbon diagram are presented in Fig. 4a and

Extended Data Fig. 11e. The associated cryo-EM workflow is provided in Extended Data Fig. 11b and 11c. The legend for Fig. 4a also includes the resolution of this map.

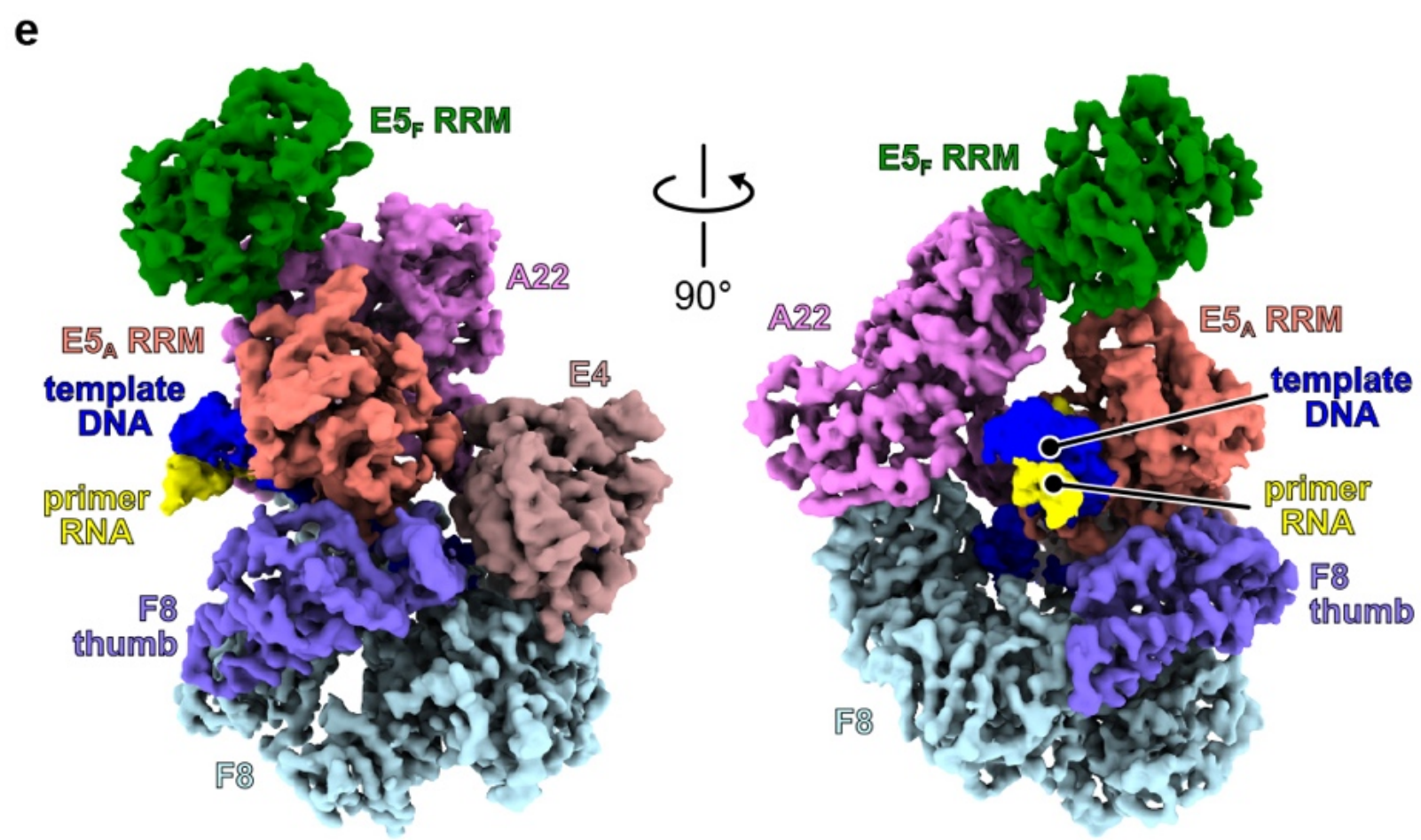

Extended Data Fig. 11e.

e, Two views of the 3.8 Å cryo-EM map of polymerase–primase core complex bound to RNA-DNA hybrid.

10. Can the authors show whether binding is separable from helicase activity? For example, can D5 or Pol bind the substrate without catalysis? Given that proteins and substrates are in hand, these experiments should be feasible.

The mass photometry experiments in Fig. 1c involved co-incubating polymerase holoenzyme (F8/E4/A22) and E5 with DNA in the absence of ATP, which would be required for catalysis. Under those conditions, we observed formation of the replisome complex, even though we would not expect the complex to be enzymatically active. Additionally, all cryo-EM structures were determined in the absence of ATP. Together, these data suggest that DNA binding is separable from helicase activity.

11. Additional biochemical assays would strengthen the manuscript, especially for Figures 3 and 5. Gel shift/DNA binding experiments and order-of-addition assays would help establish whether distinct replication states exist. For example:

o Use rNTPs alone to allow RNA synthesis without dNTPs

o Supply only 3 of the 4 required rNTPs to trap intermediates

o Include crosslinking or mutational trapping strategies These assays are critical given the low resolution of the structures and their central role in the authors' argument about helicase, priming, and synthesis transitions.

We thank the reviewer for this important comment regarding strengthening the manuscript through additional biochemical assays and for providing specific examples of how this could be accomplished.

The originally presented Fig. 3 showed how MPXV polymerase binding influences helicase activity (this is now Fig. 2), while Fig. 5 focused on presenting the structure of an MPXV polymerase and E5 primase subcomplex bound to a RNA-DNA hybrid (this is now Fig. 4).

The experiment presented in Fig. 3f includes rNTPs to allow for RNA synthesis in the absence of dNTPs. These assays show that RNA is synthesized in the presence of magnesium and that adding polymerase

increases RNA synthesis in a polymerase dose-dependent manner. Furthermore, Fig. 3f also includes new data with polymerase mutants 2 and 3, which contain mutations that disrupt polymerase association with E5 (see Extended Data Fig. 7c); these mutants were found not to enhance primase activity. In these new biochemical assays, we also show that E5 primase mutants in which an active-site aspartate residue is altered (E5 D70A) or the RRM is deleted are not active.

We agree that trapping intermediates by including 3 of the 4 rNTPs would be a very interesting set of experiments (and would likely also offer opportunities to trap intermediates for structural characterization). However, after discussion with the co-authors, we decided not to pursue these experiments as part of this study and plan to pursue them in future work aimed at structurally characterizing intermediates.

The reviewer comment about using cross-linking to support the visualized structures was related to the low resolution of the previous structure that was presented (5.6 Å). We have since replaced the lower resolution structures with a higher resolution structure of the polymerase–primase core complex bound to RNA-DNA hybrid (3.8 Å). In light of this higher resolution structure and mutational analysis of interface residues and RNA synthesis assays noted above, we have not pursued cross-linking studies.

12. The authors claim their structures support Okazaki fragment formation, in contrast to the prevailing rolling-hairpin model that lacks lagging strand synthesis. However, Figure 5 could be better supported with figures from Extended data 9 and especially 10 directly to the models of Okazaki fragment formation. Lines 193–197: These residues are modeled based on related structures, and if the goal is to revise the replication model, mutational support is essential or at least more thorough discussion and supportive figures.

Based on a comment from Reviewer #4 that the rolling-hairpin model has already been discounted, we have removed from the manuscript mention of the rolling-hairpin model as a prevailing model (see Reviewer #4, point F). Main text Fig. 5 in the original manuscript (Fig. 4 in the revised manuscript) now includes panels d–g, which were originally in Extended Data Fig. 9. Panels from the original Extended Data Fig. 10 (comparison of the MPXV and human replisome) are now included in main text Fig. 5 in the revised manuscript.
In the original manuscript, lines 193–197 previously read as follows:

"Five basic residues on the D5A RRM (K151, R152, R175, R176, and K177) contact the ssDNA phosphate backbone and one F8 thumb residue (R1000) contacts an ssDNA base (T4) (Fig. 2g). D5A ZBM residues R304 and Y306 interact with ssDNA on the face that is opposite the one contacted by the RRM (Fig. 2h). Examination of sequence alignments of poxvirus D5 primase domains reveals that residues that contact ssDNA are highly conserved (Extended Data Fig. 3)."

These residues were modeled based on cryo-EM density. A panel in Extended Data Fig. 3d in the revised manuscript now shows this cryo-EM density. For a residue for which cryo-EM density for the side chain was too weak to model based on density (R152), this residue was removed and the relevant figure and text adjusted accordingly.

We hope that these revisions, including removal of the rolling-hairpin model from the manuscript and the addition of supportive figures, address this reviewer's concern.

13. Mutations in D5A that block polymerase binding would provide biochemical support for the structural model. Such mutations should impair both polymerase binding and helicase activity (as shown in Figure 3). Discuss what mutations could be tested or do the authors have such data?

We agree with the reviewer that this is an important point. We performed mutational analysis to provide biochemical support for the structural models (as noted above; please also see the response to comment #3). We found that mutations that block polymerase binding to E5 prevent helicase activation (see Extended Data

Fig. 7e,f). We also tested these for replisome assembly in mass photometry experiments and also tested them for primase activity enhancement in primase activity assays (please see Fig. 3f and Extended Data Fig. 7d).

14. Calculate and report 3D-FSCs for all structures.

We have calculated the 3D-FSCs for all reconstructed structures to assess directional resolution and map anisotropy. These plots have been added to the updated Extended Data Figs. 1, 5, and 11.

15. Include a figure in the Introduction comparing the rolling-hairpin model and the lagging-strand/Okazaki fragment model.

As noted above in our response to the first comment above, we have removed discussion of the rolling-hairpin model based on a comment from Reviewer #3.

Minor notes:

• In the abstract, clarify that Mpox is a double-stranded DNA virus.

We have modified the abstract and the abstract now includes the following. "Poxviruses are double-stranded DNA (dsDNA) viruses with large genomes."

• **Extended Data 1b: Some bands are unlabeled. Add a table showing expected vs. observed masses, and note that discrepancies fall within error.**

We thank the reviewer for raising this issue. We have now labeled all peaks in the results of mass photometry experiments. We have also included a supplementary table that shows the expected versus observed masses in mass photometry experiments (see Supplementary Table 1).

• Extended Data 1c: It's unclear which panel is being referenced—please clarify.

We have reorganized the figure to make clearer which panel is being referenced for Extended Data Fig. 1c (the referenced panel is the schematic diagram of the DNA substrate).

• Figure 1b is referred to before 1a—reorder or correct the reference.

We have fixed this issue by citing Figure 1a (now cited line 103) before Figure 1b (now cited line 117).

• Label nucleic acid polarity in experimental figures (e.g., Fig. 3) and in structural models (e.g., Fig. 5).

We have labeled DNA polarity in experimental figures and structural models.

• Page 4, line 101 should be rephrased for clarity. Lines 115–117 are too long—split into two sentences.

During revisions, we deleted page 4, line 101, which previously read: "How D5 transitions from an auto-inhibited conformation to an activated state in which the ssDNA channel is not obstructed by the primase domains is unknown."

We have revised the long sentence that was previously in lines 115–117 by deleting part of the sentence. This sentence (lines 96–98) now reads: "We use biochemical assays and single-molecule experiments to show that conformational changes that occur when E5 interacts with the polymerase result in helicase activation and enhance E5 primase activity."

• Line 191 and elsewhere: Replace "courses" with "is positioned."

We have made this change to replaced "courses" to "is positioned" and now write, "The presumed lagging strand is positioned within the palm and through a ssDNA binding channel formed by the F8 NTD and Exo domain."

• Lines 209–230: The authors state that the Pol-helicase complex forms only in the presence of DNA (Fig. 1), but order-of-addition data is not provided. Gel shift assays comparing Pol alone, D5 alone, and the complex would strengthen this point, or a citation should be provided if this has been shown previously.

We used mass photometry to show that incubation of E5 and polymerase without DNA does not result in formation of a large complex. The complex only forms efficiently in the presence of DNA (compare Fig. 1c and Extended Data Fig. 1b). These findings are described in lines 141–143 of the manuscript. Although we had used mass photometry experiments rather than gel shift assays, we believe these results show that the complex only forms efficiently in the presence of DNA.

• Lines 252–255: The authors reference mutational analysis of conserved D5 primase residues in the literature. It would be interesting to know whether these residues affect binding and helicase activity, particularly since both Pol and helicase are required for function.

The E5 residues noted in that section (D70, D72, D170, K130, H134, R181, and K187) are in the primase active site and are remote (> 20 Å) from the polymerase–helicase interface (see Figs. 3a and 3b). We would therefore not expect them to affect binding and helicase activity. It is possible that, through allosteric effects, they may influence helicase function in the helicase–primase complex, but because we did not have these mutants on hand to test this notion, we did not perform this additional set of experiments.

• Figure 4: Since polymerase is shown to promote activity in a dose-dependent manner, the concentration of D5 should be included in the figure and legend so readers can assess relative stoichiometry without referencing the Methods.

We have included the concentration of E5 in the figure and figure legend (please note that this is Fig. 3f in the revised manuscript).

Referee #3 (Remarks to the Author):

I co-reviewed this manuscript with one of the reviewers who provided the listed reports.

Referee #4 (Remarks to the Author):

A. Summary - Yu et al. used single particle cryoelectron microscopy to image a complex of MPOX virus proteins. The assemblage comprises the core of the machinery that would be needed to initiate RNA priming and the first steps in DNA synthesis. Perhaps the most important discovery is that the structure shows how a hexamer composed of D5 helicase primase subunits, interacts with an F8/A22/E24 processive polymerase complex. In forming the complex it exposes a channel in the helicase hexamer which then activates the helicase and facilitates primase activity. The work is supported by extensive experimental documentation including mass photometry and helicase activity measurements using an optical tweezer system. Alphafold modeling was also used to extract further insights into the parallels with homologous primer polymerases. Models are proposed to suggest ways in which various structures are loaded, assembled, and elongated.

B. Originality - The paper describes original and significant research. In particular it resolves longstanding controversies over why it's been so difficult to "prove" D5 is a catalytically active helicase. It also provides a solid enzymological explanation for how poxviruses might initiate genome and lagging strand DNA replication.

C. Data and methodology - The conclusions are convincing and supported by reams of state-of-the-art experimental data.

D. Appropriate use... - The authors have taken care not to over interpret their data, informing the reader of situations where the models might fail due to uncertainties in the experimentally-derived structures.

E. Conclusions - The conclusions seem to be sound. Interesting and testable models are proposed.

F. Suggestions - The literature review covers most of the more critical literature relating to poxvirus DNA replication. From the wording, it might be implied that the rolling hairpin model is still the "prevailing model" for virus replication (lines 102-3). Realistically, the Moyer-Graves model is only of historical importance and has long fallen into disfavor for the reasons the authors cite.

We thank the reviewer for bringing up the important point that the rolling hairpin model is only of historical importance and has long been disfavored. We have removed mention of this model from the Introduction and it is no longer mentioned in the manuscript.

Missing from the introduction is the observation that the polymerase-encoded proofreading exonuclease also plays a critical role in catalyzing virus recombination (PMID: 19224992; PMID: 36015016).

We have added to the introduction mention that the polymerase-encoded exonuclease promotes virus recombination, citing PMIDs 19224992 and 36015016, and now write the following in lines 80–83:

“The DNA polymerase of poxviruses is a family B DNA polymerase with canonical fingers, palm, and thumb domains, an N-terminal domain (NTD), and a 3′–5′ proofreading exonuclease (Exo) domain[5]. Polymerase Exo activity also promotes virus genetic recombination[10,11].”

There is an incomplete sentence that mentions the virus ligase (line 111), in that regard there are several papers showing that the SSB (I3 in VACV) is another essential replication protein (e.g. PMID: 29093092).

We have removed the incomplete sentence that mentioned the virus ligase and added a sentence describing that the single-stranded DNA-binding protein I3 is another essential replication protein, citing PMID 29093092 in addition to another paper (PMID: 9525612) (lines 88–89).

“The single-stranded DNA-binding protein I3 is also essential for viral replication[16,17], and poxvirus DNA replication additionally requires either a viral DNA ligase or a host DNA ligase[18].”

The authors describe dimers of F8/A22/E4 heterotrimers as "inactive forms" (lines 126-7). Many replication forks incorporate a pair of DNA polymerases (sometimes different gene products), super assemblages that are collectively responsible for coincident leading and lagging strand synthesis. Is it conceivable that these are actually biologically relevant structures?

We thank the reviewer for bringing up this point. We suspected that the dimers of F8/A22/E4 heterotrimers represented a previously described dimeric state that was visualized in prior structural analysis (PMID: 37075110). Dimerization of F8/A22/E4 occurred through an interface between the F8 thumb domain one of heterotrimer and the A22 protein of the other heterotrimer that would sterically clash with DNA binding. The authors of the prior study thus referred to it as an “autoinhibition state.” We agree that calling it an inactive state is not accurate, as it may be biologically relevant. We have removed the statement that this state is inactive (lines 107–109).

“We also observed a smaller peak possibly representing auto-inhibited dimers of F8/A22/E4 heterotrimers as observed in a prior study (Extended Data Fig.1b)[20].”

G. References - See (F) for a few comments. It has been claimed that poxviruses can recruit PCNA, and so presumably its associated factors, to the factories that are sites of DNA replication (PMID: 28467896).

Despite various efforts this controversial observation has never been replicated. I'd argue that the work described in the current manuscript supports the contention that, with few exceptions (cellular topoisomerase II), poxviruses do actually encode all the core machinery necessary to catalyze DNA replication. There is no need to posit the recruitment of host systems.

We thank the reviewer for bringing up this important point. We have added the following text to the Discussion section of the manuscript (lines 419–422).

"Interestingly, while a prior study suggested that poxviruses can recruit PCNA (and presumably its associated factors) to the factories that are sites of poxvirus DNA replication[39], our working model suggests that, with few exceptions (e.g., cellular topoisomerase II)[40,41], poxviruses encode all of the core machinery necessary to carry out DNA replication."

H Clarity - the manuscript is well written and the figures are nicely organized and well drawn. I did have some trouble orienting myself when inspecting the figures. Admittedly the structure figures are already quite complicated, but could the authors please always tab the 5'- and 3'-ends of the templates and also indicate the N- and/or C-termini of the DNA polymerase where possible? It would help orient the reader.

We thank the reviewer for this suggestion to improve the clarity of the manuscript. Wherever possible for models shown in multiple orientations, we have modified the figures so that the 5′ and 3′ ends of the nucleic acids are labeled. Because the polymerase is heterotrimeric, labeling all N and C termini would pose challenges and potentially overcrowd the figures. We have added additional annotation to Extended Data Fig. 2d to show the positions of the F8 NTD and C-terminal region, which is the F8 thumb.

David Evans

Referee #5 (Remarks to the Author):

Yu et al. provide evidence that supports the hypothesis that the MPXV D5 helicase-primase RNA polymerase and helicase activities are stimulated by the presence of the viral DNA polymerase (F8-A22-D4). This work is of importance because it has been declared an international public health emergency by the WHO. The set of reported structures in this study contrasts differences between previously reported structures in the literature. They resolve the structure of the entire replisome bound to a DNA template using single-particle cryoEM. The structures reveal that the D5 RRM undergoes a significant conformational change in the presence of DNA polymerase, allowing access to the helicase domain. Furthermore, they also provide biochemical and single-molecule data confirming their hypothesis. Overall, the manuscript is well written and provides clear evidence to support the author's hypothesis. We would like the authors to consider a few points outlined below. This work may be suitable for the broad range of readers at Nature; however, several concerns need to be addressed prior to further editorial processing.

Mass photometry data (Figure 1c, extended Figure 1b):

The mass photometry data shows several peaks for the polymerase-only sample. It is unclear which peak corresponds to what because the authors don't state the expected weights for the individual subunits. Please add the expected weights for each subunit to the manuscript. Furthermore, the authors propose that the peak observed at 152 kDa corresponds to the fully assembled polymerase, despite their expectation of a peak at 192 kDa. This would correspond to an error of about 20%. Refeyn reports an expected measurement error of ~5%. How can you explain this discrepancy? Without knowing the expected size of all subunits, it is difficult to judge whether the 152 kDa peak is lacking a

subunit. Additionally, a very small peak is observed at the expected size of ~200 kDa. Could this be the fully assembled polymerase? The peak that the authors suggested to be the polymerase dimer is also ~20% off from the expected size. Please clarify.

We thank the reviewer for noting this issue; this concern was also raised by Reviewer #1.

We have repeated the experiments and believe that the inaccurate mass of 152 kDa for the peak we attributed to the polymerase was due to an issue with instrument calibration. We have since repeated the mass photometry experiments, and observed a peak at 193 kDa, which aligns closely with the expected 191 kDa for the polymerase holoenzyme. We have updated Extended Data Fig. 1b with the new experimental data. We have also included a new table in the manuscript that provides the expected versus observed masses for individual subunits and complexes (see Supplementary Table 1).

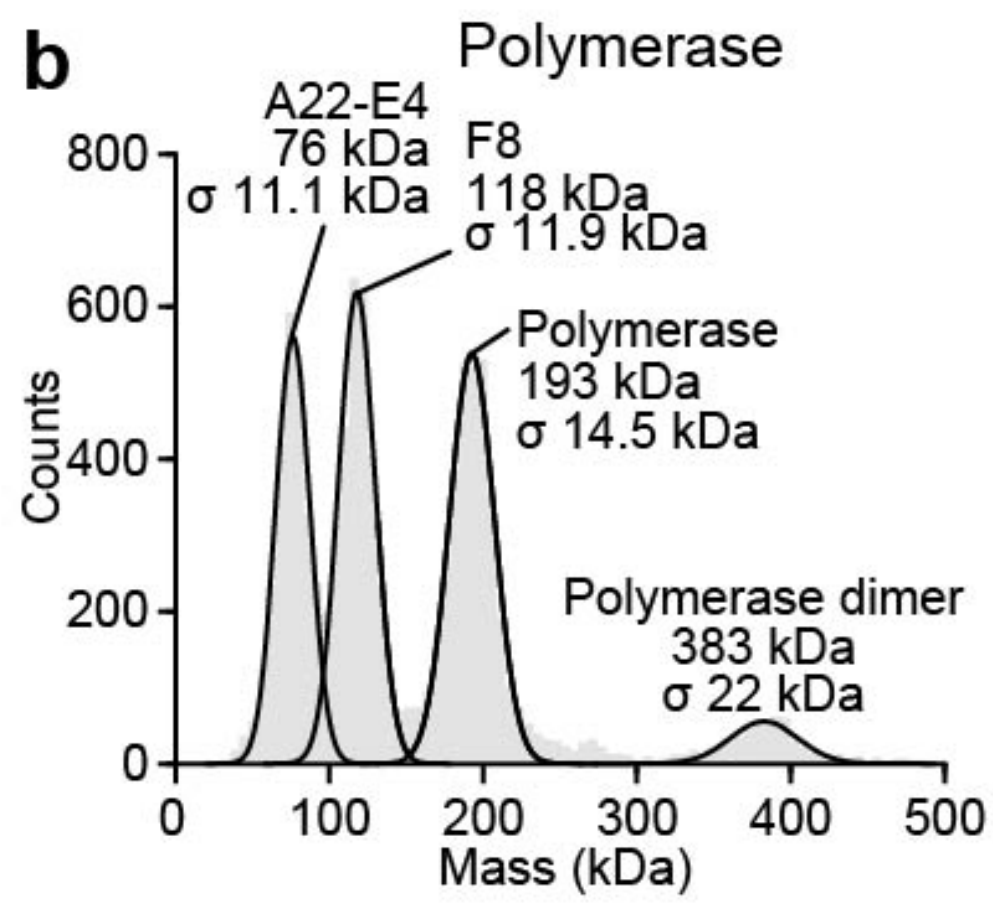

Extended Data Fig. 1b.

b, Mass photometry of the polymerase alone, E5 alone, or E5 and polymerase co-incubated without DNA. The experiment was performed twice (*n* = 2) and representative data are shown.

Mass photometry experiments are not consistent across figures. For example, in Figure 1c, the replisome is estimated to be 680 kDa, but in Extended Data Figure 1, the replisome is now 752 kDa, a 72 kDa difference that is quite significant given the sizes of the smaller components, such as F8/A22/E4. Could the authors rationalise what is causing this? SDS-PAGE of each component will show its purity and help identify any impurities that may cause this.

We thank the reviewer for raising this important issue, which was also brought up by additional reviewers. We believe the issue arose from issues with instrument calibration for that particular experiment. In the revised version of the manuscript, we provide mass photometry data are consistent with the expected molecular weights. These data are provided in Fig. 1c and Extended Data Fig. 1b and summarized also in a new Supplementary Table 1.

Helicase activity (Figure 3c, Extended Data Figure 5):

• Important controls are missing:

o Individual components for Fork DNA (e.g., blue and red ssDNA) – can you stain the gel to show the purity of the other strand?

o Assembled Fork DNA on its own (e.g., without D5 and polymerase)

• What are these intermediate bands that are present? Authors identify the topmost band as Fork DNA but as the reaction progresses, the intensity of the band doesn't visually decrease much. Further quantification of this experiment with properly identified bands and products will be required.

• Authors describe enhanced helicase activity when polymerase is at a 2:1 ratio with D5, given that structurally, it appears as a 1:1 complex. Do the authors have an explanation for this observation?

We thank the reviewer for these constructive comments regarding the helicase assays.

In the revised manuscript, we have provided additional helicase activity assays that include a lane showing the assembled DNA substrate on its own (without E5 or polymerase) (revised Fig. 2b). Regarding the intermediate bands observed beneath the fork DNA, we note that such bands can be observed in similar assays using DNA substrates (e.g., they were also observed by Li et al. in helicase activity assays with MPXV E5, see PMID: 38177671). We believe these could represent minor alternative conformations that are inherent to the DNA substrate preparation. We have not stained the gel to show the purity of the other strand.

In the revised manuscript, we provide a quantification of the results of helicase activity assays and focus the quantification of the ssDNA product (see Fig. 2b and 2c). We agree with the reviewer that the intensity of the fork DNA band does not decrease much, and that this may reflect an incomplete reaction.

While our structural model identifies a 1:1 stoichiometric complex, the enhanced activity observed at a 2:1 polymerase-to-E5 ratio likely reflects the biochemical requirement to drive the binding equilibrium toward full complex assembly in the assay conditions used, considering also complex dissociation.

Consistent with this notion, the results of our single-molecule assays suggest that higher concentrations of polymerase are required for efficient helicase activity and decrease the frequency of pauses in helicase activity, which we attribute to polymerase dissociation from the helicase (see Fig. 2j).

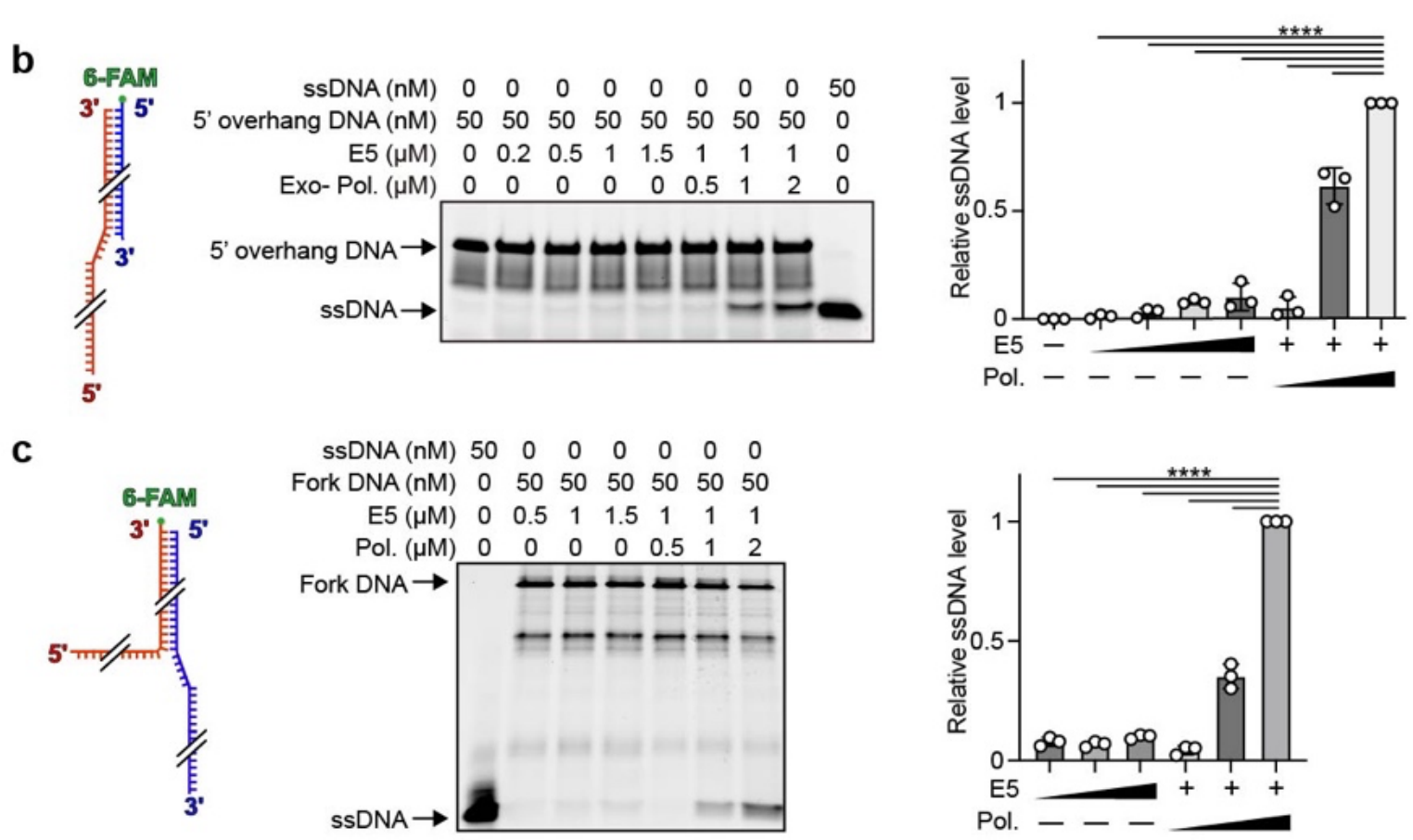

Figure 2. new panels b, c

b, c, Helicase activity assay performed with MPXV E5 alone or co-incubated with polymerase using DNA substrates containing a 5′ overhang (**b**) or a forked-DNA substrate (**c**) visualized with a polyacrylamide-TBE gel. ssDNA control is the unannealed primer. Schematic diagrams of the DNA substrates and quantification of the ssDNA bands are shown. Data are mean ± s.d.; each experiment was performed three times (n = 3), and representative gels are shown. One-way ANOVA with Tukey's multiple comparison test (****P < 0.0001). 6-FAM, 6-carboxyfluorescein. Pol.: polymerase.

Single-molecule experiments (Figure 3d-h, Extended Data Figure 5-7):

A missed opportunity for the single-molecule experiment would have been to correlate fluorescence with the force measurements that are presented. Fluorescent labelling of D5 and polymerase would allow you to correlate complex formation with helicase activity. This would address several questions:

We appreciate the excellent suggestion to correlate fluorescence with force measurements. To address this, we attempted to implement confocal fluorescence microscopy with a LUMICKS C-trap dual-optical-tweezers system. We engineered constructs in which a Halo-tag (34 kDa) was fused to the C terminus of E5 to label the helicase and to the N terminus of the F8 subunit of the polymerase. This approach allowed us to label the E5 helicase with the 549 HaloTag ligand fluorescent and the polymerase with 646 HaloTag fluorescent ligand.

Unfortunately, while in pilot experiments we observed events consistent with E5 and polymerase co-localizing and some activity, we also found that HaloTag labeling of E5 impaired its helicase activity in our single-molecule experiments. We suspect this is because the E5 hexamer could not tolerate the six HaloTags (which would contribute an additional 204 kDa to the complex) where they were positioned. We are now exploring other fluorescent-labeling approaches, but because testing different approaches will require extensive optimization, we plan to pursue these experiments as part of a follow-up manuscript.

Please note, however, that in the comments below, we describe an alternate approach using unlabeled proteins in new single-molecule experiments to answer the concern raised.

• Is the pausing seen (Ext Data 7) due to polymerase dissociation (loss of polymerase fluorescence)?

We thank the reviewer for this important question regarding the origin of the pauses.

We indeed hypothesized that the pausing events were related to polymerase dissociation. Given that we were unable to obtain fluorescently labeled proteins, we decided to test this hypothesis by examining whether the pauses were sensitive to polymerase concentration.

We analyzed the data to specifically quantify the pause fractions (see Fig. 2j) and found that the pause fraction was sensitive to polymerase concentration (see Fig. 2g and 2h). Specifically, the pause fraction decreased from 18.4% at a 1:2 (E5:polymerase) ratio to 8.9% at a 1:10 ratio. Importantly, we also included in new assays an E5 construct that lacks the RRM domains that are important for autoinhibition ($E5^{\Delta RRM}$) (described in PMIDs: 38914567, 38177671). That construct, which would not require polymerase binding for activation, had a pause fraction of only ~1%. Additionally, the pause-free velocity remained relatively similar across all conditions: 49 ± 9 bp s^{-1} (1:2 ratio), 45 ± 3 bp s^{-1} (1:10 ratio), and 34 ± 1 bp s^{-1} for the $E5^{\Delta RRM}$.

These results provide strong evidence that the pausing events are primarily driven by polymerase dissociation.

The new results are presented in Figs. 2e–2k and are described in lines 243–255.

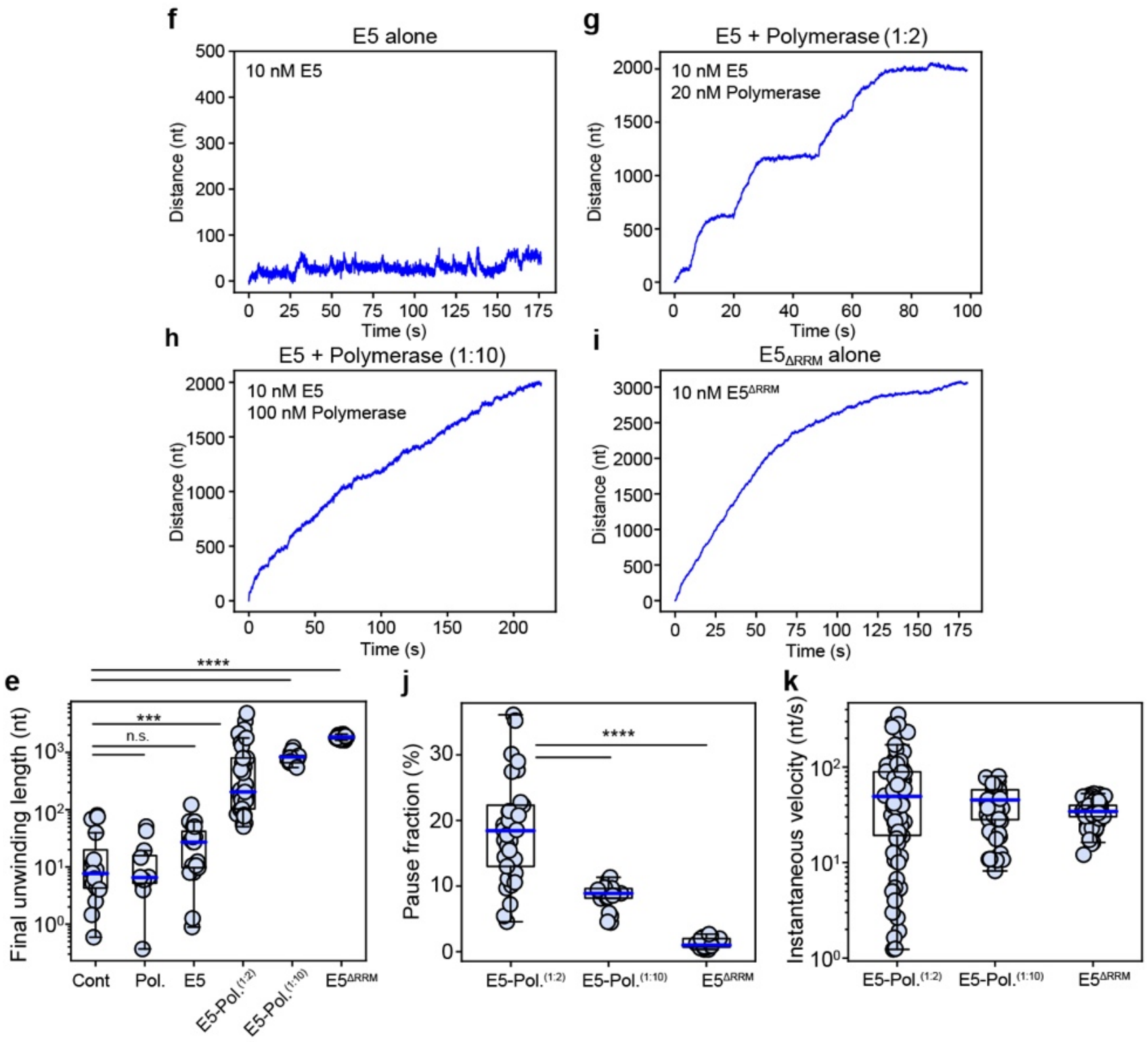

Fig. 2. new panels e—k

e, Distribution of the unwinding length at 60 s time point of a control condition (no proteins, *n* = 16), polymerase alone (*n* = 12), E5 alone (*n* = 19), E5 with polymerase 1:2 ratio (*n* = 33), E5 with polymerase 1:10 ratio (*n* = 19), or $E5^{\Delta RRM}$ (*n* = 25). The box spans the interquartile range (IQR; 25th to 75th percentile), and individual points represent raw data. Blue lines represent median values for different conditions. Statistical comparisons between experimental conditions were performed using Welch's t-test. Not significant (n.s.); ***P = 0.000499; ****P < 0.0001.

f–i, Representative traces of unwinding activity in the presence of 10 nM E5 alone (**f**), 10 nM E5 with 20 nM polymerase (containing Exo-deficient F8) (**g**), 10 nM E5 with 100 nM polymerase (containing Exo-deficient F8) (h), or 10 nM $E5^{\Delta RRM}$ (**i**).

j, Comparison of the distributions of the pause fraction of E5 with polymerase at a 1:2 ratio (*n* = 33), E5 with polymerase at a 1:10 ratio (*n* = 19), or $E5^{\Delta RRM}$ (*n* = 25). The box spans the interquartile range (IQR; 25th to 75th percentile), and individual points represent raw data. Blue lines represent median values for different conditions. Statistical comparisons were performed using Welch's t-test (****P < 0.0001).

k, Comparison of the instantaneous velocity of E5 with polymerase at a 1:2 ratio (*n* = 75), E5 with polymerase at a 1:10 ratio (*n* = 53), or $E5^{\Delta RRM}$ (*n* = 92). The box spans the IQR (25th to 75th percentile) and individual points represent raw data. Blue lines represent median values.

• How many polymerases are present (increased polymerase fluorescence intensity)?

While our mass photometry experiments and structural analysis both suggest that E5 and the polymerase assemble with a 1:1 stoichiometry, our inability to obtain fluorescently labeled proteins prevented us from directly addressing this question as part of the revisions.

• How likely is D5 alone to unwind (Ext Data 5 shows some traces that are active)? Is this actually D5 unwinding, or is it just the melting of the DNA junction?

In biochemical assays, we did observe evidence of weak E5 helicase activity with E5 alone. This is consistent with some traces showing detectable activity (see Extended Data Fig. 8b). We added a control condition in which the DNA was maintained at the same force with no protein present (see Fig. 2e and Extended Data Fig. 8a). Please see the comment below regarding the point about melting of the DNA junction.

• Control experiments of holding the DNA alone with no proteins present will clarify if it is spontaneously melting at such high forces.

As noted above, we performed control experiments at 52 pN in the absence of protein. Under these conditions, we did not observe evidence of spontaneous DNA melting (see Fig. 2e, along with Extended Data Fig. 8a for the associated traces). These findings suggest that there is no spontaneous melting at the forces we used in experiments.

Helicase activity has been previously reported at lower forces (~20 pN) – Burnham et al. (2019) Nat Comms [PMID: 31089141]. Could authors explore the force dependence for helicase activity?

We explored the activity of the E5–polymerase complex at lower forces. We performed 10 independent replicates each at 5 pN and 20 pN with an E5-to-polymerase ratio of 1:2. Under these lower forces, we did not observe discernible helicase activity or DNA unwinding (see Extended Data Fig. 9c,d).

• Are the higher forces presented in this study physiologically representative of MPX replication?

This is an important question. The in vitro system we are using here involves purified components and importantly lacks additional factors, including the poxviral single-stranded DNA-binding protein I3. We suspect that the single-stranded DNA-binding protein (and perhaps other viral factors) may influence the forces involved in replication in cells. As such, we would not assume that the forces used in our study represent physiologically relevant forces. Precisely mimicking the environment inside infected cells would prove challenging but would be required. We do plan to test the role of adding the single-stranded DNA-binding protein I3 to the system to determine if this protein alters the forces required to observe unwinding activity, as well as testing different substrates (e.g., containing ssDNA overhangs). However, we believe these experiments would be beyond the scope of our current study.

In another study, we also examined the helicase–primase of herpes simplex virus 1 (HSV-1) using similar experiments with the LUMICKS C-Trap and found an optimal force of 45 pN in those assays (PMID: 41468884). We would not extrapolate that this force would be representative of HSV-1 replication, but the forces required to see robust activity in those assays are similar in scale to the force we had to use here with the MPXV helicase–primase.

To address this important limitation, we have added the following text to the Discussion section (lines 411-418):

“A limitation of the study is that the force we used to tether DNA in the single-molecule assays (52 pN) is high. However, we did not observe discernable helicase activity at lower, more physiologically relevant forces (5 and 20 pN) we also tested. Importantly, control experiments in which DNA was held at that 52 pN showed no evidence of spontaneous DNA melting. We believe that the higher forces may lower the energy barrier for viral helicase activity under these experimental conditions. Future work will be required to determine whether using a forked DNA substrate or adding the poxviral single-stranded DNA-binding protein influences helicase efficiency.”

• Would you observe helicase activity at low forces (5 pN)?

We observed no discernible unwinding activity at 5 pN force (see Extended Data Fig. 9c).

• If you utilise a fork DNA substrate as the ensemble experiment, would you see helicase activity at low forces (5 pN)?

Unfortunately, although we recognize the potential impact of DNA substrate architecture on helicase activity, our single-molecule experiments using the LUMICKS C-Trap are currently constrained to commercially available ssDNA/dsDNA hybrid substrates. In response to another reviewer's comments (see Reviewer #1 comment 4), however, we performed ensemble helicase assays with substrates that contain a fork or a 5′ overhang (see revised Figs. 2b and 2c). These experiments show that the helicase is robustly activated by the polymerase on both forked substrates and substrates that contain a 5′ overhang. We agree that examining additional substrate architectures in single-molecule assays would be valuable, but we believe this analysis would be beyond the scope of the current study.

Line 216: "Unwinding experiments were conducted in a constant-force mode with a high-frequency feedback system adjusting the position of a bead to maintain the desired force set point (usually 45–52 pN)." Can authors clarify why this range was used?

We thank the reviewer for identifying this issue. The force we used was 52 pN, and the text has been corrected accordingly. The revised sentence in lines 229–231 now reads:

"We conducted unwinding experiments in a constant-force mode with a high-frequency feedback system adjusting the position of a bead to maintain the desired force set point (52 pN)."

As noted above, 52 pN is the force at which we observed robust helicase activity, and we did not observe any spontaneous DNA melting at this force.

Line 223: "…no evident helicase activity was observed across all examined traces (n = 23)…" Examining the extended Figure 5, it appears that the helicase on its own exhibits some minor helicase activity. In my opinion, this is still in line with your overall hypothesis and worth mentioning. I would replace "no evident helicase activity" with "minor helicase activity" or something similar. You could also elaborate on the limited processivity of that helicase activity (e.g., a maximum of 300 bp unwound).

We agree that characterizing this as "minor activity" provides a more accurate representation of the data.

The revised text in lines 236–237 now reads:

"In experiments containing MPXV E5 alone, we only observed minor helicase activity across all examined traces (*n* = 19)"

In line 227: the statement "67 examined events from 29 traces" is unclear. What do the authors classify as an event? Are they separated by long pauses? And if yes, how long does the pause have to be for it to be a separate event?

We have updated the manuscript to provide quantitative analysis and to specify the criteria for defining events and pauses in helicase activity. These are detailed in the Methods section (lines 1110–1122).

Briefly, analysis was conducted using custom Python scripts, in which raw traces were converted into nucleotides unwound. To identify pauses, the data were smoothed using a Savitzky–Golay filter, and we calculated the discrete derivative of unwinding over time. We applied a velocity threshold of 2 nt s^{-1}; any segments falling below this threshold were classified as a "pause." Unwinding segments separated by these identified pauses were counted as distinct events within a single trace. For consistency, the first 60 seconds of each unwinding trace were used to quantify pause durations and frequencies. We have updated the text to ensure the distinction between unwinding events and objectively defined pause states is clear.

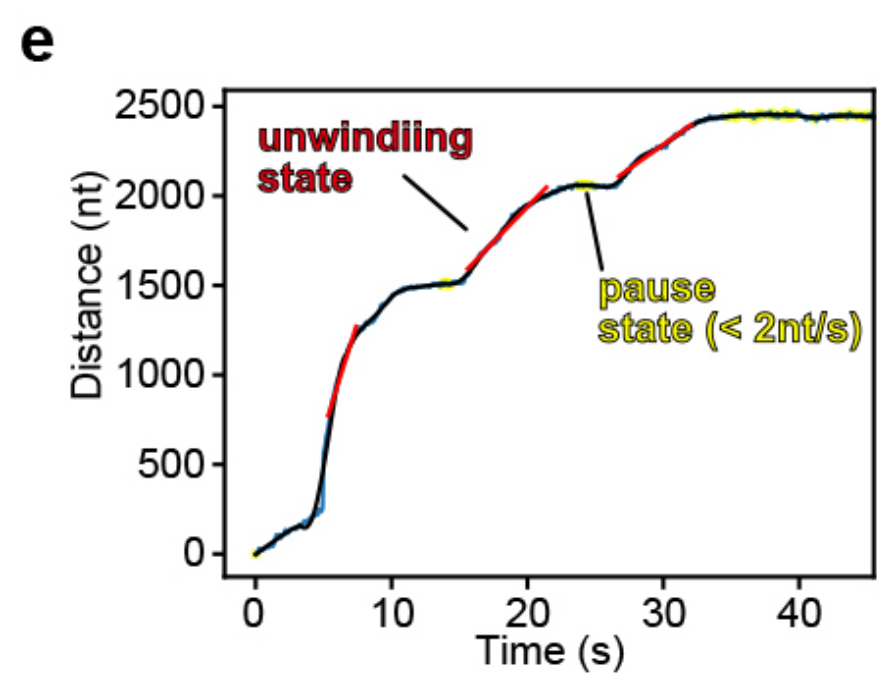

Extended Data Fig. 8e.

e, Representative trace of optical tweezer data analysis. The paused and unwinding states are labeled after classification based on the instantaneous unwinding rate (see Methods). The original trace is from an experiment that included E5 and polymerase (Exo-deficient) at a 1:2 ratio.

We found the mechanism figure unclear:

• Re-order the figure in a more linear format because it is unclear where the mechanism starts at first glance • d-f seems to be the mechanism, utilise a subtle box to indicate this

We have reordered the figure panels to follow a clear chronological path of the replication process. The original panels Figs. 5d and 5f, which illustrate the proposed working model, have been refined and integrated into the revised Figs. 5e and 5f.

• g-h comparisons should be moved to the Extended Data Figure for clarity

As suggested, to improve the clarity of the figures, we have moved the previous panels Fig. 5g and 5h to Extended Data Figs. 11g and 11h.

Minor comments:

• Line 111: The sentence wasn't finished.

Thank you. We have corrected this issue during revisions.

• Fig. 3g: There is a typo: it should be 10 nM D5 and not 10nm.

We appreciate the reviewer's careful reading. The typographical error in Fig. 3g has been corrected from "10nm" to "10 nM" in the revised Fig. 2f.

• Line 781: You probably meant Pluronic F-127. (Pluronics F128 does not exist.)

We thank the reviewer for identifying this error. We have corrected this typographical error and write Pluronic F-127 in the revised manuscript.

• Across the manuscript, distances aren't clearly defined. For example, page 8, lines 202-203 says: "The D5A RRM is displaced by 85 Å to interact with the F8 thumb, and the D5F RRM is 203 displaced by 70 Å to interact with A22." – are these RMSDs? Clarify these instances across the manuscript.

We appreciate the reviewer identifying this ambiguity regarding structural distances. We have clarified that these values refer to center-of-mass displacements rather than RMSD.

While the updated values are more accurate, they remain consistent with our structural model and do not alter the biological conclusions. The revised manuscript now explicitly states (lines 198-199):

“The $E5_A$ RRM center-of-mass is displaced by 58 Å to interact with the F8 thumb, and the $E5_F$ RRM center of mass is displaced by 54 Å to interact with A22.”

• Figure 3c: clearer indication of FAM positioning on the substrate – e.g., FAM could be in green and a bit larger.

We thank the reviewer for this suggestion. We have revised Fig. 3c to provide a clearer indication of the FAM label. In the updated Figs. 2b, and 2c, the FAM label is now green and is larger.

• Page 9, line 226: “we observed rapid DNA unwinding events at an average rate of 46 bp s-1” – Comparison to rates of other replicative helicases would be required to justify why the authors determine the MPXV helicase to be rapid.

In the revised manuscript, we have removed the descriptor "rapid," as we were not aiming to compare the observed rates to those of other replicative helicases.

• Ensemble and single-molecule experiments use concentrations across orders of magnitude. For example, in bulk, helicase activity requires µM concentrations, but in the single-molecule experiment, it can be observed at nM concentrations. Can authors justify this?

We believe that the apparent difference in the amount of E5 required between the two assays primarily reflects differences in the effective DNA substrate concentration and in the sensitivity of the single-molecule assays. In the helicase assays, the DNA substrate is present at ~50 nM, whereas in the optical tweezer measurements, a single DNA molecule (~2 pM in the channel) is held between the beads for each measurement. Because each optical tweezer trace involves capturing one DNA molecule and transferring it into the protein-containing channel, the local DNA substrate concentration is orders of magnitude lower than in bulk assays, thereby requiring substantially fewer E5 molecules to observe unwinding activity.

Referee #6 (Remarks to the Author):

(Per the editor’s request, I have focused my comments on the single-molecule experiments in this study.)

In this manuscript, Yu et al. presented cryo-EM structures of the DNA-bound MPXV replisome in pre-initiation and RNA elongation states. Compared to a previous structure of the helicase-primase (D5) in isolation, these new structures showed that D5 undergoes a large conformational change upon binding to the polymerase, exposing the ssDNA entry channel within the helicase. In addition, they performed bulk helicase assays and single-molecule optical tweezers experiments, both showing robust unwinding activities only with the helicase-polymerase complex but not with the helicase alone. These results nicely corroborate the structural data and thus are a valuable addition to the study. However, the single-molecule data need further analysis to be fully validated.

Major points:

(1) The unwinding traces were obtained at 52 pN of force. This is an unusually high force, almost reaching the overstretching regime of dsDNA. The authors need to provide evidence that the helicase-polymerase complex can unwind DNA at a lower force or explain why such a high force is required.

The reviewer raises an important point regarding the forces used in single-molecule experiments, a concern that was also raised by Reviewer #5. We had attempted to use lower forces; however, after testing different forces, we were only able to observe robust helicase activity at 52 pN with the substrate used in these assays.

To address this concern, we conducted additional experiments to confirm that no spontaneous melting occurs at 52 pN. Experiments conducted at 52 pN in the absence of protein showed no spontaneous DNA unwinding or mechanical rupture. Second, we tested the E5-polymerase complex at lower forces (5 pN and 20 pN) and found no discernible helicase activity, indicating that these tensions are insufficient for the E5–polymerase complex to initiate unwinding on the specific DNA substrate used in these assays. Our current hypothesis is that the higher forces lower the energy barrier for unwinding. This in vitro system also does not contain the poxviral single-stranded DNA-binding protein I3, which could influence helicase efficiency. Also, due to design of the commercially available substrate we used on the LUMICKS C-Trap instrument, we could not test whether a forked DNA substrate would require less force to unwind.

Nonetheless, despite the high forces required for the single-molecule experiments, the assay results, supported by multiple controls, robustly demonstrate that MPXV polymerase binding activates E5.

We also note that in our work on a separate system (the herpes simplex virus 1 helicase–primase complex) we used a similar substrate and LUMICKS C-Trap instrument and observed helicase activity at 45 pN (PMID: 41468884). Future work will be required to determine whether using a forked substrate or including viral single-stranded binding-proteins alters the forces required for unwinding.

We have added the following text to the Discussion section (lines 411–418):

“A limitation of the study is that the force we used to tether DNA in the single-molecule assays (52 pN) is high. However, we did not observe discernable helicase activity at lower, more physiologically relevant forces (5 and 20 pN) we also tested. Importantly, control experiments in which DNA was held at that 52 pN showed no evidence of spontaneous DNA melting. We believe that the higher forces may lower the energy barrier for viral helicase activity under these experimental conditions. Future work will be required to determine whether using a forked DNA substrate or adding the poxviral single-stranded DNA-binding protein influences helicase efficiency.”

(2) Line 222: “In experiments containing MPXV D5 alone, no evident helicase activity was observed across all examined traces…” However, some traces in Extended Data Fig. 5 clearly exhibited limited unwinding. This is different from the polymerase alone which showed no activity (Extended Data Fig. 6). This distinction should be stated. Presumably these data were also collected at 52 pN of force. These constructs should be compared at a more physiological force.

We thank the reviewer for bringing up this concern, which was also raised by Reviewer #5. We agree that some traces had limited unwinding. In the revised manuscript, we have used the term Reviewer #5 suggested ("minor helicase activity") to more accurately describe the behavior of E5 alone, particularly as compared to the complete lack of activity observed with the polymerase alone. These assays were indeed performed at 52 pN of force. Additionally, we have tested the E5-polymerase complex at 5 pN and 20 pN and observed no activity (see Extended Data Fig. 9c,d).

(3) The example traces suggest that unwinding is not a uniform process, frequently interrupted by pauses (Extended Data Fig. 7). What is the origin of these pauses? Is the reported unwinding rate (46 bp/s) pause-free velocity?

We thank the reviewer for this excellent question, which was also raised by another reviewer. We believe that the pauses are related to events during which the polymerase dissociates from E5.

We reached this conclusion by testing the effect of polymerase concentration on the pausing events. Measuring the frequency of pauses and comparing E5-to-polymerase ratios of 1:2 and 1:10, revealed that the pauses are sensitive to polymerase concentration and decrease as the polymerase-to-E5 ratio increases. Notably, we also used a mutant E5 in which the RRM domain is deleted ($E5_{\Delta RRM}$ mutant, PMID: 38177671). $E5_{\Delta RRM}$ is known to be active as RRM deletion removes autoinhibition. Consistent with the notion that the pauses are primarily related to polymerase dissociation events, $E5_{\Delta RRM}$ had minimal pausing in assays.

Furthermore, we found that the pause-free velocity remains relatively consistent across all conditions. These results provide strong evidence that the observed pauses are primarily driven by the stochastic dissociation of the polymerase from the E5 complex. These additional data are presented in Figs. 2e–k and discussed in the manuscript (lines 243–254).

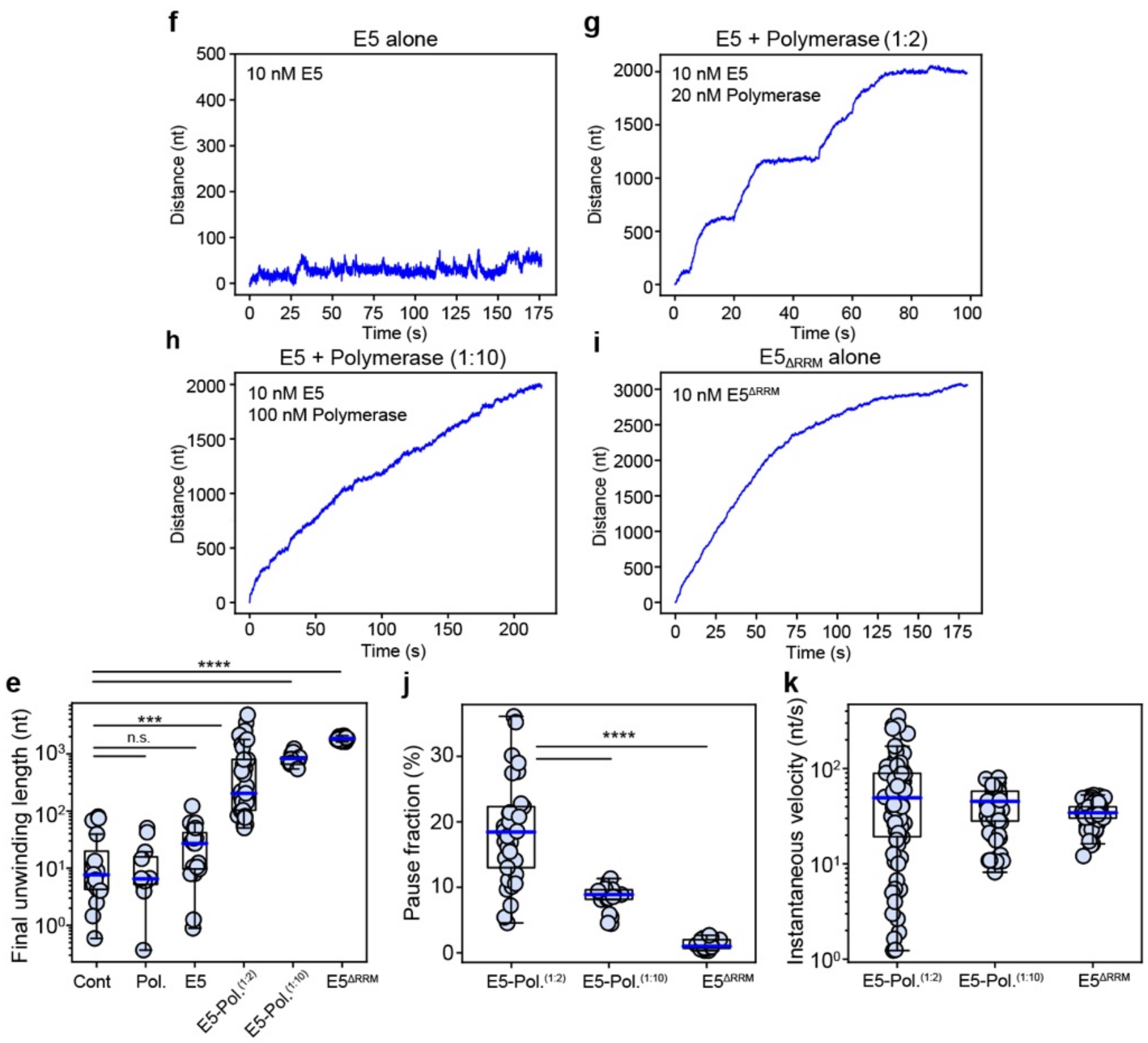

Fig. 2. new panels e—k

e, Distribution of the unwinding length at 60 s time point of a control condition (no proteins, *n* = 16), polymerase alone (*n* = 12), E5 alone (*n* = 19), E5 with polymerase 1:2 ratio (*n* = 33), E5 with polymerase 1:10 ratio (*n* = 19), or $E5^{\Delta RRM}$ (*n* = 25). The box spans the interquartile range (IQR; 25th to 75th percentile), and individual points represent raw data. Blue lines represent median values for different conditions. Statistical comparisons between experimental conditions were performed using Welch's t-test. Not significant (n.s.); ***P = 0.000499; ****P < 0.0001.

f–i, Representative traces of unwinding activity in the presence of 10 nM E5 alone (**f**), 10 nM E5 with 20 nM polymerase (containing Exo-deficient F8) (**g**), 10 nM E5 with 100 nM polymerase (containing Exo-deficient F8) (h), or 10 nM $E5^{\Delta RRM}$ (**i**).

j, Comparison of the distributions of the pause fraction of E5 with polymerase at a 1:2 ratio (*n* = 33), E5 with polymerase at a 1:10 ratio (*n* = 19), or $E5^{\Delta RRM}$ (*n* = 25). The box spans the interquartile range (IQR; 25th to 75th percentile), and individual points represent raw data. Blue lines represent median values for different conditions. Statistical comparisons were performed using Welch's t-test (****P < 0.0001).

k, Comparison of the instantaneous velocity of E5 with polymerase at a 1:2 ratio (*n* = 75), E5 with polymerase at a 1:10 ratio (*n* = 53), or E5$^{\Delta RRM}$ (*n* = 92). The box spans the IQR (25th to 75th percentile) and individual points represent raw data. Blue lines represent median values.

Minor points:

(1) D5 without the primase domain is supposed to be active in unwinding. It would be valuable to conduct single-molecule experiments with this construct if it's available.

We thank the reviewer for this excellent suggestion, which was also raised by other reviewers. As noted above, we have generated an E5 primase-domain deletion construct (E5$^{\Delta RRM}$) and evaluated its activity using single-molecule optical tweezers assays (see Fig. 2e,i–k) and in biochemical assays (see Extended Data Fig. 7a). We have added new data in these figures demonstrating that E5$^{\Delta RRM}$ is active in assays even in the absence of polymerase and has minimal pausing during unwinding.

(2) Line 225: "with both D5 and polymerase present, we observed rapid DNA unwinding events … with a markedly greatefr extent of DNA unwinding compared to D5 alone." The maximum unwinding distance for these conditions should be quantified.

We appreciate the reviewer's request for quantitative comparison of the maximum unwinding distances. We have quantified the unwinding activity across all conditions (see revised Fig. 2e).

Referees' comments:

Referee #2 (Remarks to the Author):
The authors have done an admirable job addressing my concerns regarding validating the structures with mutational studies. In addition, they have increased the rigor in their analysis, validating it with figure/densities. Statistical/validation analyses are also now complete. I recommend publishing and commend the authors on a much improved and impactful manuscript.

Referee #2 (Remarks on code availability):
I did not test the code for the C-trap analysis.

Referee #3 (Remarks to the Author):
I co-reviewed this manuscript with one of the reviewers who provided the listed reports.

Referee #4 (Remarks to the Author):
The authors have addressed my earlier comments and concerns in a satisfactory manner. A description of any associated statistics has been provided in all of the figure legends.

Referee #5 (Remarks to the Author):
The authors have done a great job addressing our concerns.
However, there are a few (minor) comments we would like to add.

Supplementary Table 1: For someone who isn't familiar with the field, it would be helpful to also include the stoichiometry of the subunits and clarify the ratio of each subunit in the complex. This would help with help the reader understand how these masses were determined.

We thank the reviewer for this suggestion. We have revised Supplementary Table 1 accordingly by adding a new column entitled "Expected stoichiometry." This column specifies the expected stoichiometry of the subunits in each target complex. We believe this addition will make the table more accessible to readers who are less familiar with the system.

Supplementary Table 1. Expected masses of subunits and complexes and mass photometry results.

**Complex**	**Subunit masses**	**Expected stoichiometry**	**Expected Complex mass**	**Measured mass**
MPXV E5 helicase–primase	90 kDa (E5)	Hexamer	540 kDa	478 kDa s.d. of ± 30 kDa
MPXV polymerase (F8/A22/E4)	117 kDa (F8)<br>49 kDa (A22)<br>25 kDa (E4)	1:1:1 ratio of F8:A22:E4	191 kDa	193 kDa s.d. of ± 14.5 kDa
DNA-bound MPXV helicase–primase and polymerase (E5+F8/A22/E4) + DNA template 1	90 kDa (E5)<br>117 kDa (F8)<br>49 kDa (A22)<br>25 kDa (E4)<br>25 kDa (DNA)	6:1:1:1 ratio of E5:F8:A22:E4	756 kDa	726 kDa s.d. of ± 53 kDa

Figure 2: The abbreviations in the figure should be defined/included in the caption. E.g. is cont the DNA-only control?

We thank the reviewer for raising this concern. The condition was a control with no protein, so we have fixed the annotation on the graph to instead write “No protein”. We have also updated the figure legend accordingly.

Referee #6 (Remarks to the Author):

The authors have done substantial work during the revision process, which significantly improved the manuscript. Technical difficulties associated with the single-molecule experiments, including fluorescent labeling without interfering with activity, use of a forked DNA substrate, and detecting activity at lower force in the presence of single-stranded DNA binding protein, left this part still somewhat unsatisfactory. Nonetheless, I still think it is a valuable complement to the structural work and together they represent a significant advance in the understanding of this important viral machinery. I do request two additional experiments that could further strengthen the manuscript:

(1) Compare the kinetics of DNA unwinding between 5' overhang and forked DNA substrates in the bulk helicase assay (Fig. 2b, c). Currently only the final normalized ssDNA levels are presented, which do not fully support the conclusion that DNA geometry does not affect the helicase activity.

We thank the reviewer for this suggestion. To directly compare the kinetics of DNA unwinding between the two DNA geometries, we have performed time-course bulk helicase assays using both the 5′-overhang DNA substrate and the forked DNA substrate under the same reaction conditions. The revised Extended Data Fig 7a,b now includes the results of these experiments, with a representative gel and quantification from three independent experiments.

In the updated data, both substrates showed time-dependent accumulation of ssDNA product in the presence of MPXV E5 and the polymerase complex. These results indicate that MPXV E5, when co-incubated with the polymerase complex, unwinds both 5′-overhang and forked DNA substrates with generally similar kinetics (see Extended Data Fig. 7a,b).

We have revised the relevant text and now state that (lines 218–221):

“Additionally, in assays with the DNA substrate containing a 5′-overhang or a forked DNA substrate, we observed similar unwinding activity of E5 together with polymerase under the experimental conditions we examined (Extended Data Fig. 7a,b).”

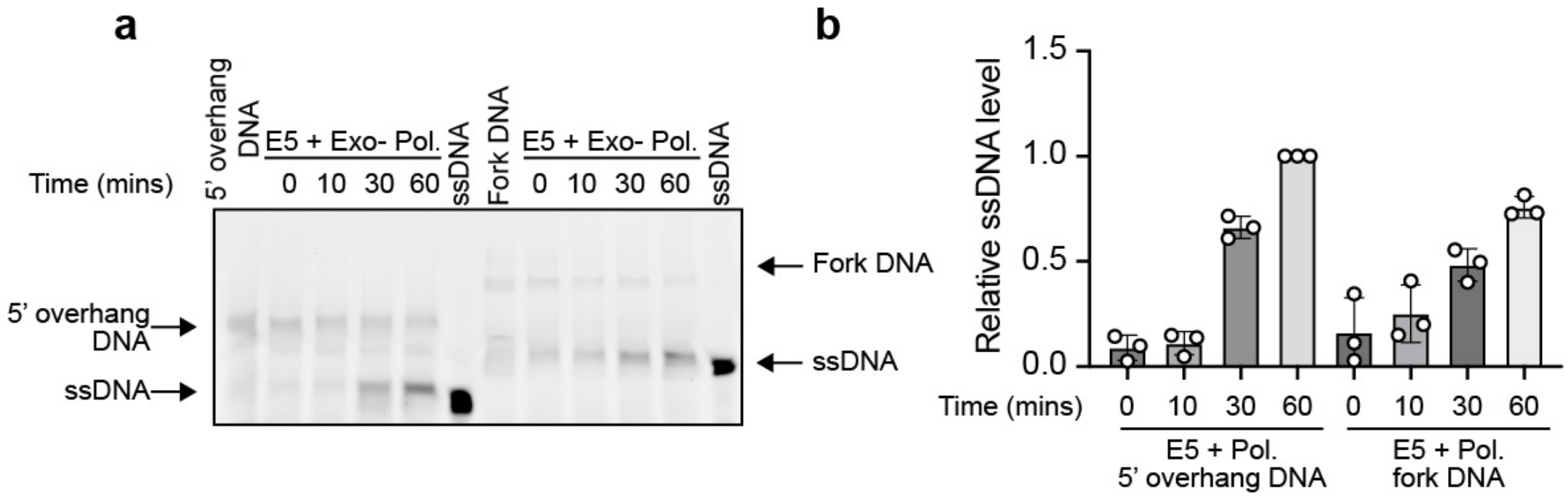

Extended Data Fig. 7 new panels a,b

a,**b**, Time-course helicase activity assay performed with MPXV E5 co-incubated with polymerase using DNA substrate with a 5′ overhang or a fork DNA substrate (**a**). Quantification of the ssDNA, with mean ± s.d., is shown (**b**). This experiment was performed three times (*n* = 3), and a representative gel is shown.

(2) Given the higher activity of the E5dRRM construct, it should be tested further in the single-molecule assay to see if unwinding can be observed at lower forces.

We have now tested the E5$^{\Delta RRM}$ mutant in the single-molecule unwinding assay at lower forces of 5 pN and 20 pN. Compared with the wild-type E5 polymerase in the presence of polymerase (1:2 ratio), E5$^{\Delta RRM}$ showed activity at these lower forces; however, E5$^{\Delta RRM}$ has substantially stronger unwinding activity at 52 pN than at 5 or 20 pN. These results indicate that although the RRM deletion enhances unwinding at lower forces, that elevated forces are still required to observe robust E5$^{\Delta RRM}$-mediated DNA unwinding. We have added these data to Extended Data Fig. 10 and revised the manuscript accordingly (lines 433–435).

“Additionally, with the more active E5$^{\Delta RRM}$ construct, we observed weaker activity at 5 and 20 pN (Extended Data Fig. 10).”

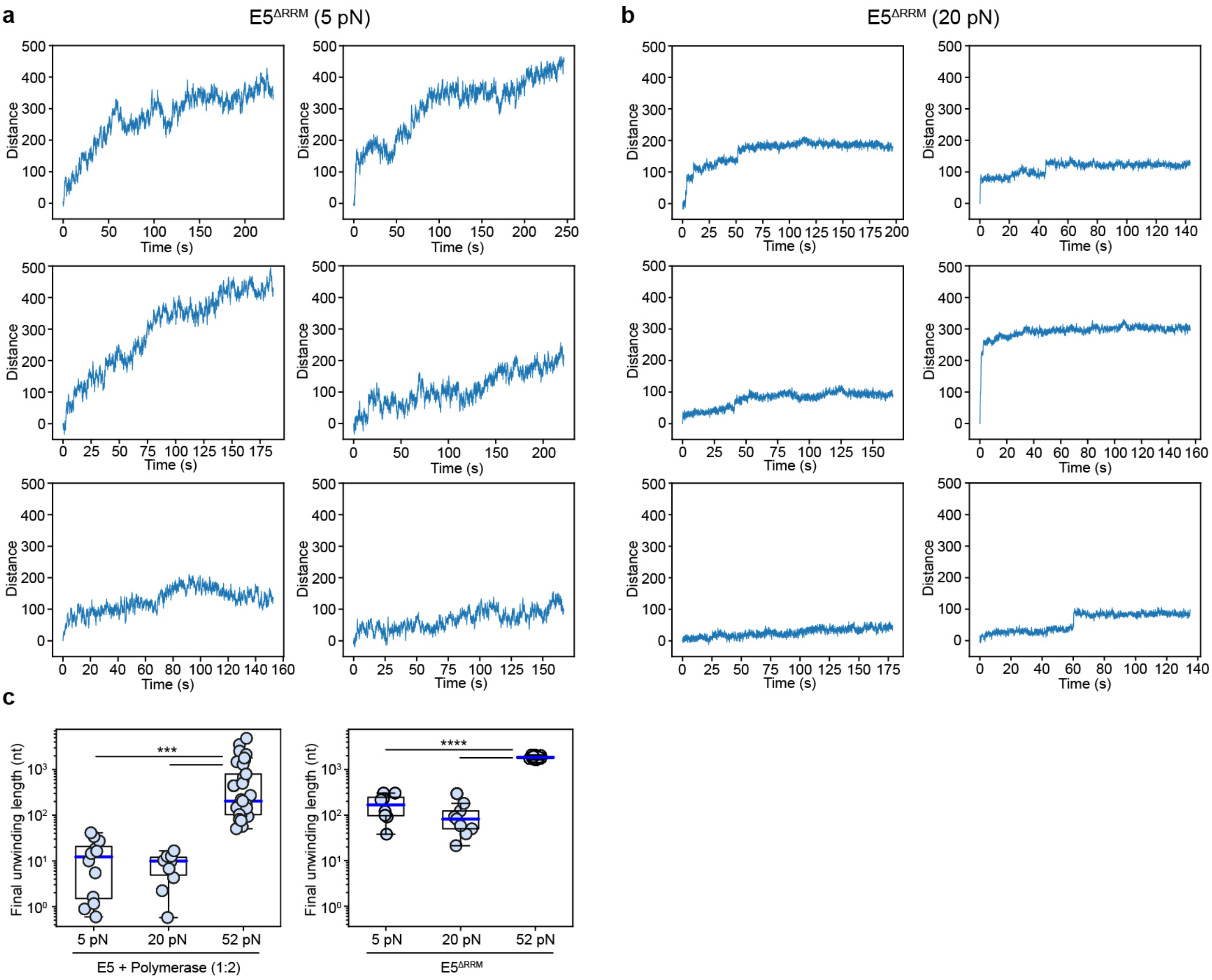

Extended Data Fig. 10. Optical tweezer traces for experiments performed with E5$^{\Delta RRM}$ at lower forces.

a, Representative traces of unwinding activity in the presence of 10 nM E5$^{\Delta RRM}$ measured at 5 pN.

b, Representative traces of unwinding activity in the presence of 10 nM E5$^{\Delta RRM}$ measured at 20 pN.

c, Distribution of the unwinding length at 60 s time point of a E5 with polymerase 1:2 ratio or E5$^{\Delta RRM}$ at 5 pN, 20 pN and 52 pN. The box spans the interquartile range (IQR; 25th to 75th percentile), and individual points represent raw data. Blue lines represent median values for different conditions. Statistical comparisons between experimental conditions were performed using Welch’s t-test. ****$P < 0.0001$, ***$P = 0.000452$ (5 pN) and 0.000416 (20 pN), respectively.

Referee #7 (Remarks to the Author):
I co-reviewed this manuscript with one of the reviewers who provided the listed reports.
